# Photocatalytic *E*→*Z Contra*‐Thermodynamic Isomerization of Vinyl Silanes with Lewis Base

**DOI:** 10.1002/chem.202201514

**Published:** 2022-07-22

**Authors:** Thi Minh Thi Le, Thibaud Brégent, Philippe Jubault, Thomas Poisson

**Affiliations:** ^1^ Normandie Univ. INSA Rouen UNIROUEN CNRS COBRA (UMR 6014) 76000 Rouen France; ^2^ Institut Universitaire de France 1 rue Descartes 75231 Paris France

**Keywords:** alkenes, BINAP, isomerization, photochemistry, silicon

## Abstract

Herein, we disclosed the *contra*‐thermodynamic *E*→*Z* isomerization of alkenyl silanes, according to the in situ formation of a chromophoric species, in the presence of *rac*‐BINAP as the catalyst. The reaction carried out in DMSO or CH_3_CN under irradiation at 405 nm allowed the interconversion of the *E*‐isomers into the Z‐congeners in good to excellent yields and outstanding *Z*/*E* selectivities, on 18 examples. Finally, the mechanism of this *E*→*Z* isomerization was studied to get insight into the reaction mechanism.

## Graphical abstract

**The contra‐thermodynamic** *E*→*Z* isomerization of vinyl silanes was reported using *rac*‐BINAP as the photocatalyst. This reaction proceeds via the in situ formation of a chromophoric species, in the presence of catalyst. Under irradiation at 405 nm the interconversion of *E*‐isomers into the Z‐congeners were carried out in good to excellent yields and outstanding *Z*/*E* selectivities (18 examples) and the reaction mechanism was studied.

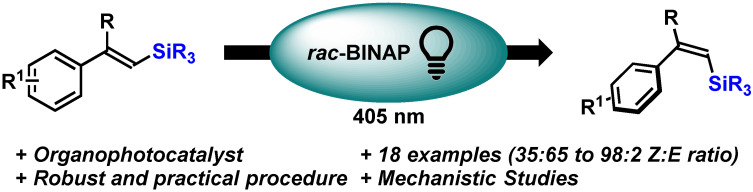

## Introduction

Alkenes are privileged motifs in organic synthesis and have a pivotal role in the elaboration of complex molecules. Moreover, myriad of natural products and bioactive molecules contain an olefinic residue. Therefore, their synthesis and the elaboration of stereospecific access to either the *E*‐ or the *Z*‐isomer are of paramount importance. Thus, organic practitioners devised specific strategies, such as olefinations (e.g. Wittig, Julia‐Kocienski, Horner‐Wadsworth‐Emmons) or stereocontrolled metathesis reactions,^[1]^ for instance, to forge alkenes in a stereospecific manner.

The geometric alkene isomerization is an attractive complementary approach, embracing the principle of atom economy.^[2]^ Whilst the thermodynamically favored *Z*→*E* isomerization has been widely documented, the *contra*‐thermodynamic *E*→*Z* isomerization remained challenging due to the *“uphill”* energetics of the whole process. This endergonic process could be circumvented under the auspice of the excited state reactivity, through the direct irradiation of the olefin or its photosensitization via energy transfer (EnT). This latter strategy pioneered in the 60s’ enjoyed a renaissance over the last ten years under the notable impetus of Gilmour, and others.^[3]^ Alternatively, a complementary approach based on the in situ formation of a chromophoric species, resulting from the interaction of the alkenes with an appropriate catalyst, might also permit the *contra*‐thermodynamic *E*→*Z* isomerization. This strategy, widely used in photoinduced asymmetric transformations^[4]^ and pioneered by F. D. Lewis in the 80s,^[5]^ allowed us to develop recently the photocatalytic *E*→*Z* isomerization of α‐substituted cinnamate derivatives and β,β‐disubstituted vinyl boronates.^[6]^

As part of the portfolio of the versatile olefinic building blocks, alkenyl silanes is a prominent class of substrates with applications in benchmark transformations.^[7]^ The stereoretentive nature of these reactions, allowing a perfect control of the two‐dimensional space, highlights the strategic position of alkenyl silanes in the arsenal of the organic chemists.

**Scheme 1:**
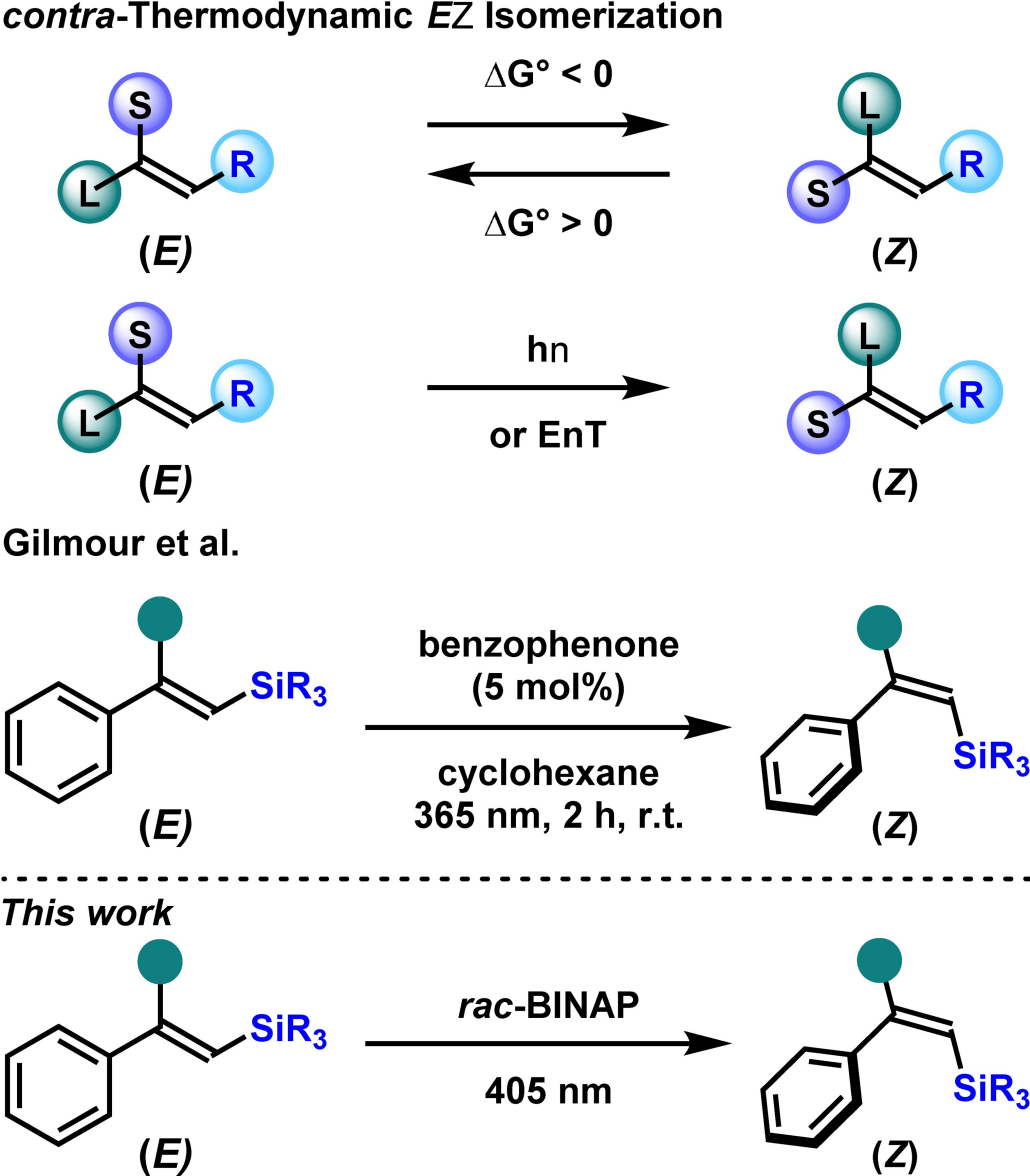
State of the art and present work.

Although the synthesis of di‐ or tri‐substituted *E*‐alkenyl silanes is widely documented,^[8]^ the synthesis of the *Z*‐isomer is more tedious and mostly focused on the di‐substituted congeners.^[9]^ In that context, the interconversion of the *E*‐isomers into the corresponding *Z*‐ones is an appealing strategy. With regards to the synthesis of *Z*‐alkenyl silanes from the corresponding *E*‐isomers, Gilmour disclosed the unique report of a photoinduced *contra*‐thermodynamic *E*→*Z* isomerization based on an EnT using benzophenone, as the photosensitizer, under irradiation at 365 nm.^[10]^ Complementary to this pioneer report, we sought to develop a straightforward protocol to forge the versatile *Z*‐alkenyl silanes through the in situ formation of a transient chromophoric species, starting from the readily accessible *E*‐isomers (Scheme 1).

## Results and Discussion

**Scheme 2:**
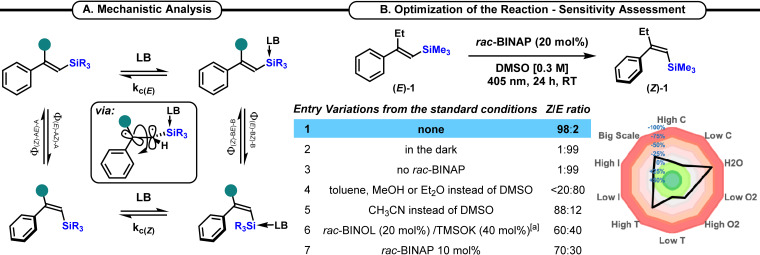
A. Mechanistic analysis. B. Optimization of the reaction – sensitivity assessment.

Thus, we conjectured that a possible interaction of the silicon center upon coordination with Lewis base catalyst might generate a photoactive species by changing the HOMO and LUMO level of the alkenyl silane. To promote this *n*→σ* interaction, we surmised that a phosphine catalyst would be an adequate catalyst, since the phosphine‐silicon interaction has been already documented.^[11]^ This blueprint has further prerequisites to be productive. First, the *E*→*Z* quantum yield (Φ_*E*→*Z*_) should be significantly enhanced upon coordination of the catalyst to the silicon species and the *Z*→*E* quantum yield (Φ_*Z*→*E*_) of both coordinated and non‐coordinated species should be substantially lower, ideally null, to circumvent the reversibility of the process (Scheme 2A).

We began our investigations with identification of the optimal reaction conditions to permit the *E*→*Z* interconversion of alkenyl silane **(*E*)‐1** (Scheme 2B). After an extensive set of optimization, we delineated the optimal reaction conditions for the isomerization of **(*E*)‐1** into **(*Z*)‐1**. Pleasingly, the use of 20 mol% of *rac*‐BINAP,^[12]^ as the catalyst, in DMSO at r.t. under irradiation at 405 nm for 24 h allowed the formation of **(*Z*)‐1** in a quantitative yield with a *Z*/*E* ratio of 98 : 2 (Scheme 2B, entry 1). Control experiments revealed that no isomerization occurred when the reaction was conducted in the dark, nor in the absence of *rac*‐BINAP under irradiation at 405 nm for 24 h (entries 2 & 3). DMSO was the best solvent, although CH_3_CN gave a promising 88 : 12 *Z*/*E* ratio (entries 4 & 5). In the course of our investigations, other Lewis base were tested to permit the *n*→σ* interaction with the silyl derivatives. Phenoxides, which are known to interact with silyl species, as demonstrated by Mukaiyama and others,^[13]^ were tested. However, despite an intensive screening, the *rac*‐BINOL/TMSOK combination remained less efficient than *rac*‐BINAP (entry 6).^[14]^ Finally, despite our efforts to decrease the catalyst loading, 20 mol% of *rac*‐BINAP was required to ensure the complete *E*→*Z* isomerization of silane **1** (entry 7). Then, to showcase the robustness of this isomerization protocol an assessment of the sensitivity of the reaction to the different parameters was carried out (Scheme 2B).^[15]^ The reaction showcased a fairly decent robustness with regard to the presence of oxygen, concentration and light intensity. However, a significant decrease of the *Z*/*E* ratio was witnessed in presence of water or when the reaction was conducted at higher temperature.

**Scheme 3:**
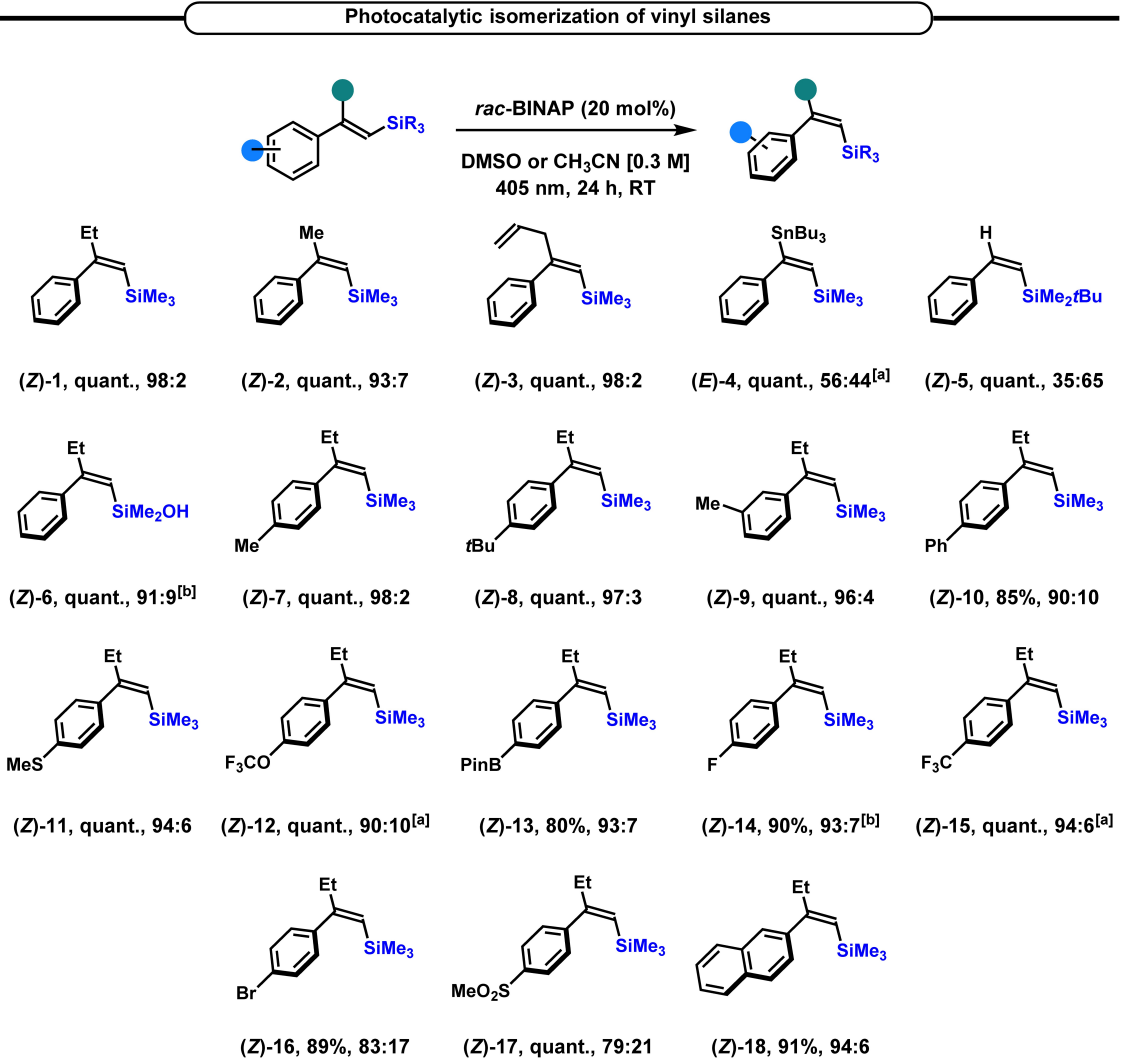
*E*→*Z* Isomerization of β‐aryl vinyl silanes, scope of the reaction, *Z : E* ratio were determined by GC‐FID.^[a]^ The reaction was conducted in CH_3_CN.^[b]^ *E : Z* ratio was determined by ^1^H NMR.

Then, with the optimized conditions in hand, we evaluated the applicability of this photoisomerization reaction to other vinyl silanes (Scheme 3). First, we evaluated the impact of the nature of the α‐substituent of the β‐(trimethylsilyl)styrene. The replacement of the ethyl residue by a methyl or an allyl substituent did not affect the efficiency of the reaction and **(*Z*)‐2** and **(*Z*)‐3** were isolated in excellent yields with an excellent *Z* : *E* ratio (93 : 7 and 98 : 2, respectively). However, the introduction of a *n*Bu_3_Sn substituent affected the isomerization, giving **(*E*)‐4** in a poor 56 : 44 *E* : *Z* ratio and the non‐substituted β‐(*tert*‐butyldimethylsilyl)styrene **(*Z*)‐5** was obtained with a modest 35 : 65 ratio, highlighting the low efficiency of this protocol on β‐substituted vinylsilanes. Then, we demonstrated the compatibility of our protocol with the synthetically useful silanol derivatives, a key building block in the Hiyama‐Denmark cross‐coupling reaction,^[16]^ which was readily isomerized, affording **(*Z*)‐6** with a pleasant 91 : 9 *Z* : *E* ratio. The substitution pattern on the aromatic ring was also evaluated. The presence of a methyl or *tert*‐butyl group at the *para*‐position did not alter the efficiency of the process, since **(*Z*)‐7** and **(*Z*)‐8** were obtained with excellent *Z : E* ratio, while **(*Z*)‐9** with the methyl group at the *meta*‐position gave a slightly lower *Z : E* ratio (96 : 4). Then, other substituents were introduced on the aromatic ring. The presence of a phenyl ring, a thiomethyl, a trifluoromethoxy, as well as the synthetically useful BPin group, at the *para*‐position of the aromatic ring of the vinylsilane was well tolerated, and the corresponding *Z*‐vinyl silanes (**(*Z*)‐10‐13**) were obtained in excellent to quantitative yields and excellent *Z : E* ratio under the standard reaction conditions. Then, the influence of halogens and electro‐withdrawing substituents at the *para*‐position of the aromatic ring was assessed. The fluoro and trifluoromethyl derivatives **(*Z*)‐14** and **(*Z*)‐15** were isolated in excellent yield and very good *Z : E* ratio, while the bromide and sulfone derivatives **(*Z*)‐16** and (***Z*** **)‐17** were less efficient in this isomerization reaction affording lower *Z : E* ratio.

**Scheme 4:**
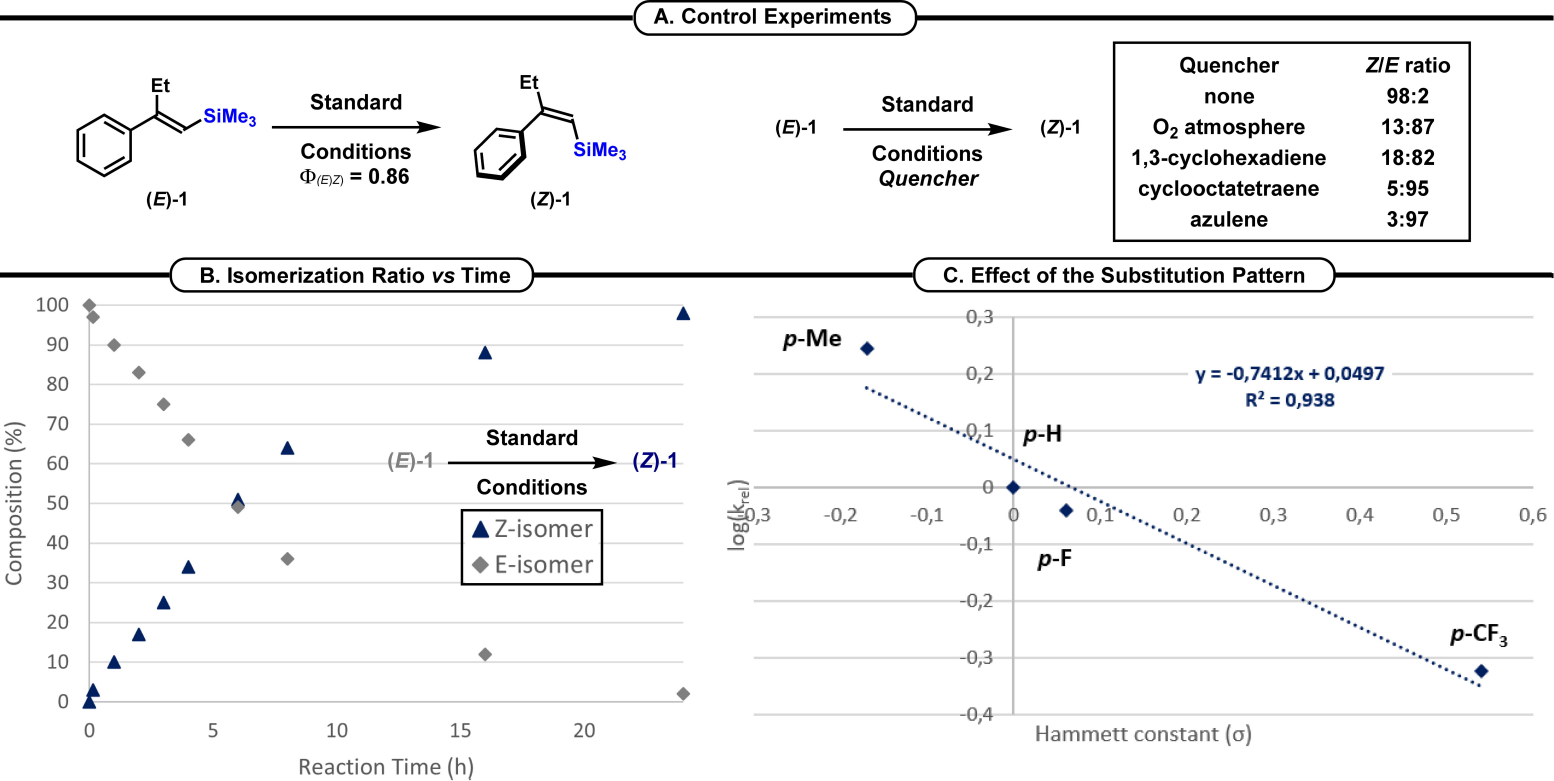
Study of the reaction mechanism. A. Control experiments. B. Isomerization ratio vs. Time. C. Effect of the substitution pattern.

Finally, the 2‐napthyl derivative was readily converted into the *Z*‐isomer **(*Z*)‐18** in 91 % yield and a pleasant 94 : 6 *Z : E* ratio. Then, some control experiments were carried out to intend to delineate a possible mechanism for this *E*→*Z* isomerization reaction (Scheme 4). First, a quantum yield of 0.86 was measured for the *E*→*Z* isomerization of **(*E*)‐1**, while the *Z*→*E* isomerization of **(*Z*)‐1** was unproductive (Φ_*Z*→*E*_=0). These measurements clearly highlight the non‐reactivity of the *Z*‐isomer in our reaction conditions (Scheme 4A). Then, we evaluated the impact of triplet and singlet state quenchers on the efficiency of this isomerization reaction (Scheme 4A). First, the reaction was conducted under an O_2_ atmosphere and the isomerization was inefficient. Likewise, in the presence of 1,3‐cyclohexadiene and cyclooctatetraene the *E*→*Z* isomerization was inhibited. Finally, the reaction conducted in the presence of azulene (a singlet and triplet quencher) was also inefficient. Hence, these results suggested the possible involvement of a triplet state mechanism, in contrast to our previous reports.^[6]^ To showcase the possible interaction of the *rac*‐BINAP catalyst with the vinyl silane, we conducted UV/Visible measurement. Unfortunately, we have not been able to witness a significant modification of the absorption of **(*E*)‐1** in the presence of *rac*‐BINAP, apart a slight enhancement of its absorption in the presence of the catalyst.^[17]^ Similarly, ^31^P NMR measurement did not evidence an interaction between the *rac*‐BINAP and **(*E*)‐1**. To further understand the reaction mechanism, a kinetic profile of the reaction was delineated. It confirmed that the photostationnary state was reached after 24 h (Scheme 4B). The impact of the substitution on the aryl residue of the β,β‐disubstituted vinyl silane was also studied. The presence of electron‐donating group at the *para*‐position of the aromatic ring increased the rate of the isomerization reaction, while the presence of electron‐withdrawing groups slows down the reaction.^[17]^ A correlation of the Hammett constant (σ) with these data unambiguously showcased these observations (*ρ*=−0.741, r^2^=0.938, Scheme 4C).

The observed negative slope suggests the development of a positive charge in the transition state, probably during the formation of the transient chromophoric species. One explanation might result from the Gutmann analysis of the Lewis base−Lewis acid interaction.^[18]^ Indeed, the interaction of the phosphine with the silicon atom would decrease the electron density on the silicon atom. The latter would then be stabilized by the presence of electron‐donating groups at the *para*‐position of the aromatic residue through conjugation, overall favoring the formation of the transient chromophoric species.

## Conclusion

In summary, we reported herein a novel reaction manifold to address the *E*→*Z* isomerization of β,β‐disubstituted vinyl silanes using *rac*‐BINAP as the catalyst under light irradiation (405 nm). The reaction proceeded smoothly and the *E*‐isomers were readily converted into the *Z*‐ones with excellent *E : Z* ratio (up to 98 : 1) and excellent yields. In addition, the reaction was scalable and a robustness evaluation of the transformation was carried out. The mechanism of the reaction was studied, supporting a possible triplet state mechanism. We believe that this novel protocol for the photocatalytic *E*→*Z contra*‐thermodynamic isomerization of β,β‐disubstituted vinyl silanes will offer a complementary approach to the community to access these difficult‐to‐synthesize substrates.

## Conflict of interest

The authors declare no conflict of interest.

1

## Supporting information

As a service to our authors and readers, this journal provides supporting information supplied by the authors. Such materials are peer reviewed and may be re‐organized for online delivery, but are not copy‐edited or typeset. Technical support issues arising from supporting information (other than missing files) should be addressed to the authors.

Supporting Information

## Data Availability

Research data are not shared.
