# Supplementary material for: Photocatalytic E→Z Contra‐Thermodynamic Isomerization of Vinyl Silanes with Lewis Base
Source: Chemistry. 2022 Jul 22;28(52):e202201514. doi: 10.1002/chem.202201514 (PMC9541780; doi:10.1002/chem.202201514)
Supplement: Supplementary file 1 — Supporting Information [file CHEM-28-0-s001.pdf]

Chemistry–A European Journal

Supporting Information

Photocatalytic *E*→*Z Contra*-Thermodynamic Isomerization of Vinyl Silanes with Lewis Base

Thi Minh Thi Le, Thibaud Brégent, Philippe Jubault, and Thomas Poisson*

1. General Information

All reactions were carried out using oven dried glassware and magnetic stirring under an atmosphere of argon unless otherwise stated. Vinyl silanes were isolated using flash chromatography, which was performed with silica gel (40-63 µm) or silica gel (15-40 µm) or by reversed-phase chromatography (RPC) using a 35 g Puriflash C18-HP 15 µm Interchim© cartridge (H_2O/CH_3CN gradient 90:10 to 0:100, rate: 3% CH_3CN per minutes).

Analytical thin layer chromatography was performed on silica gel aluminum plates with F-254 indicator and visualized by UV light (254 nm) and/or chemical staining with a $KMnO_4$ solution. All reagents were purchased from commercial suppliers (Sigma Aldrich, Fisher Scientific, TCI, VWR and Fluorochem) and used as received except the following: THF and toluene were distilled over sodium and benzophenone under an argon atmosphere, DCM over CaH_2 under argon. Technical grade solvents for extraction and purification (cyclohexane, dichloromethane (DCM), n-pentane, ethyl acetate (EtOAc), diethyl ether (Et_2O), petroleum ether (PE) and ethanol) were used without purification.

^{1}H NMR spectra were recorded on a Brucker DXP 300 instrument at 300 MHz (75 MHz for ^{13}C, 282 MHz for ^{19}F) in $CDCl_3$ at room temperature unless otherwise stated. Chemical shifts (δ) were quoted in parts per million (ppm) relative to the residual peak of $CDCl_3$ (δ_H = 7.267 ppm and δ_C = 77.16 ppm; or relative to external $CFCl_3$: δ = 0.00 ppm). Spectra are reported as follows: chemical shift δ (ppm), multiplicity (s = singlet, d = doublet, dd = doublet of doublets, dt = doublet of triplets, t = triplet, td = triplet of doublets, q = quartet, qd = quartet of doublets, p = quintuplet, m = multiplet), integration and coupling constant.

High-resolution mass spectra (HRMS) were recorded on Waters LCT Premier spectrometer. IR spectra were recorded on a PerkinElmer Spectrum 100. GC-FID analysis was obtained on a SCION 436-GC apparatus equipped with a RXI 5MS RESTEK column (internal diameter 30 x 0.25 mm, 0.25 µm deposit). Absorption spectra were recorded on UV-Visible Agilent Cary 60 spectrophotometer.

The lamp used for the isomerization reaction was an EVOLUCHEM 18W with λ_{max} = 405 nm. Reactions were setting up with this lamp respecting a distance d = 5 cm between the lamp and the reaction tube.

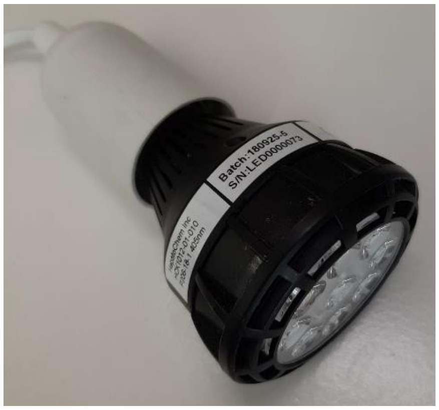

Photo 1: Evoluchem 18W 405 nm

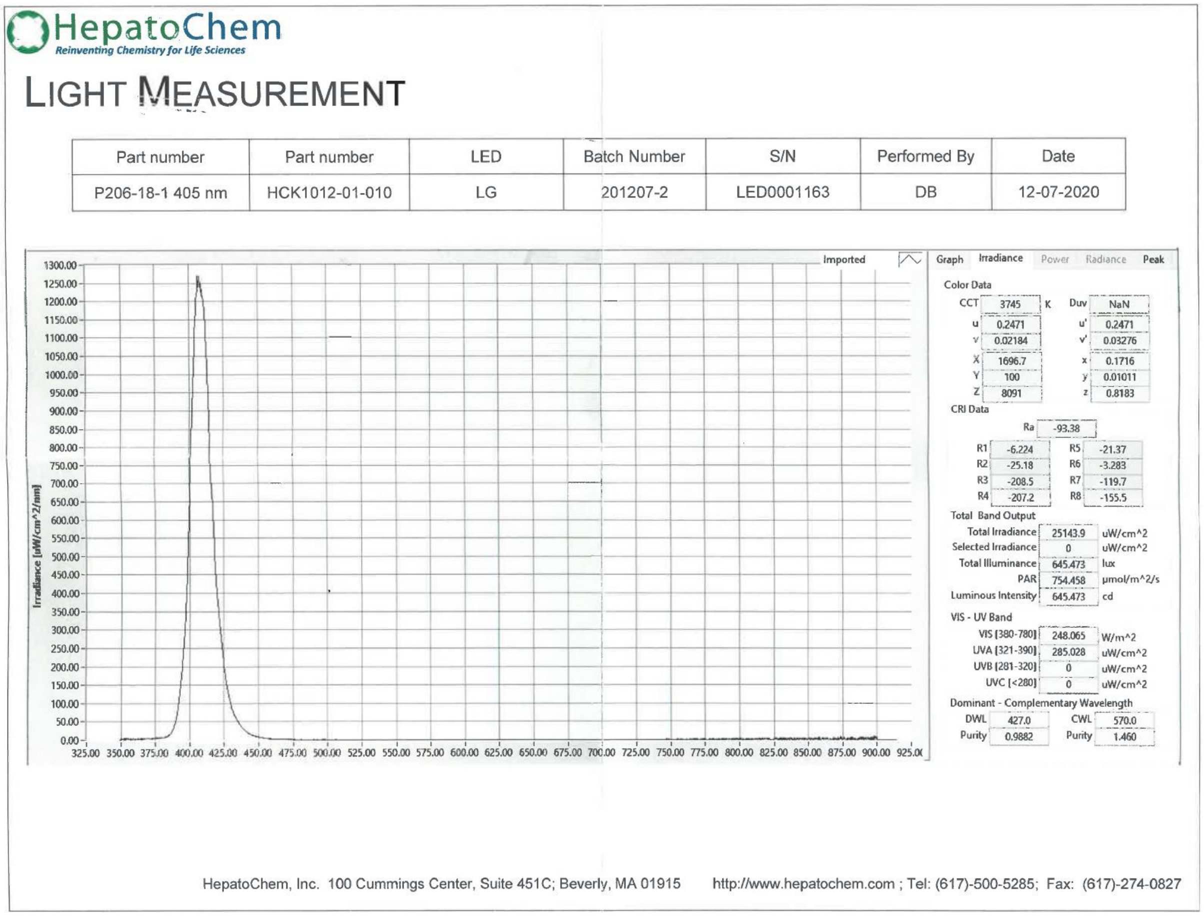
HepatoChem
Reinventing Chemistry for Life Sciences

LIGHT MEASUREMENT

Part number	Part number	LED	Batch Number	S/N	Performed By	Date
P206-18-1 405 nm	HCK1012-01-010	LG	201207-2	LED0001163	DB	12-07-2020

HepatoChem, Inc. 100 Cummings Center, Suite 451C; Beverly, MA 01915 http://www.hepatochem.com ; Tel: (617)-500-5285; Fax: (617)-274-0827

Emission spectrum of Evoluchem 18W 405 nm

2. Synthesis of starting materials

General Procedure A for the synthesis of vinyl silanes (*E*)-1 and (*E*)-7 to (*E*)-15[1]

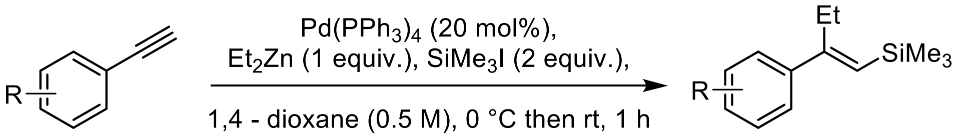

In an oven-dried flask were added $Pd(PPh_3)_4$ (0.02 equiv.), the corresponding phenylacetylene (1 equiv.) and dioxane (0.5 M) under an argon atmosphere. At 0 °C were added slowly diethylzinc (1 M in hexane, 1 equiv.), followed by iodotrimethylsilane (2 equiv.) and the reaction mixture was stirred for 1 h at room temperature. The reaction mixture was quenched with water, the aqueous phase was extracted with cyclohexane (3 times). The combined organic phases were dried over $MgSO_4$, the crude product was concentrated in vacuo and purified by flash chromatography with silica gel (40-63 µm or 15-40 µm) (PE:EtOAc: from 100:0 to 95:5) or by reversed-phase chromatography (RPC) using a 35 g Puriflash C18-HP 15 µm Interchim© cartridge (H_2O/CH_3CN gradient 90:10 to 0:100, rate: 3% CH_3CN per minutes).

[1] S. I. Faßbender, J. J. Molloy, C. Mück-Lichtenfeld, R. Gilmour, *Angew. Chem. Int. Ed.* **2019**, *58*, 18619–18626.

Characterization of (*E*)-1 and (*E*)-7 to (*E*)-15

(*E*)-trimethyl(2-phenylbut-1-en-1-yl)silane ((*E*)-1)

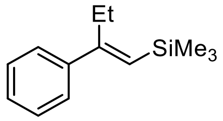

(*E*)-1

Obtained as a colorless oil (664 mg, 65% yield) after column chromatography on silica gel (PE:EtOAc from 100:0 to 95:5). ^{1}H NMR (300 MHz, $CDCl_3$) δ 7.41-7.31 (m, 2H), 7.29 – 7.16 (m, 3H), 5.69 (s, 1H), 2.59 (q, *J* = 7.5 Hz, 2H), 0.93 (t, *J* = 7.5 Hz, 3H), 0.14 (s, 9H). ^{13}C NMR (75 MHz, $CDCl_3$) δ 159.2, 143.5, 128.3, 127.4, 127.3, 126.4, 28.0, 14.3, 0.4. IR (ATR): ν_{max} = 3059, 2956, 2897, 2873, 1594, 1464, 1247, 852, 833, 758, 692 cm^{-1}. HRMS (EI+): m/z Calculated for $C_{13}H_{20}Si$ $[M]^+$: 204.1334, found 204.1336. These data were consistent with those reported in the literature. [1, 2]

(*E*)-trimethyl(2-(p-tolyl)but-1-en-1-yl)silane ((*E*)-7)

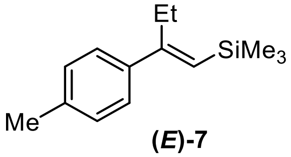

(*E*)-7

Obtained as a colorless oil (190 mg, 29% yield) after column chromatography on silica gel (PE 100%) and then by reversed-phase chromatography (RPC) using a 35 g Puriflash C18-HP 15 µm Interchim© cartridge (H_2O/CH_3CN gradient 90:10 to 0:100, rate: 3% CH_3CN per minutes). ^{1}H NMR (300 MHz, $CDCl_3$) δ 7.38 – 7.30 (m, 2H), 7.14 (d, *J* = 7.5 Hz, 2H), 5.74 (d, *J* = 2.3 Hz, 1H), 2.65 (qd, *J* = 7.5, 2.1 Hz, 2H), 2.36 (s, 3H), 1.01 (td, *J* = 7.5, 2.2 Hz, 3H), 0.21 (d, *J* = 2.3 Hz, 9H). ^{13}C NMR (75 MHz, $CDCl_3$) δ 159.0, 140.5, 137.0, 129.0, 126.4, 126.2, 28.0, 21.2, 14.3, 0.4. IR (ATR): ν_{max} = 3025, 2957, 2879, 1597, 1510, 1247, 923, 856, 831, 808, 766, 689, 496 cm^{-1}. HRMS (EI+): m/z Calculated for $C_{14}H_{22}Si$ $[M]^+$: 218.1491, found 218.1485.

(*E*)-(2-(4-(tert-butyl)phenyl)but-1-en-1-yl)trimethylsilane ((*E*)-8)

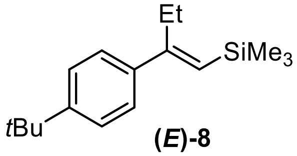

(*E*)-8

Obtained as a yellow oil (170 mg, 22% yield) after column chromatography on silica gel (PE:EtOAc from 100:0 to 95:5) and then by reversed-phase chromatography (RPC) using a 35 g Puriflash C18-HP 15 µm Interchim© cartridge (H_2O/CH_3CN gradient 90:10 to 0:100, rate: 3% CH_3CN per minutes). ^{1}H NMR (300 MHz, $CDCl_3$) δ 7.42 – 7.30 (m, 4H), 5.74 (s, 1H), 2.63 (q, *J* = 7.4 Hz, 2H), 1.32 (s, 9H), 1.00 (t, *J* = 7.5 Hz, 3H), 0.18 (s, 9H). ^{13}C NMR (75 MHz, $CDCl_3$) δ 158.79, 150.32, 140.30, 126.50, 125.91, 125.18, 34.60, 31.49, 27.88, 14.44, 0.41. IR (ATR): ν_{max} = 3084, 2961, 2902, 2872, 1595, 1465, 1363, 1247, 925, 858, 833, 752, 689, 549 cm^{-1}. HRMS (CI+): m/z Calculated for $C_{17}H_{29}Si$ $[M+H]^+$: 261.2039, found 261.2030.

(*E*)-trimethyl(2-(m-tolyl)but-1-en-1-yl)silane ((*E*)-9)

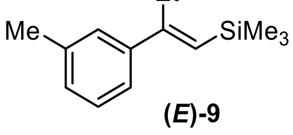

(*E*)-9

Obtained as a colorless oil (116 mg, 45% yield), after column chromatography on silica gel (PE:EtOAc from 100:0 to 95:5). ^{1}H NMR (300 MHz, $CDCl_3$) δ 7.23 – 7.15 (m, 3H), 7.11 – 7.04 (m, 1H), 5.72 (s, 1H), 2.64 (q, *J* = 7.5 Hz, 2H), 2.37 (s, 3H), 0.99 (t, *J* = 7.5 Hz, 3H), 0.20 (s, 9H). ^{13}C NMR (75 MHz, $CDCl_3$) δ 159.4, 143.5, 137.8, 128.2, 128.1, 127.2, 127.1, 123.4, 28.1, 21.7, 14.3, 0.4. IR (ATR):

[2] S. S. P. Chou, H. L. Kuo, C. J. Wang, C. Y. Tsai, C. M. Sun, *J. Org. Chem.* **1989**, *54*, 868-872.

ν_{max} = 3031, 2956, 2873, 1595, 1578, 1465, 1247, 868, 834, 783, 765, 689 cm^{-1}. HRMS (CI+): m/z Calculated for $C_{14}H_{23}Si$ [M+H]$^+$: 219.1569, found 219.1570.

(*E*)-(2-([1,1'-biphenyl]-4-yl)but-1-en-1-yl)trimethylsilane ((*E*)-10)

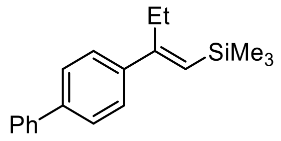

(*E*)-10

Obtained as a yellow oil (774 mg, 92% yield) after column chromatography on silica gel (PE:EtOAc from 100:0 to 90:10). ^{1}H NMR (300 MHz, $CDCl_3$) δ 7.66 – 7.54 (m, 4H), 7.55 – 7.40 (m, 4H), 7.41 – 7.30 (m, 1H), 5.84 (s, 1H), 2.70 (q, *J* = 7.5 Hz, 2H), 1.05 (t, *J* = 7.5 Hz, 3H), 0.23 (s, 9H). ^{13}C NMR (75 MHz, $CDCl_3$) δ 158.6, 142.3, 141.0, 140.1, 128.9, 127.5, 127.4, 127.1, 127.0, 126.8, 27.9, 14.4, 0.4. IR (ATR): ν_{max} = 3030, 2955, 2927, 2873, 1592, 1486, 1247, 1007, 925, 858, 834, 762, 732, 694 cm^{-1}. HRMS (EI+): m/z Calculated for $C_{19}H_{24}Si$ [M]$^+$: 280.1647, found 280.1645.

(E)-trimethyl(2-(4-(methylthio)phenyl)but-1-en-1-yl)silane ((*E*)-11)

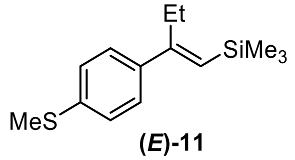

(*E*)-11

Obtained as a yellow oil (117 mg, 16% yield) after column chromatography on silica gel (PE:EtOAc from 100:0 to 95:5) and then by reversed-phase chromatography (RPC) using a 35 g Puriflash C18-HP 15 µm Interchim© cartridge (H_2O/CH_3CN gradient 90:10 to 0:100, rate: 3% CH_3CN per minutes). ^{1}H NMR (300 MHz, $CDCl_3$) δ 7.54 – 7.40 (m, 2H), 7.38 – 7.25 (m, 2H), 5.85 (s, 1H), 2.73 (q, *J* = 7.5 Hz, 2H), 2.58 (s, 3H), 1.09 (t, *J* = 7.5 Hz, 3H), 0.30 (s, 9H). ^{13}C NMR (75 MHz, $CDCl_3$) δ 158.3, 140.1, 137.3, 126.8, 126.8, 126.5, 27.8, 16.0, 14.3, 0.4. IR (ATR): ν_{max} = 3072, 2955, 2871, 1591, 1492, 1246, 922, 810, 831, 810, 760, 689, 500 cm^{-1}.

(E)-trimethyl(2-(4-(trifluoromethoxy)phenyl)but-1-en-1-yl)silane ((*E*)-12)

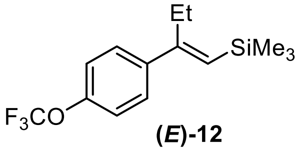

(*E*)-12

Obtained as a yellow oil (732 mg, 85% yield) after column chromatography on silica gel (PE:EtOAc from 100:0 to 95:5). ^{1}H NMR (300 MHz, $CDCl_3$) δ 7.42 (d, *J* = 8.7 Hz, 2H), 7.20 – 7.10 (m, 2H), 5.74 (s, 1H), 2.64 (q, *J* = 7.5 Hz, 2H), 0.99 (t, *J* = 7.5 Hz, 3H), 0.21 (s, 9H). ^{19}F NMR (282 MHz, $CDCl_3$) δ -58.39. ^{13}C NMR (75 MHz, $CDCl_3$) δ 157.8, 148.54 (q, *J* = 1.8 Hz), 142.3, 128.6, 127.7, 120.7 (q, *J* = 256.7 Hz), 120.7, 28.1, 14.1, 0.3. IR (ATR): ν_{max} = 2959, 2903, 1599, 1506, 1248, 1206, 1160, 1017, 921, 858, 835, 808, 751, 690 cm^{-1}. HRMS (EI+): m/z Calculated for $C_{14}H_{19}F_3OSi$ [M]$^+$:288.1157, found 288.1156.

(*E*)-trimethyl(2-(4-(4,4,5,5-tetramethyl-1,3,2-dioxaborolan-2-yl)phenyl)but-1-en-1-yl)silane ((*E*)-13)

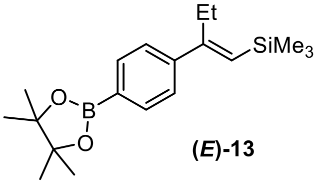

(*E*)-13

Obtained as a colorless sticky oil (340 mg, 46% yield) after column chromatography on silica gel (PE:EtOAc from 100:0 to 95:5). ^{1}H NMR (300 MHz, $CDCl_3$) δ 7.81 – 7.70 (m, 2H), 7.44 – 7.33 (m, 2H), 5.77 (s, 1H), 2.64 (q, *J* = 7.5 Hz, 2H), 1.35 (s, 12H), 0.96 (t, *J* = 7.5 Hz, 3H), 0.19 (s, 9H). ^{13}C NMR (75 MHz, $CDCl_3$) δ 159.2, 146.3, 134.8, 128.2, 125.7, 83.9, 27.9, 25.0, 14.2, 0.4. The carbon bearing boron was not observed. IR (ATR): ν_{max} = 3080, 2959, 1608, 1593, 1397, 1358, 1142, 1083, 857, 835, 658 cm^{-1}. HRMS (AP+): m/z Calculated for $C_{19}H_{32}BO_2Si$ [M+H]$^+$: 331.2265, found 331.2272. These data were consistent with those reported in the literature. [1]

(*E*)-(2-(4-fluorophenyl)but-1-en-1-yl)trimethylsilane ((*E*)-14)

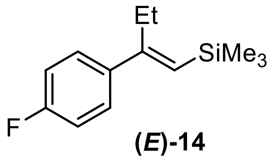

(*E*)-14

Obtained as a colorless oil (791 mg, 70% yield) after column chromatography on silica gel (PE:EtOAc from 100:0 to 95:5). ^{1}H NMR (300 MHz, $CDCl_3$) δ 7.48 – 7.29 (m, 2H), 7.11 – 6.89 (m, 2H), 5.70 (s, 1H), 2.62 (q, *J* = 7.5 Hz, 2H), 0.98 (t, *J* = 7.5 Hz, 3H), 0.20 (s, 9H). ^{19}F NMR (282 MHz, $CDCl_3$) δ -116.29. ^{13}C NMR (75 MHz, $CDCl_3$) δ 162.4 (d, *J* = 245.9 Hz), 158.2, 139.6 (d, *J* = 3.3 Hz), 128.1 (d, *J* = 7.7 Hz), 127.5 (d, *J* = 0.8 Hz), 115.1 (d, *J* = 21.2 Hz), 28.2, 14.3, 0.4. IR (ATR): ν_{max} = 2958, 2903, 2879, 1602, 1506, 1248, 1232, 1159, 923, 859, 832, 690, 511 cm^{-1}. HRMS (EI+): m/z Calculated for $C_{13}H_{19}FSi$ $[M]^{+}$: 222.1240, found 22.1238. These data were consistent with those reported in the literature. [1]

(*E*)-trimethyl(2-(4-(trifluoromethyl)phenyl)but-1-en-1-yl)silane ((*E*)-15)

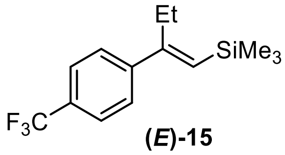

(*E*)-15

Obtained as a yellow oil (964 mg, 71% yield) after column chromatography on silica gel (PE:EtOAc from 100:0 to 95:5). ^{1}H NMR (300 MHz, $CDCl_3$) δ 7.29 (d, *J* = 8.3 Hz, 2H), 7.21 (d, *J* = 8.3 Hz, 2H), 5.53 (s, 1H), 2.38 (q, *J* = 7.5 Hz, 2H), 0.70 (t, *J* = 7.5 Hz, 3H), -0.06 (s, 9H). ^{19}F NMR (282 MHz, $CDCl_3$) δ -62.89. ^{13}C NMR (75 MHz, $CDCl_3$) δ 157.9, 147.2, 130.0, 129.3 (q, *J* = 32.5 Hz), 126.7, 125.3 (q, *J* = 3.7 Hz), 124.5 (q, *J* = 271.8 Hz), 28.0, 14.0, 0.2. IR (ATR): ν_{max} = 2959, 2903, 1615, 1598, 1407, 1322, 1249, 1164, 1124, 1067, 1015, 926, 858, 834, 690, 626 cm^{-1}. HRMS (EI+): m/z Calculated for $C_{14}H_{19}F_3Si$ $[M]^{+}$:272.1208, found 272.1212.

(*E*)-(2-(4-bromophenyl)but-1-en-1-yl)trimethylsilane ((*E*)-16)

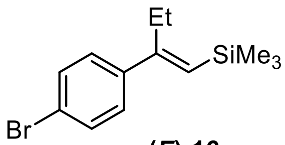

(*E*)-16

Obtained as a colorless oil (131 mg, 16% yield) after column chromatography on silica gel (PE:EtOAc from 100:0 to 95:5). ^{1}H NMR (300 MHz, $CDCl_3$) δ 7.42 – 7.33 (m, 2H), 7.28 – 7.12 (m, 2H), 5.68 (s, 1H), 2.55 (q, *J* = 7.5 Hz, 2H), 0.91 (t, *J* = 7.5 Hz, 3H), 0.14 (s, 9H). ^{13}C NMR (75 MHz, $CDCl_3$) δ 157.9, 142.4, 131.4, 128.3, 128.1, 121.2, 27.9, 14.1, 0.3. IR (ATR): ν_{max} = 2957, 2879, 1593, 1486, 1247, 1072, 1008, 923, 857, 831, 813, 690 cm^{-1}. HRMS (EI+): m/z Calculated for $C_{13}H_{19}BrSi$ $[M]^{+}$: 282.0439, found 282.0444. These data were consistent with those reported in the literature. [1]

(*E*)-trimethyl(2-(4-(methylsulfonyl)phenyl)but-1-en-1-yl)silane ((*E*)-17)

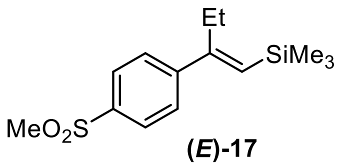

(*E*)-17

Obtained as a yellow oil (121 mg, 13% yield) after column chromatography on silica gel (PE:EtOAc from 100:0 to 90:10). ^{1}H NMR (300 MHz, $CDCl_3$) δ 7.59 (d, *J* = 8.5 Hz, 2H), 7.27 (d, *J* = 8.6 Hz, 2H), 5.55 (s, 1H), 2.78 (s, 3H), 2.37 (q, *J* = 7.5 Hz, 2H), 0.68 (t, *J* = 7.5 Hz, 3H), -0.08 (s, 9H). ^{13}C NMR (75 MHz, $CDCl_3$) δ 157.3, 149.2, 138.9, 131.5, 127.4, 127.3, 44.7, 27.8, 14.0, 0.2. IR (ATR): ν_{max} = 2962, 2877, 2257, 1592, 1310, 1248, 1148, 1095, 956, 924, 858, 836, 780, 729, 692, 611, 528 cm^{-1}. HRMS (CI+): m/z Calculated for $C_{14}H_{23}O_2SSi$ $[M+H]^{+}$: 283.1188, found 283.1190. These data were consistent with those reported in the literature. [1]

(*E*)-trimethyl(2-(naphthalen-2-yl)but-1-en-1-yl)silane ((*E*)-18)

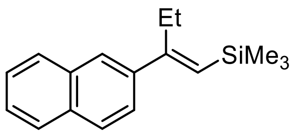

(*E*)-18

Obtained as a yellow oil (185 mg, 99% yield) after column chromatography on silica gel (PE:EtOAc from 100:0 to 90:10). ^{1}H NMR (300 MHz, $CDCl_3$) δ 7.91 – 7.73 (m, 4H), 7.61 – 7.56 (m, 1H), 7.52 – 7.38 (m, 2H), 5.90 (s, 1H), 2.77 (q, *J* = 7.5 Hz, 2H), 1.04 (t, *J* = 7.5 Hz, 3H), 0.24 (s, 9H). ^{13}C NMR (75 MHz, $CDCl_3$) δ 159.0, 140.7, 133.6, 132.9, 128.3, 128.1, 127.8, 127.6, 126.2, 125.8, 125.1, 124.9, 28.1, 14.4, 0.4. IR (ATR): ν_{max} = 3056, 2955, 2897, 2874, 1597, 1505, 1432, 1247, 925, 874, 836, 812, 743, 689, 474 cm^{-1}. HRMS (EI+): m/z Calculated for $C_{17}H_{22}Si$ $[M]^+$:254.1491, found 254.1497.

Synthesis and characterization of (*E*)-2, (*E*)-3, (*Z*)-4, (*E*)-5 and (*E*)-6

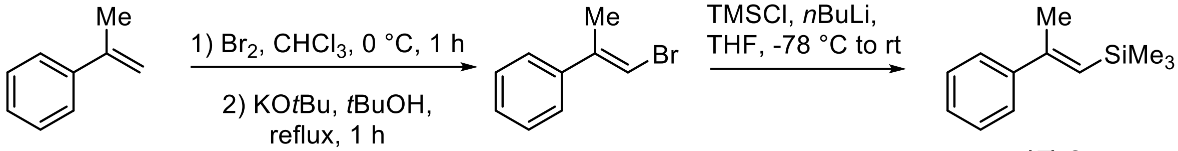

(*E*)-2

***(E)-2** was prepared according to the reported method.* [1]

(*E*)-trimethyl(2-phenylprop-1-en-1-yl)silane ((*E*)-2)

Obtained as a colorless oil (116 mg, 15% yield) after column chromatography on silica gel (PE:EtOAc from 100:0 to 90:10) and then by reversed-phase chromatography (RPC) using a 35 g Puriflash C18-HP 15 µm Interchim© cartridge (H_2O/CH_3CN gradient 90:10 to 0:100, rate: 3% CH_3CN per minutes). ^{1}H NMR (300 MHz, $CDCl_3$) δ 7.50 – 7.41 (m, 2H), 7.37 – 7.20 (m, 3H), 5.91 (q, *J* = 0.8 Hz, 1H), 2.21 (d, *J* = 0.7 Hz, 3H), 0.19 (s, 9H). ^{13}C NMR (75 MHz, $CDCl_3$) δ 151.8, 144.5, 128.2, 127.5, 127.4, 125.6, 21.1, 0.2. IR (ATR): ν_{max} = 3055, 2954, 2897, 2854, 1598, 1572, 1494, 1443, 1247, 849, 834, 756, 690 cm^{-1}. HRMS (CI+): m/z Calculated for $C_{12}H_{19}Si$ $[M+H]^+$:191.1256, found 191.1261. These data were consistent with those reported in the literature. [1]

(*E*)-trimethyl(2-phenylpenta-1,4-dien-1-yl)silane ((*E*)-3)

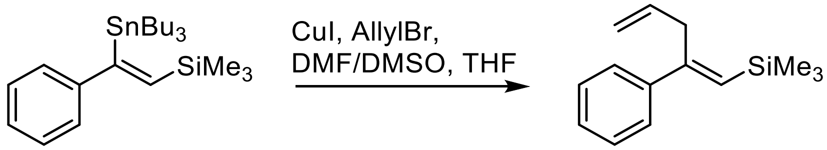

(*Z*)-4 **(*E*)-3**

(E)-3 was prepared according to the reported method. [3]

Obtained as a yellow oil (369 mg, 53% yield) after column chromatography on silica gel (PE:EtOAc from 8:2 to 7:3). ^{1}H NMR (300 MHz, $CDCl_3$) δ 7.39 – 7.23 (m, 2H), 7.23 – 7.00 (m, 3H), 5.79 (s, 1H), 5.64 (ddt, *J* = 16.4, 10.2, 6.0 Hz, 1H), 5.01 – 4.71 (m, 2H), 3.23 (dt, *J* = 6.1, 1.8 Hz, 2H), 0.04 (s, 9H). ^{13}C NMR (75 MHz, $CDCl_3$) δ 154.1, 143.5, 136.7, 129.6, 128.2, 127.4, 126.4, 116.2, 38.9, 0.4. IR (ATR): ν_{max} = 3080, 2954, 2895,

[3] F. Sasaki, T. Endo, M. Noguchi, K. Kawai and T. Nakano, *Appl. Organometal. Chem.* **2008**, *22*, 128–138.

1595, 1571, 1494, 1443, 1247, 991, 912, 851, 834, 759, 691 cm^{-1}. These data were consistent with those reported in the literature. [3]

(*Z*)-trimethyl(2-phenyl-2-(tributylstannyl)vinyl)silane (*Z*)-4

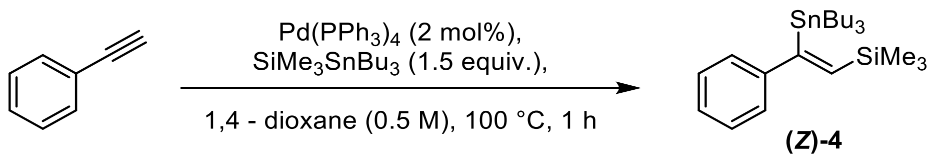

(*Z*)-4

***((Z)-4)** were prepared according to the reported method.* [1]

Obtained as a colorless oil (1.86 g, 80% yield) after column chromatography on silica gel (PE:EtOAc from 8:2 to 7:3). ^{1}H NMR (300 MHz, $CDCl_3$) δ 7.32 (t, *J* = 7.5 Hz, 2H), 7.19 (t, *J* = 7.3 Hz, 1H), 7.06 (d, *J* = 8.1 Hz, 2H), 6.64 (s, 1H), 1.56 – 1.41 (m, 6H), 1.41 – 1.27 (m, 6H), 1.02 – 0.82 (m, 15H), 0.27 (s, 9H). ^{13}C NMR (75 MHz, $CDCl_3$) δ 166.2, 152.1, 148.5, 128.1, 126.1, 125.6, 29.3, 27.6, 13.8, 12.2, 0.4. IR (ATR): ν_{max} = 3068, 2955, 2921, 2872, 2854, 1464, 1377, 1246, 961, 860, 850, 830, 754, 696, 408 cm^{-1}. These data were consistent with those reported in the literature. [1, 4]

Synthesis of (*E*)-tert-butyldimethyl(styryl)silane ((*E*)-5)

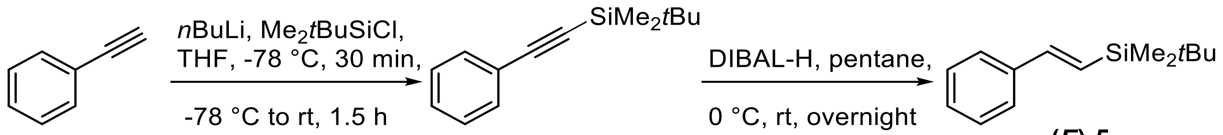

(*E*)-5

In an oven dried flask were added phenylacetylene (0.55 mL, 5.0 mmol, 1 equiv.) and dry THF (10 mL) under an argon atmosphere. The flask was cooled to -78 °C before the dropwise addition of *n*-butyllithium (2.44 M in hexane, 2.25 mL, 5.5 mmol, 1.1 equiv.) and the reaction mixture was stirred for 30 min. *tert*-Butyldimethylsilyl chloride (1.1 mL, 6.0 mmol, 1.2 equiv.) was added dropwise and the reaction mixture was stirred for 30 min at -78 °C. The reaction mixture was allowed to warm to room temperature and was stirred for 1.5 h. The reaction was quenched with an aq. sat. NH_4Cl solution and organics were separated. The aqueous phase was extracted with Et_2O (3 times) and the combined organic phases were washed with brine, dried over $MgSO_4$, and concentrated under reduced pressure. The crude residue was purified by column chromatography (SiO_2, 100% *n*-pentane) to afford the desired product as a clear oil (684 mg, 63%).

DIBAL-H (1.0 M in DCM, 3.42 mL, 4.11 mmol, 1.3 equiv.) was added dropwise to a solution of *tert*-Butyldimethyl(phenylethynyl)silane (684 mg, 3.16 mmol, 1 equiv.) in *n*-pentane (10 mL) at 0 °C under an argon atmosphere. The reaction mixture was stirred at room temperature overnight. The reaction was quenched with cold H_2SO_4 (5%, aq.) at 0 °C and filtered over celite. The reaction was diluted in Et_2O and organics separated. The aqueous phase was extracted with Et_2O (3 times). The combined organic phases were washed with brine (50 mL), dried over $MgSO_4$, and concentrated under reduced pressure. The crude residue was purified by column chromatography (SiO_2, 100% *n*-pentane) to afford the desired product as a colorless oil (459 mg, 67%). ^{1}H NMR (300 MHz, $CDCl_3$) δ 7.35 – 7.23 (m, 2H), 7.22 – 7.10 (m, 2H), 7.11 – 7.07 (m, 1H), 6.72 (d, *J* = 19.2 Hz, 1H), 6.31 (dd, *J* = 19.2, 0.7 Hz, 1H), 0.75 (d, *J* = 0.9 Hz, 9H), -0.05 (s, 6H). ^{13}C NMR (75 MHz, $CDCl_3$) δ 145.0, 138.6, 128.7, 128.1, 126.9, 126.5, 26.7, 16.9, -5.9. IR (ATR): ν_{max} = 3025,

[4] I. Hemeon, R. D. Singer, *J. Mol. Catal. A Chem.* **2004**, *214*, 33-44.

2953, 2927, 2883, 2856, 1605, 1574, 1470, 1249, 988, 828, 803, 774, 732, 688, 661 cm^{-1}. These data were consistent with those reported in the literature. [1]

(*E*)-dimethyl(2-phenylbut-1-en-1-yl)silanol ((*E*)-6)

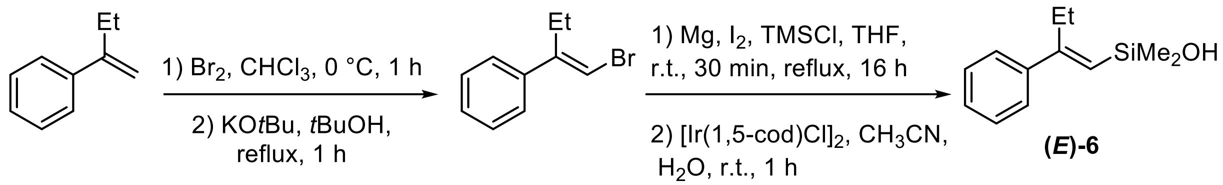

***(E)-6** were prepared according to the reported method [1]*

Obtained as a colorless oil (116 mg, 15% yield) after column chromatography on silica gel (PE:EtOAc from 100:0 to 90:10) and then by reversed-phase chromatography (RPC) using a 35 g Puriflash C18-HP 15 µm Interchim© cartridge (H_2O/CH_3CN gradient 90:10 to 0:100, rate: 3% CH_3CN per minutes). ^1H NMR (300 MHz, $CDCl_3$) δ 7.46 – 7.33 (m, 2H), 7.34 – 7.26 (m, 3H), 5.72 (s, 1H), 2.70 (q, *J* = 7.5 Hz, 2H), 0.98 (t, *J* = 7.5 Hz, 3H), 0.28 (s, 6H). ^{13}C NMR (75 MHz, $CDCl_3$) δ 159.6, 143.4, 128.3, 127.4, 127.1, 126.4, 28.0, 14.2, 2.6. IR (ATR): ν_{max} = 3058, 2962, 2874, 1595, 1571, 1494, 1443, 1251, 1030, 929, 837, 775, 693 cm^{-1}.

3. Optimization

Optimization with *rac*-BINOL - Wavelength Effect

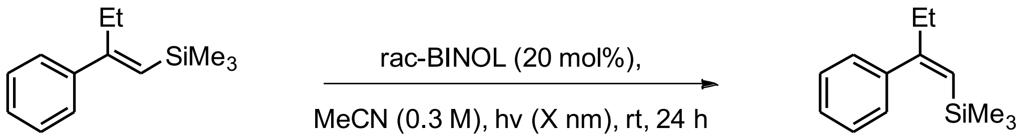

Entry	Wavelength (X)	Ratio E/Z [a]
1	400-500 nm	98:2
**2**	**405 nm**	**66:34**

Table 1: Study of the wavelength [a] E/Z Ratio determined by ^{1}H NMR.

Optimization with rac-BINOL in the presence of base

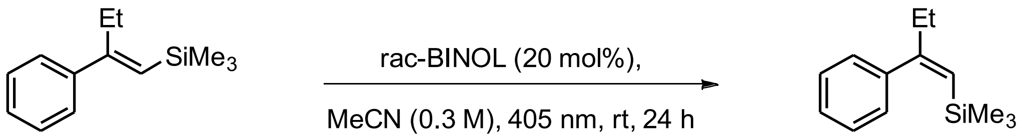

Entry	Base (20 mol%)	Ratio E/Z [a]
1	-	66:34
2	*t*BuOLi	84:16
3	$(Me_3Si)NLi$	79:21
4	NaH	56:44
**5**	**Me_3SiOK**	**35:65**
6[b]	Me_3SiOK	40:60
7[c]	Me_3SiOK	89:11

Table 2 : Study of base effect

[a] E/Z Ratio determined by ^{1}H NMR. [b] THF was used instead of MeCN. [c] DCM was used instead of MeCN.

Solvent Effect

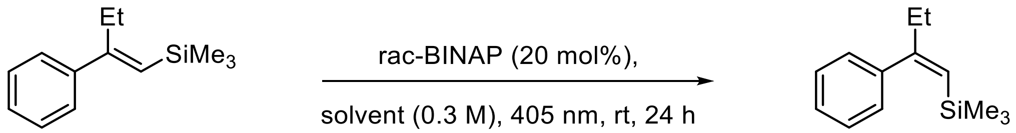

Entry	Solvent	Ratio E/Z
1	Toluene	89:11
2	Et_2O	83:17
3	THF	60:40
4	DMF	89:11
5	MeCN	12:88
6	DMSO	5:95
7	MeOH	94:6

Table 3 : Study of the solvent

Catalyst Loading and Concentration

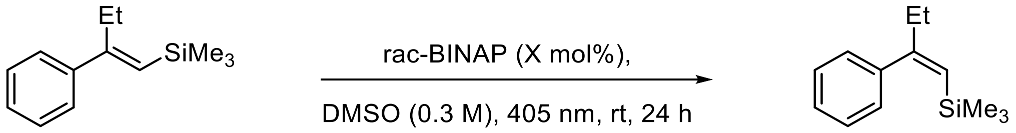

Entry	Catalyst Loading	Ratio E/Z
**1**	20 mol%	5 :95
**2**	10 mol%	30 :70
**3**	5 mol%	69 :31

Table 4 : Study of the catalyst loading

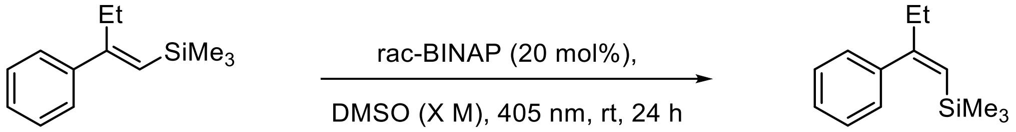

Entry	Concentration	Ratio E/Z
**1**	0.6 M	7:93
**2**	0.3 M	5:95
**3**	0.15 M	7:93

Table 5 : Study of the concentration

Control experiments

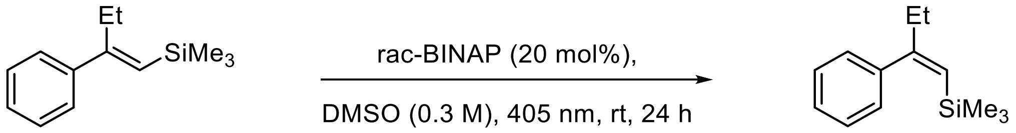

Entry	Deviation from the standard conditions	Ratio E/Z
**1**	-	5 :95
**2**	Dark	98 :2
**3**	Dark, no catalyst	98 :2
**4**	No catalyst	98 :2
**5**	50 °C	49 :51
**6**	Under air	16 :84

Table 6 : Control experiments

Reverse Z-> E process

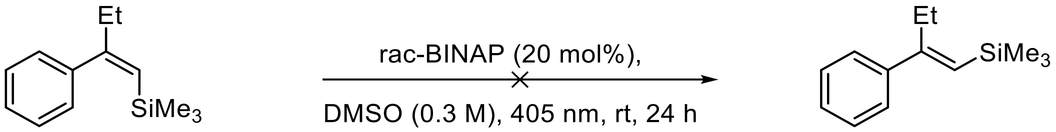

4. Sensitivity experiments

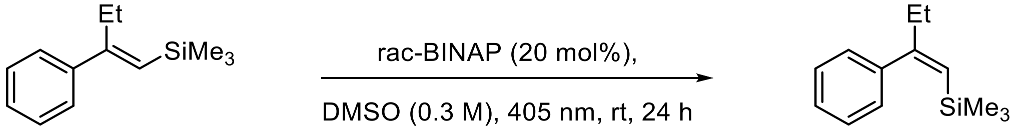

The sensitivity assessment was conducted as described in Pitzer, L.; Schäfers, F.; Glorius, F. *Angew. Chem. Int. Ed.* **2019**, *58*, 8572-8576.

The influence of water, oxygen, light intensity, temperature and concentration was investigated, as well as the scalability. While one parameter was varied, all other parameters were kept constant. The *Z*/*E* isomer ratio was determined by GC-FID analysis of the crude mixture. The respective deviation was calculated in reference to a control reaction (ratio *Z*/*E* = 95:5 on 0.15 mmol scale)

The results are shown in *Table 7* and the respective *Figure 1*. The reaction showed to be overall rather robust. The addition of water (10%) appeared to have a negative effect (- 16%).

In summary, to reproduce the reaction, working in anhydrous medium is important, while the reaction seems to be not sensitive towards temperature change, lower light intensity, oxygen and also works at larger scale (*Photo 3*).

Entry	Parameters	Standard conditions	Conditions studied	(Z)-1a	Deviation (%)
1	Concentration	0.3 M	High : 0.6 M	93%	-2
2			Low : 0.15 M	93%	-2
3	Medium	Anhydrous DMSO	DMSO/H_2O 9:1	30%	-68
4	Atmosphere	Under argon	Degassed DMSO	80%	-16
5			Under air	84%	-12
6	Temperature	35 °C	25 °C	97%	2
7			50 °C	51%	-46
8	Intensity (W)	d = 5 cm	High : d = 1 cm	89%	-6
9			Low : d = 6 cm	89%	-6
10	Scale	0.3 mmol/1ml	3 mmol/10 mL	64%	-33

Table 7 : Conditions for the sensitivity assessment

(a) The reaction was setting up using a fan.

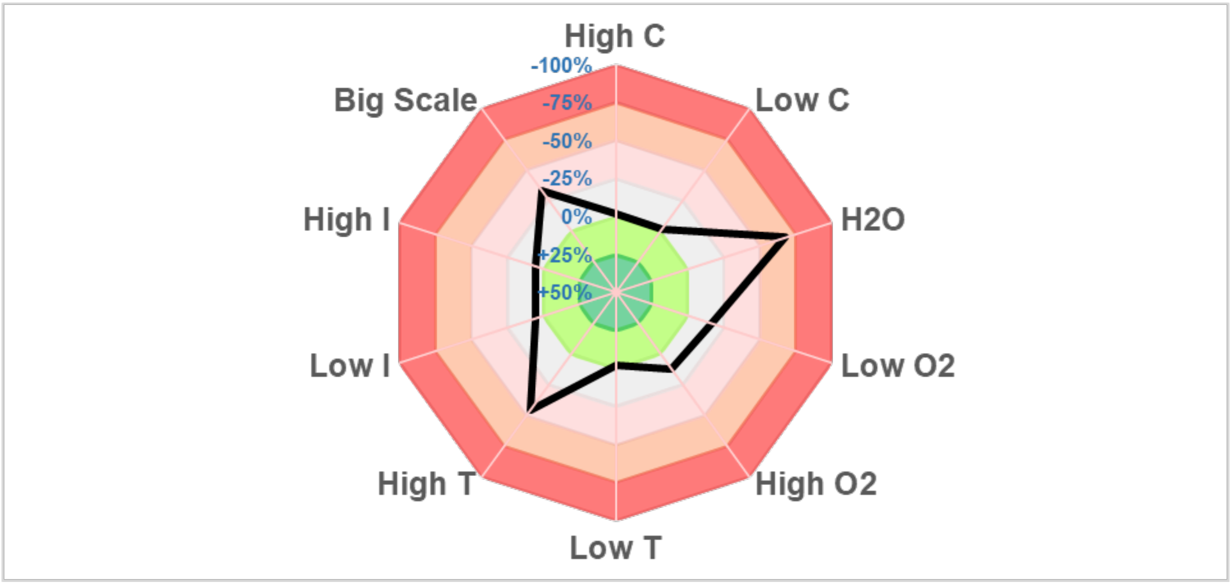

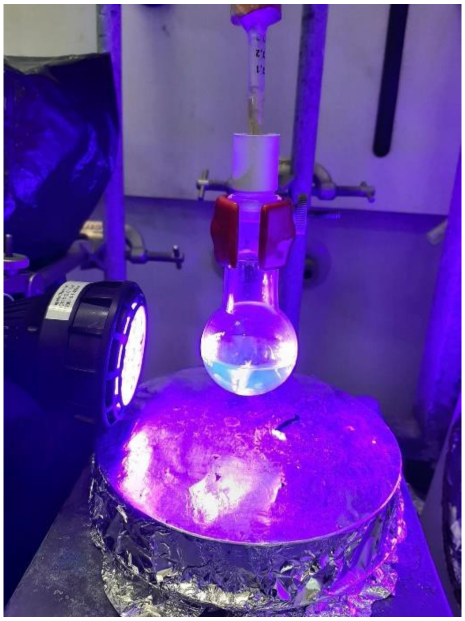

Photo 2: Isomerization on larger scale (1.5 mmol)

5. Mechanistic studies

UV/visible absorption measurements

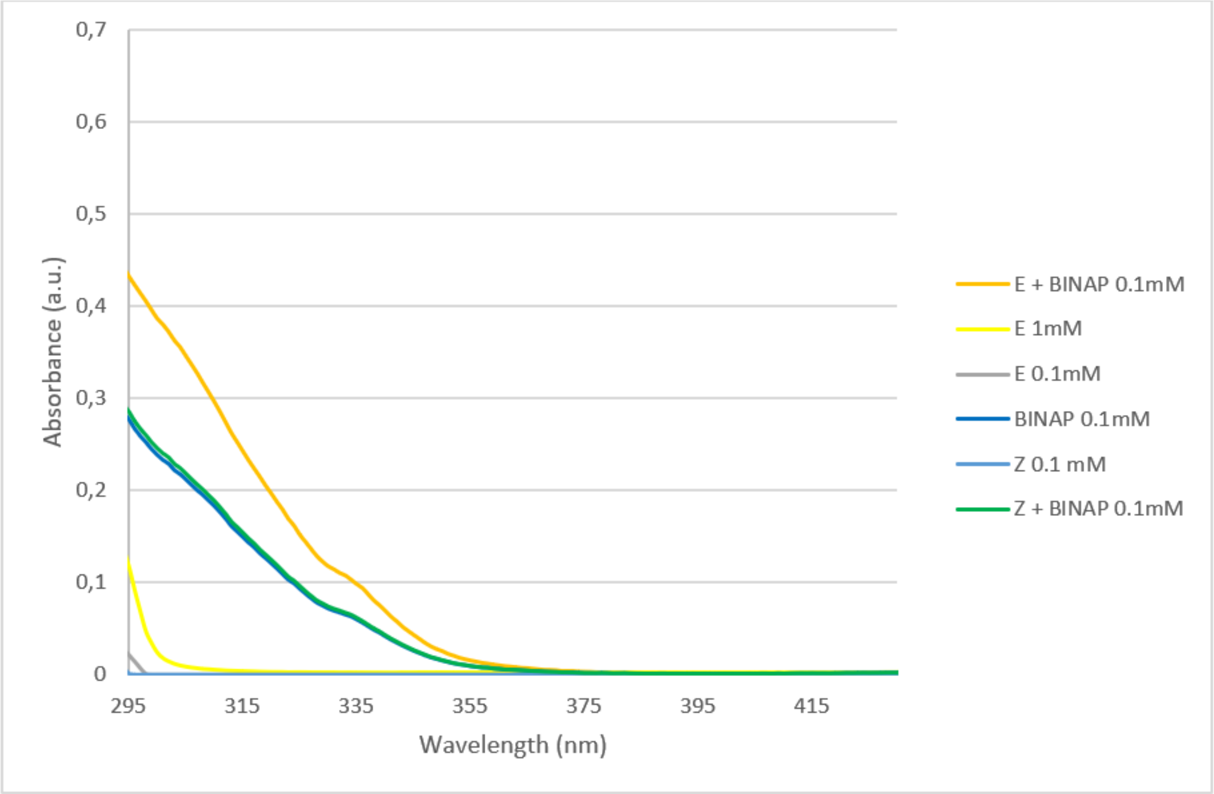

UV/visible absorption spectra at a concentration 0.1 mM in DMSO

Addition of triplet and singlet quenchers

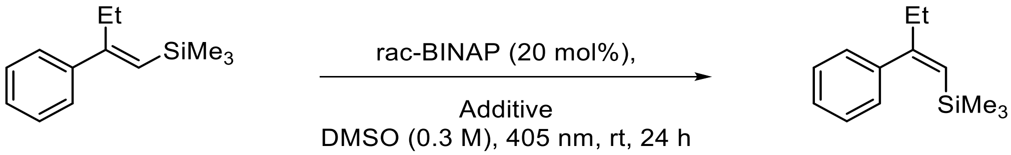

Additive	Ratio E:Z
none	2:98
O_2 atmosphere	87:13
1,3-cyclohexadiene (1 equiv.)	82:18
Cyclooctatetraene (1 equiv.)	95:5
Azulene (1 equiv.)	97:3

Table 8 : Experiments with triplet and singlet state quenchers

1,3-cyclohexadiene, O_2 and cyclooctatetraene are known to quench triplet states and azulene is known to quench both singlet and triplet states. With these experiments, we can suggest a triplet state mechanism.

Isomerization quantum yields measurements

Determination of the photon flux

the photon flux of lamp EVOLUCHEM 18W (λ_{max} = 405 nm) was determined by standard ferrioxalate actinometry. A solution of ferrioxalate (0.15 M) was prepared by dissolving potassium ferrioxalate hydrate (737 mg) in H_2SO_4 aq. solution (0.05 M, 10 mL). A buffered solution of 1,10-phenanthroline was prepared by dissolving 1,10-phenanthroline (20 mg) and sodium acetate (4.5 g) in H_2SO_4 aq. solution (0.5 M, 20 mL). Both solutions were stored in the dark.

To determine the photon flux of the lamp, the ferrioxalate solution (1000 µL) was placed in a microwave tube and irradiated for 90 s at λ_{max} = 405 nm. After irradiation, the phenanthroline solution (175 µL) was added and the mixture was stirred for 1h in the dark. The solution was transfered to a quartz cuvette and the absorption of the solution was measured at 510 nm. A non-irradiated sample was prepared and the absorption was measured. The average of the absorption of 3 experiments (irradiated and non-irradiated) was determined and used to calculate the conversion using *(eq. A)*.

$$n\,(Fe^{2+}) = \frac{V.\Delta A\,(510\,nm)}{l.\varepsilon} \qquad (eq. A)$$

In our case, *V* is the total volume (0.001175 L) of the solution after addition of buffered solution. *ΔA* is the difference in absorption at 510 nm between the irradiated and non-irradiated solutions, *l* is the path length (1.0 cm), and *ε* is the molar absorption coefficient of the ferrioxalate actinometer at 510 nm (11100 $L.cm^{-1}.mol^{-1}$). n (Fe^{2+}) was calculated to be 2.38 x 10^{-7} mol. The photon flux (ϕ_q) can be calculated using *(eq. B)*.

$$\varphi_q = \frac{n(Fe^{2+})}{\varphi_{Fer}.t.f} \qquad (eq. B)$$

Here, ϕ_{Fer} is the photoreaction quantum yield for the ferrioxalate actinometer (1.13 at λ_{ex} = 405 nm), t is the irradiation time (90 s), and f is the fraction of light absorbed at λ_{ex} = 405 nm by the ferrioxalate actinometer. This value is calculated using (*eq. C)* where A(405 nm) is the absorption of the ferrioxalate solution at 405 nm. An absorption spectrum gave an A(405 nm) value > 3, indicating that the fraction of absorbed light (f) is 0.999.

$$f = 1 - 10^{-A(405\,nm)} \qquad (eq. C)$$

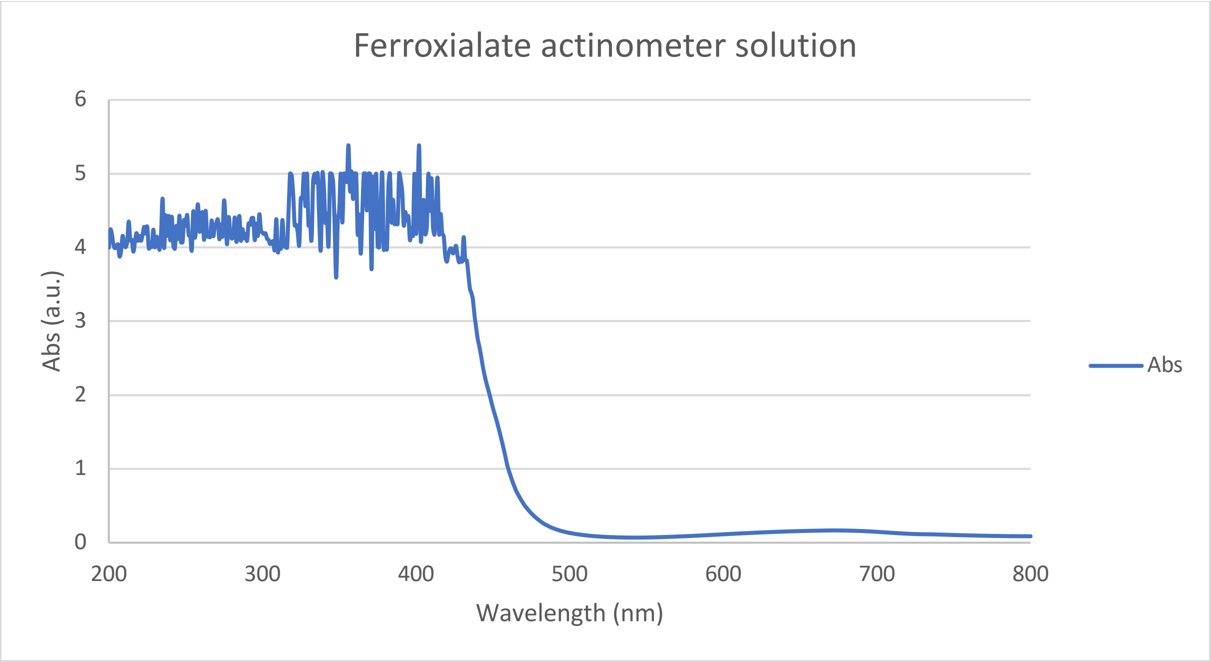

Figure 1: UV-visible absorption spectrum of ferrioxalate actinometer solution

Finally, the photon flux was determined to be **2.34 x 10^{-9} einstein.s^{-1}** (average from 3 parallel experiments).

Determination of isomerization quantum yield

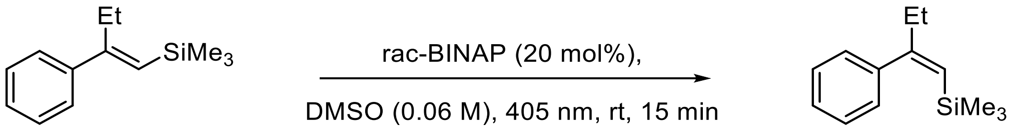

The quantum yield was calculated following general procedure **B** at 0.06 M for 15 min. After irradiation, the yield was determined by GC-FID analysis. The yield was determined to be 2.9 % (average from 3 parallel experiments) meaning 1.74 x 10^{-6} mol.

The quantum yield was determined using *(eq. D)* where the photon flux ϕ_q was 2.34 x 10^{-9} einstein.s^{-1}, *t* was the reaction time (900 s), and f_R the fraction of absorbed incident light (determined using *eq. C*). At 405 nm, the measured absorption was > 3 a.u. giving 0.999 for f_R.

$$\varphi = \frac{n\,(product)}{\varphi_q . t . f_R} \qquad (eq. D)$$

Finally, the reaction quantum yield $\phi_{E\rightarrow Z}$ was determined to be $\boldsymbol{\phi_{E\rightarrow Z}}$ **= 0.86**.

The same procedure was repeated starting from the (*Z*)-isomer to calculate the reaction quantum yield $\phi_{Z\rightarrow E}$.

After 3 experiments, the reaction quantum yield $\phi_{Z\rightarrow E}$ was determined to be $\boldsymbol{\phi_{Z\rightarrow E} = 0}$. (no conversion was observed).

Isomerization Ratio vs Time

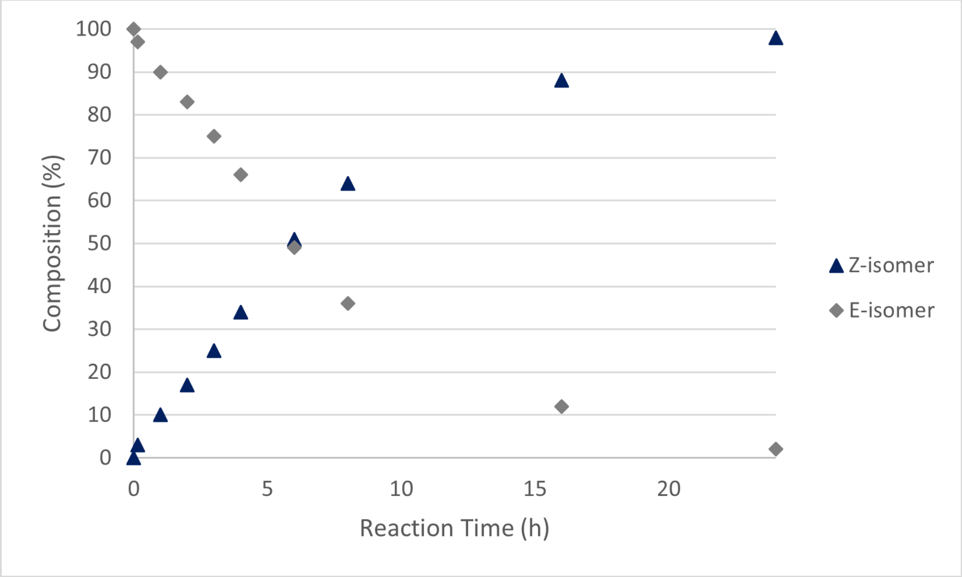

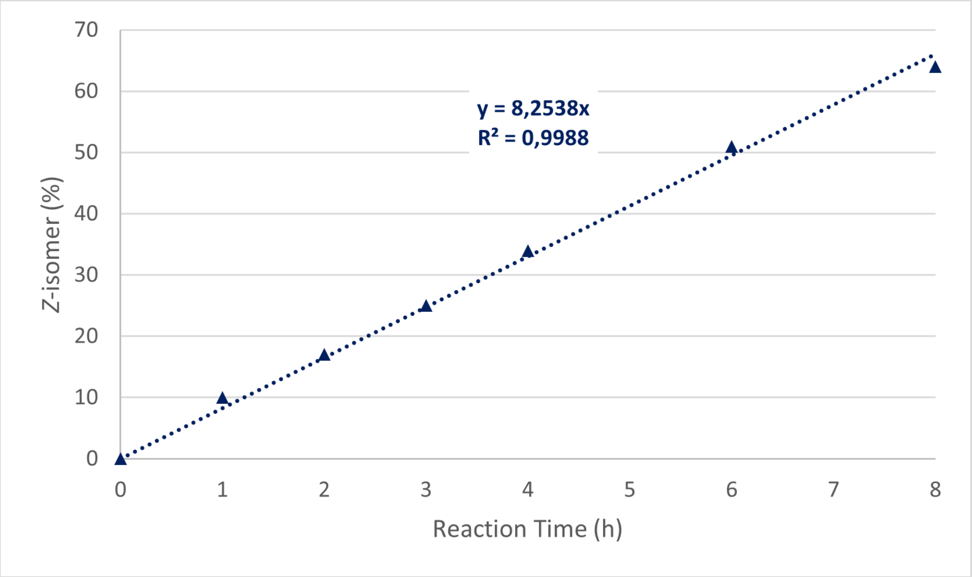

Effect of the substitution pattern

As our supposed mechanism is based on BINAP coordination on silicon, we wanted to know if we could proove that studying the effect of the substitution pattern with different substrates containing EDG or EWG in *para*-position. Yields are described as Y_R.

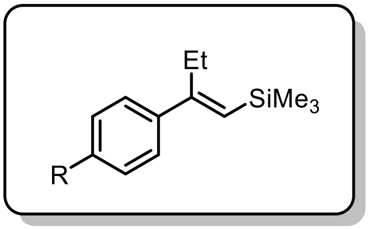

R	$Y_{R, 0\,h}$ (%)	$Y_{R, 1\,h}$ (%)	$Y_{R, 2\,h}$ (%)	$Y_{R, 3\,h}$ (%)	$Y_{R, 4\,h}$ (%)
**H**	0	10	17	25	34
**Me**	0	14	27	44	62
**F**	0	8	15	22	32
**CF_3**	0	2	6	13	17

Table 9: Effect of the substitution pattern with different EWG and EDG in para-position

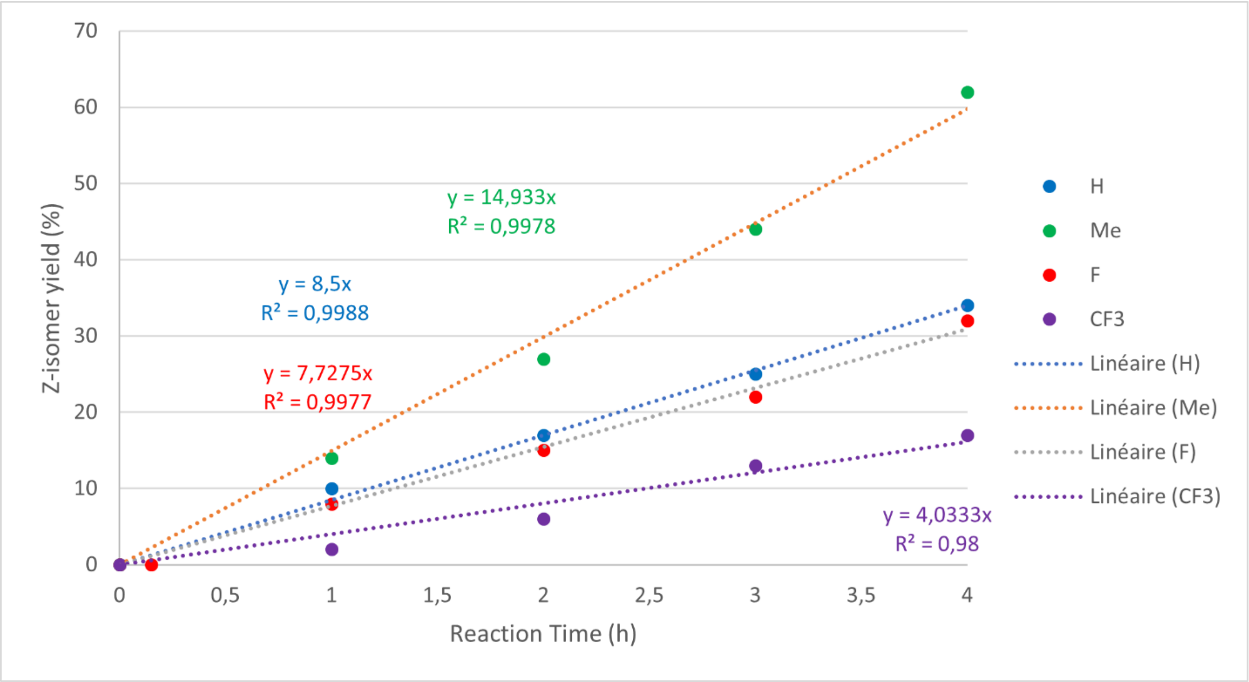

We have seen that the reaction was slower with an EWG in *para*-position. We then wanted to link this behaviour with Hammett constant (σ_R).

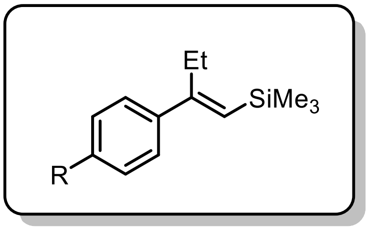

R	σ_R	dZ/dT	k_{rel}	log (k_{rel})
**H**	0	0,000000425	1	0
**Me**	-0.17	0,00000074667	1,756870588	0,244739772
**F**	0.06	0,0000003867	0,909882353	-0,041014758
**CF_3**	0.54	0,0000002017	0,474588235	-0,323683032

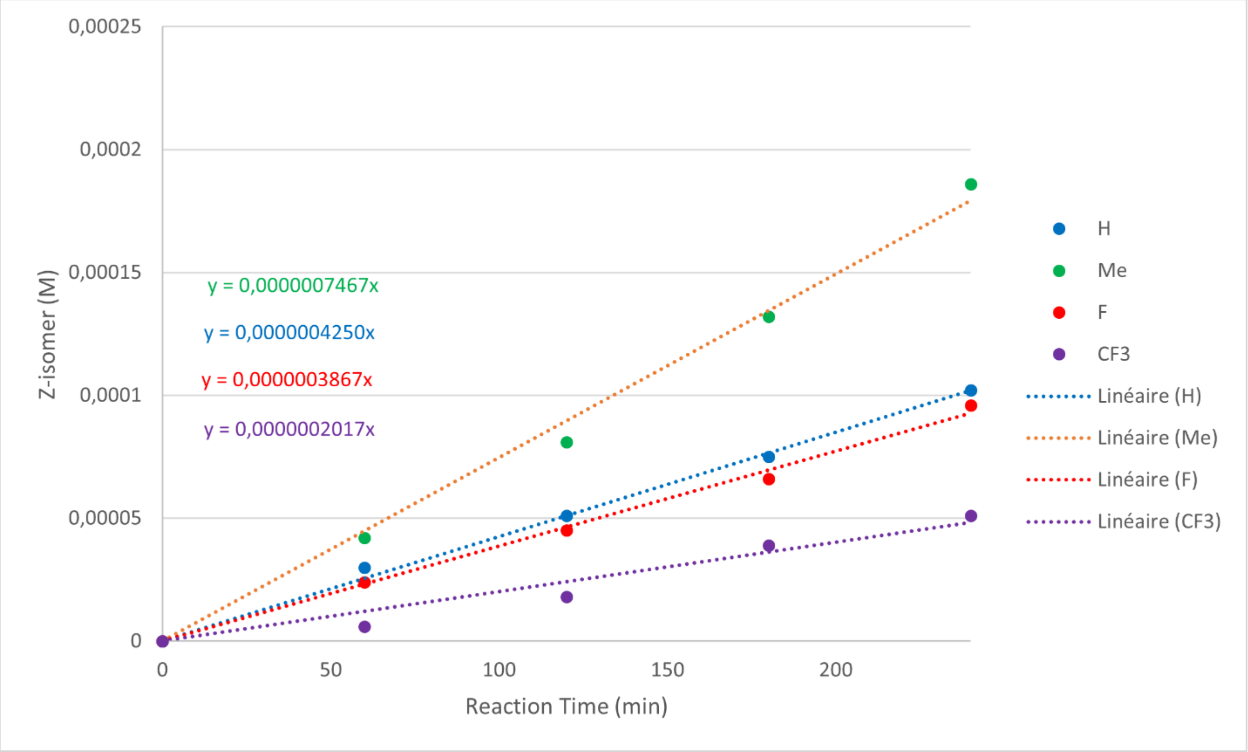

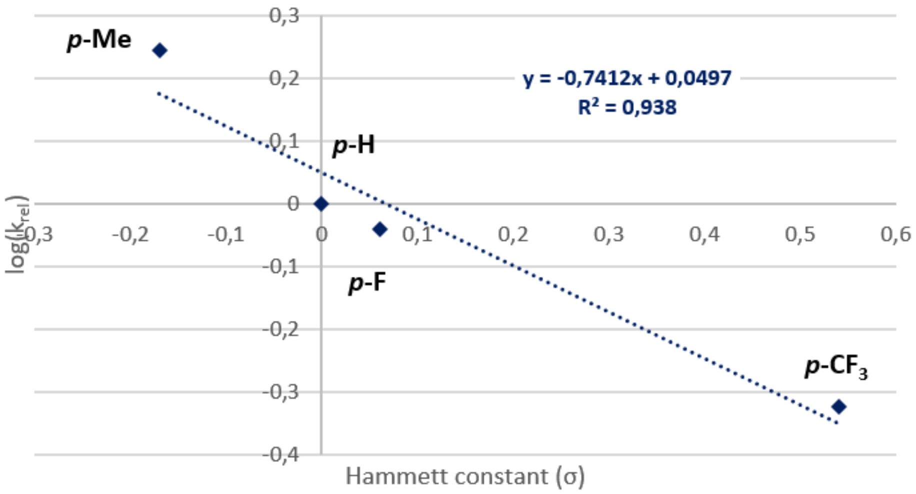

6. Isomerization of alkenyl silanes (*E*)-1 to (*E*)-18

Material

General procedure B

An oven-dried microwave tube equipped with a magnetic stir bar was charged with *rac*-BINAP (37.4 mg, 0.06 mmol, 0.2 equiv.) and the corresponding alkenyl silane derivative (0.3 mmol, 1 equiv.). The tube was sealed, evacuated and back-filled with argon 3 times. Then, DMSO (1 mL) was added by syringe and the reaction mixture was stirred for 24 h (unless otherwise stated) under visible light irradiation (405 nm). Then, the reaction mixture was diluted with EtOAc (10 mL) and washed with brine (3 x 10 mL). The organic phase was dried over $MgSO_4$, filtered, and concentrated under vacuo. The residue was then taken in Et_2O and filtered through a short plug of silica and silica was rinsed with Et_2O. Then, the filtrate was concentrated *in vacuo*. The yield was determined by mass recovery based on NMR-purity and the *E*-/*Z*-isomer ratio was determined by GC-FID analysis (unless otherwise stated).

General procedure C

An oven-dried microwave tube equipped with a magnetic stir bar was charged with *rac*-BINAP (37.4 mg, 0.06 mmol, 0.2 equiv.) and the corresponding alkenyl silane derivative (0.3 mmol, 1 equiv.). The tube was sealed, evacuated and back-filled with argon 3 times. Then, CH_3CN (1 mL) was added by syringe and the reaction mixture was stirred for 24 h (unless otherwise stated) under visible light irradiation (405 nm). Then, the reaction mixture was filtered through a short plug of silica and silica was rinsed with Et_2O. Then, the filtrate was concentrated *in vacuo*. The yield was determined by mass recovery based on NMR-purity and the *E*-/*Z*-isomer ratio was determined by GC-FID analysis (unless otherwise stated).

Characterization of (*Z*)-1 to (*Z*)-18

(*Z*)-trimethyl(2-phenylbut-1-en-1-yl)silane ((*Z*)-1)

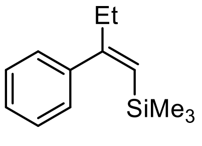

(*Z*)-1

Prepared according to general procedure **B** and obtained as a colorless oil (61.5 mg, quant. yield, *Z/E* = 98:2). ^{1}H NMR (300 MHz, $CDCl_3$) δ 7.43-7.21 (m, 3H), 7.21 – 7.03 (m, 2H), 5.57 (t, *J* = 1.4 Hz, 1H), 2.43 (qd, *J* = 7.4, 1.4 Hz, 2H), 1.04 (t, *J* = 7.4 Hz, 3H), -0.17 (s, 9H). ^{13}C NMR (75 MHz, $CDCl_3$) δ 161.4, 144.6, 128.1, 127.9, 126.9, 125.4, 35.4, 12.8, 0.2. These data were consistent with those reported in the literature.[1]

(*Z*)-trimethyl(2-phenylprop-1-en-1-yl)silane ((*Z*)-2)

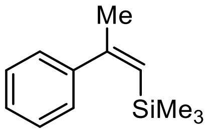

(*Z*)-2

Prepared according to general procedure **B** and obtained as a colorless oil (56 mg, 98% yield, *Z/E* = 93:7, determined by NMR 1H). ^{1}H NMR (300 MHz, $CDCl_3$) δ 7.58 – 7.37 (m, 3H), 7.41 – 7.30 (m, 2H), 5.77 (q, *J* = 1.4 Hz, 1H), 2.35 (d, *J* = 1.5 Hz, 3H), -0.00 (s, 9H). ^{13}C NMR (75 MHz, $CDCl_3$) δ 155.6, 144.9, 128.4, 128.0, 127.6, 127.1, 29.9, 0.2. These data were consistent with those reported in the literature. [1]

(*Z*)-trimethyl(2-phenylpenta-1,4-dien-1-yl)silane ((*Z*)-3)

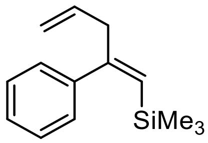

(*Z*)-3

Prepared according to general procedure **B** and obtained as a colorless oil (67 mg, quant. yield, *Z/E* = 98:2). ^{1}H NMR (300 MHz, $CDCl_3$) δ 7.60 – 7.32 (m, 3H), 7.33 – 7.12 (m, 2H), 6.13 – 5.83 (m, 1H), 5.72 (td, *J* = 1.4, 0.5 Hz, 1H), 5.21 – 5.14 (m, 1H), 5.18 – 5.09 (m, 1H), 3.27 (dd, *J* = 6.9, 1.5 Hz, 2H), -0.06 (s, 9H). ^{13}C NMR (75 MHz, $CDCl_3$) δ 157.6, 144.2, 136.0, 128.1, 128.1, 127.9, 127.1, 116.6, 46.9, 0.2.

(*E*)-trimethyl(2-phenyl-2-(tributylstannyl)vinyl)silane ((*E*)-4)

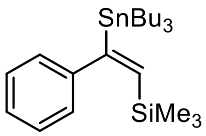

(*E*)-4

Prepared according to general procedure **C** and obtained as a colorless oil (141 mg, quant. yield, *E/Z* = 56:44). ^{1}H NMR (300 MHz, $CDCl_3$) δ 7.28 – 7.18 (m, 2H), 7.17 – 7.06 (m, 1H), 6.95 – 6.83 (m, 2H), 6.15 (s, 1H), 1.58 – 1.35 (m, 6H), 1.31 – 1.03 (m, 6H), 1.03 – 0.60 (m, 15H), -0.15 (s, 9H). ^{13}C NMR (75 MHz, $CDCl_3$) δ 168.8, 148.6, 146.1, 127.9, 126.1, 125.2, 29.2, 27.5, 13.8, 10.2, 0.5. These data were consistent with those reported in the literature. [1, 5]

(*Z*)-tert-butyldimethyl(styryl)silane ((*Z*)-5)

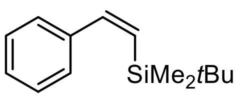

(*Z*)-5

Prepared according to general procedure **B** and obtained as a colorless oil (66 mg, quant. yield, *Z/E* = 35:65). ^{1}H NMR (300 MHz, $CDCl_3$) δ 7.57 – 7.36 (m, 1H), 7.36 – 7.16 (m, 5H), 5.87 (d, *J* = 15.4 Hz, 1H), 0.91 (s, 9H), -0.07 (s, 6H). These data were consistent with those reported in the literature. [1, 6]

---

[5] B. Huang, Z.Zhou, M. Z. Cai, *Chinese J. Chem.* **2006**, *24*, 1469-1471.

[6] S. Bratovanov, W. Koźmiński, J. Fässler, Z. Molnar, D. Nanz, S. Bienz, *Organometallics* **1997**, *16*, 3128–3134.

(*Z*)-dimethyl(2-phenylbut-1-en-1-yl)silanol ((*Z*)-6)

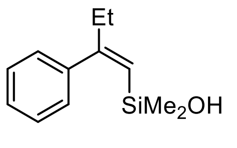

(*Z*)-6

Prepared according to general procedure **B** and obtained as a colorless oil (62.5mg, quant. yield, *Z/E* = 91:9, determined by NMR 1H). ^{1}H NMR (300 MHz, $CDCl_3$) δ 7.54 – 7.43 (m, 3H), 7.45 – 7.36 (m, 2H), 5.78 (t, *J* = 1.4 Hz, 1H), 2.65 (qd, *J* = 7.4, 1.4 Hz, 2H), 1.26 (t, *J* = 7.4 Hz, 3H), 0.00 (s, 6H). ^{13}C NMR (75 MHz, $CDCl_3$) δ 161.2, 144.3, 128.1, 127.8, 127.1, 125.8, 35.0, 12.7, 2.1.

(*Z*)-trimethyl(2-(p-tolyl)but-1-en-1-yl)silane ((*Z*)-7)

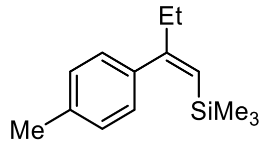

(*Z*)-7

Prepared according to general procedure **B** and obtained as a colorless oil (66.5 mg, quant. yield, *Z/E* = 98:2). ^{1}H NMR (300 MHz, $CDCl_3$) δ 7.28 (d, *J* = 7.8 Hz, 2H), 7.21 (d, *J* = 8.0 Hz, 2H), 5.70 (t, *J* = 1.5 Hz, 1H), 2.57 (qd, *J* = 7.3, 1.2 Hz, 2H), 2.52 (s, 3H), 1.18 (t, *J* = 7.4 Hz, 3H), 0.00 (s, 9H). ^{13}C NMR (75 MHz, $CDCl_3$) δ 161.6, 141.7, 136.6, 128.6, 128.1, 125.1, 35.5, 21.4, 12.9, 0.4.

(*Z*)-(2-(4-(tert-butyl)phenyl)but-1-en-1-yl)trimethylsilane ((*Z*)-8)

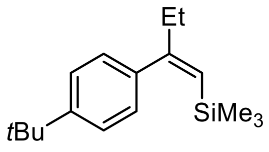

(*Z*)-8

Prepared according to general procedure **B** and obtained as a colorless oil (74.2 mg, 95% yield, *Z/E* = 97:3). ^{1}H NMR (300 MHz, $CDCl_3$) δ 7.38 – 7.27 (m, 2H), 7.17 – 6.95 (m, 2H), 5.51 (t, *J* = 1.4 Hz, 1H), 2.40 (qd, *J* = 7.4, 1.5 Hz, 2H), 1.32 (s, 9H), 1.02 (t, *J* = 7.4 Hz, 3H), -0.20 (s, 9H). ^{13}C NMR (75 MHz, $CDCl_3$) δ 161.5, 149.9, 141.6, 127.7, 125.0, 124.6, 35.2, 34.6, 31.6, 12.8, 0.3.

(*Z*)-trimethyl(2-(m-tolyl)but-1-en-1-yl)silane ((*Z*)-9)

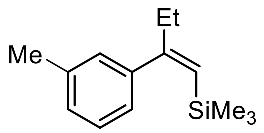

(*Z*)-9

Prepared according to general procedure **B** and obtained as a colorless oil (62.2 mg, 95% yield, *Z/E* = 96:4. ^{1}H NMR (300 MHz, $CDCl_3$) δ 7.19 (t, *J* = 7.5 Hz, 1H), 7.08 (d, *J* = 7.5 Hz, 1H), 7.04 – 6.85 (m, 2H), 5.53 (t, *J* = 1.4 Hz, 1H), 2.42 (qd, *J* = 7.3, 1.4 Hz, 2H), 2.36 (s, 3H), 1.03 (t, *J* = 7.4 Hz, 3H), -0.17 (s, 9H). ^{13}C NMR (75 MHz, $CDCl_3$) δ 161.6, 144.5, 137.3, 128.9, 127.7, 127.6, 125.1, 125.0, 35.3, 21.5, 12.8, 0.3.

(*Z*)-(2-([1,1'-biphenyl]-4-yl)but-1-en-1-yl)trimethylsilane ((*Z*)-10)

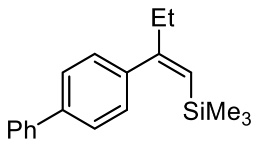

(*Z*)-10

Prepared according to general procedure **B** and obtained as a colorless oil (71.2 mg, 85% yield, *Z/E* = 90:10). ^{1}H NMR (300 MHz, $CDCl_3$) δ 7.81 – 7.72 (m, 2H), 7.74 – 7.64 (m, 2H), 7.63 – 7.52 (m, 3H), 7.52 – 7.44 (m, 1H), 7.42 – 7.31 (m, 1H), 5.74 (t, *J* = 1.4 Hz, 1H), 2.59 (qd, *J* = 7.4, 1.4 Hz, 2H), 1.19 (t, *J* = 7.4 Hz, 3H), 0.00 (s, 9H). ^{13}C NMR (75 MHz, $CDCl_3$) δ 161.0, 143.6, 141.1, 139.8, 128.9, 128.6, 127.3, 127.1, 126.5, 125.7, 35.3, 12.8, 0.4.

(*Z*)-trimethyl(2-(4-(methylthio)phenyl)but-1-en-1-yl)silane ((*Z*)-11)

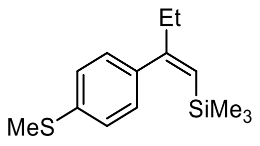

(*Z*)-11

Prepared according to general procedure **B** and obtained as a colorless oil (75.1 mg, 85% yield, *Z/E* = 94:6). ^{1}H NMR (300 MHz, $CDCl_3$) δ 7.24 – 7.13 (m, 2H), 7.12 – 7.03 (m, 2H), 5.55 (t, *J* = 1.4 Hz, 1H), 2.49 (s, 3H), 2.39 (qd, *J* = 7.4, 1.4 Hz, 2H), 1.00 (t, *J* = 7.4 Hz, 3H), -0.16 (s, 9H). ^{13}C NMR (75 MHz, $CDCl_3$) δ 160.7, 141.4, 136.9, 128.6, 126.1, 125.6, 35.3, 16.1, 12.8, 0.3.

(*Z*)-trimethyl(2-(4-(trifluoromethoxy)phenyl)but-1-en-1-yl)silane ((*Z*)-12)

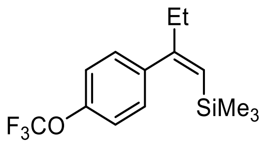

(*Z*)-12

Prepared according to general procedure **C** and obtained as a colorless oil (87 mg, quant. yield, *Z/E* = 90:10). ^{1}H NMR (300 MHz, $CDCl_3$) δ 7.19 – 7.13 (m, 4H), 5.60 (t, *J* = 1.4 Hz, 1H), 2.39 (qd, *J* = 7.4, 1.5 Hz, 2H), 1.02 (t, *J* = 7.4 Hz, 3H), -0.18 (s, 9H). ^{19}F NMR (282 MHz, $CDCl_3$) δ -58.44. ^{13}C NMR (75 MHz, $CDCl_3$) δ 159.8, 148.4 (q, *J* = 1.7 Hz), 143.3, 129.5, 126.6, 120.7 (q, *J* = 256.7 Hz), 120.4, 35.4, 12.7, 0.2.

(*Z*)-trimethyl(2-(4-(4,4,5,5-tetramethyl-1,3,2-dioxaborolan-2-yl)phenyl)but-1-en-1-yl)silane ((*Z*)-13)

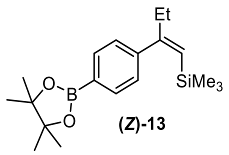

(*Z*)-13

Prepared according to general procedure **B** and obtained as a colorless oil (79.3 mg, 80% yield, *Z/E* = 93:7). ^{1}H NMR (300 MHz, $CDCl_3$) δ 7.74 (d, *J* = 7.7 Hz, 2H), 7.15 (d, *J* = 7.8 Hz, 2H), 5.55 (t, *J* = 1.5 Hz, 1H), 2.40 (qd, *J* = 7.4, 1.4 Hz, 2H), 1.35 (s, 12H), 0.99 (t, *J* = 7.4 Hz, 3H), -0.19 (s, 9H). ^{13}C NMR (75 MHz, $CDCl_3$) δ 161.3, 147.6, 134.4, 127.5, 125.6, 83.9, 35.3, 25.1, 25.0, 12.7, 0.3.

(*Z*)-(2-(4-fluorophenyl)but-1-en-1-yl)trimethylsilane ((*Z*)-14)

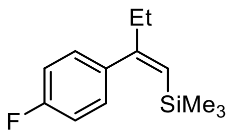

(*Z*)-14

Prepared according to general procedure **B** and obtained as a colorless oil (60 mg, 90% yield, *E/Z* = 93:7, E/Z ratio was determined by ^{1}H NMR). ^{1}H NMR (300 MHz, $CDCl_3$) δ 7.37 – 7.22 (m, 2H), 7.22 – 7.08 (m, 2H), 5.74 (t, *J* = 1.4 Hz, 1H), 2.56 (qd, *J* = 7.4, 1.4 Hz, 2H), 1.18 (td, *J* = 7.5, 1.2 Hz, 3H), -0.00 (s, 9H). ^{19}F NMR (282 MHz, $CDCl_3$) δ -116.54. ^{13}C NMR (75 MHz, $CDCl_3$) δ ^{13}C NMR (75 MHz, $CDCl_3$) δ 162.3 (d, *J* = 245.9 Hz), 158.1, 139.5 (d, *J* = 3.3 Hz), 128.0 (d, *J* = 7.7 Hz), 127.4 (d, *J* = 0.8 Hz), 115.0 (d, *J* = 21.2 Hz), 28.1, 14.2, 0.3.

(*Z*)-trimethyl(2-(4-(trifluoromethyl)phenyl)but-1-en-1-yl)silane ((*Z*)-15)

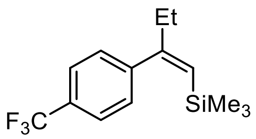

(*Z*)-15

Prepared according to general procedure **C** and obtained as a colorless oil (82.4 mg, quant. yield, *Z/E* = 94:6). ^{1}H NMR (300 MHz, $CDCl_3$) δ 7.70 – 7.42 (m, 2H), 7.33 – 7.15 (m, 2H), 5.64 (t, *J* = 1.5 Hz, 1H), 2.41 (qd, *J* = 7.4, 1.5 Hz, 2H), 1.02 (t, *J* = 7.4 Hz, 3H), -0.18 (s, 9H). ^{19}F NMR (282 MHz, $CDCl_3$) δ -62.83. ^{13}C NMR (75 MHz, $CDCl_3$) δ 159.7, 148.4, 129.3 (q, *J* = 32.5 Hz), 128.5, 126.9, 124.9 (q, *J* = 3.9 Hz), 124.46 (q, *J* = 272.1 Hz), 35.3, 12.6, 0.2.

(*Z*)-(2-(4-bromophenyl)but-1-en-1-yl)trimethylsilane ((*Z*)-16)

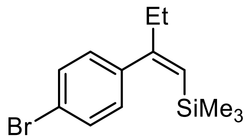

(*Z*)-16

Prepared according to general procedure **B** and obtained as a colorless oil (75.6 mg, 89% yield, *Z/E* = 83:17). ^{1}H NMR (300 MHz, $CDCl_3$) δ 7.60 (d, *J* = 8.3 Hz, 2H), 7.19 (d, *J* = 8.3 Hz, 2H), 5.75 (t, *J* = 1.5 Hz, 1H), 2.54 (qd, *J* = 7.4 Hz, 1.4 Hz, 2H), 1.16 (t, *J* = 7.4 Hz, 3H), -0.00 (s, 9H). ^{13}C NMR (75 MHz, $CDCl_3$) δ 159.9, 143.4, 131.0, 129.8, 126.3, 120.9, 35.3, 12.7, 0.3.

(Z)-trimethyl(2-(4-(methylsulfonyl)phenyl)but-1-en-1-yl)silane ((Z)-17)

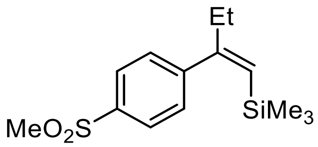

(*Z*)-17

Prepared according to general procedure **B** and obtained as a colorless oil (34 mg, quant. yield, *Z/E* = 79:21). ^{1}H NMR (300 MHz, $CDCl_3$) δ 7.93 – 7.78 (m, 2H), 7.45 – 7.28 (m, 2H), 5.65 (t, *J* = 1.4 Hz, 1H), 3.05 (s, 3H), 2.39 (qd, *J* = 7.4, 1.4 Hz, 2H), 1.05 – 0.93 (m, 3H), -0.21 (s, 9H). ^{13}C NMR (75 MHz, $CDCl_3$) δ 158.9, 150.5, 139.0, 129.0, 127.5, 127.0, 44.7, 35.0, 12.5, 0.1.

(*Z*)-trimethyl(2-(naphthalen-2-yl)but-1-en-1-yl)silane ((*Z*)-18)

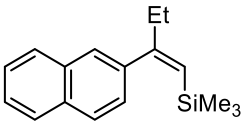

(*Z*)-18

Prepared according to general procedure **B** and obtained as a colorless oil (69.5 mg, 91% yield, *Z/E* = 94:6). ^{1}H NMR (300 MHz, $CDCl_3$) δ 7.54 – 7.36 (m, 4H), 7.17 – 7.04 (m, 2H), 7.03 – 6.85 (m, 1H), 5.31 (s, 1H), 2.22 – 2.09 (m, 2H), 0.70 (t, *J* = 7.4 Hz, 3H), -0.53 (s, 9H). ^{13}C NMR (75 MHz, $CDCl_3$) δ 161.3, 142.0, 133.2, 132.6, 128.0, 127.8, 127.5, 126.8, 126.6, 126.2, 126.1, 125.7, 35.3, 12.9, 0.4.

7. References

[1] S. I. Faßbender, J. J. Molloy, C. Mück-Lichtenfeld, R. Gilmour, *Angew. Chem.* **2019**, *131*, 18792–18799.

[2] S. S. P. Chou, H. L. Kuo, C. J. Wang, C. Y. Tsai, C. M. Sun, *J. Org. Chem.* **1989**, *54*, 868-872.

[3] F. Sasaki, T. Endo, M. Noguchi, K. Kawai and T. Nakano, *Appl. Organometal. Chem.* **2008**, *22*, 128–138.

[4] I. Hemeon, R. D. Singer, *J. Mol. Catal. A Chem.* **2004**, *214*, 33-44.

[5] B. Huang, Z.Zhou, M. Z. Cai, *Chinese J. Chem.* **2006**, *24*, 1469-1471.

[6] S. Bratovanov, W. Koźmiński, J. Fässler, Z. Molnar, D. Nanz, S. Bienz, *Organometallics* **1997**, *16*, 3128–3134.

8. NMR Spectra

Starting materials

(E)-trimethyl(2-phenylbut-1-en-1-yl)silane ((E)-1)

^{1}H NMR (300 MHz, $CDCl_3$)

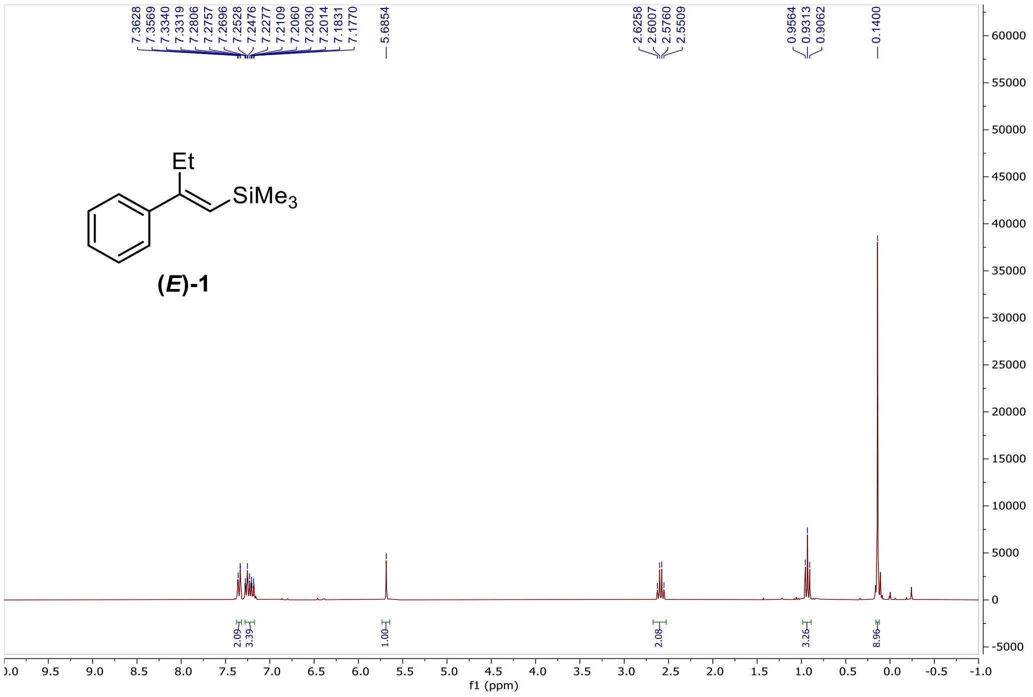

^{13}C NMR (75 MHz, $CDCl_3$)

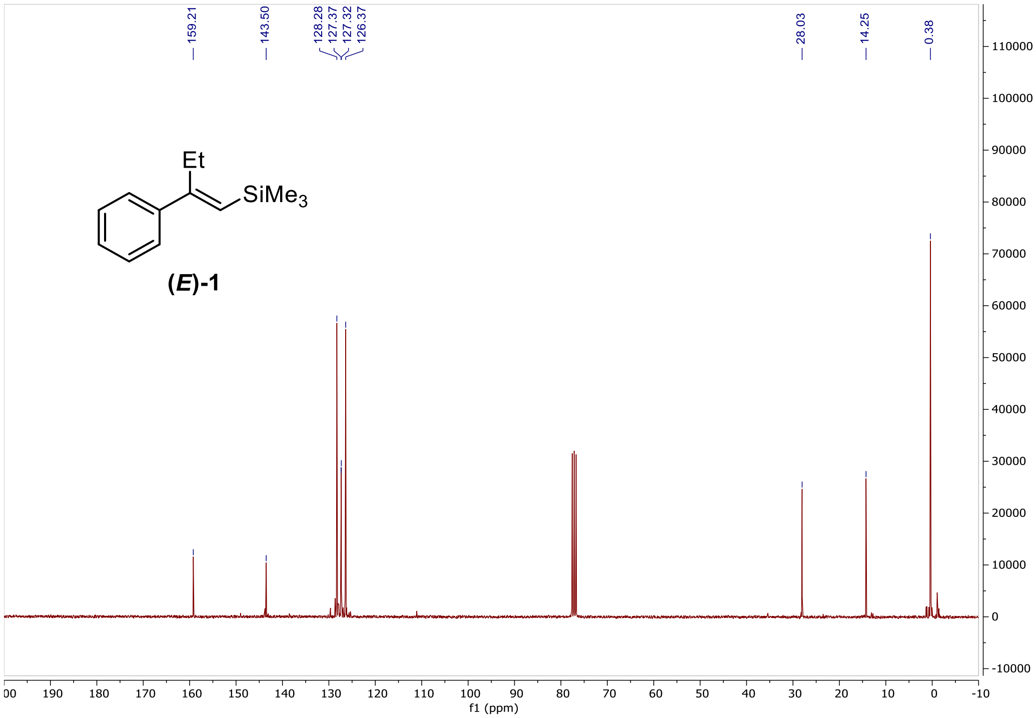

(*E*)-trimethyl(2-phenylprop-1-en-1-yl)silane ((*E*)-2)

^{1}H NMR (300 MHz, $CDCl_3$)

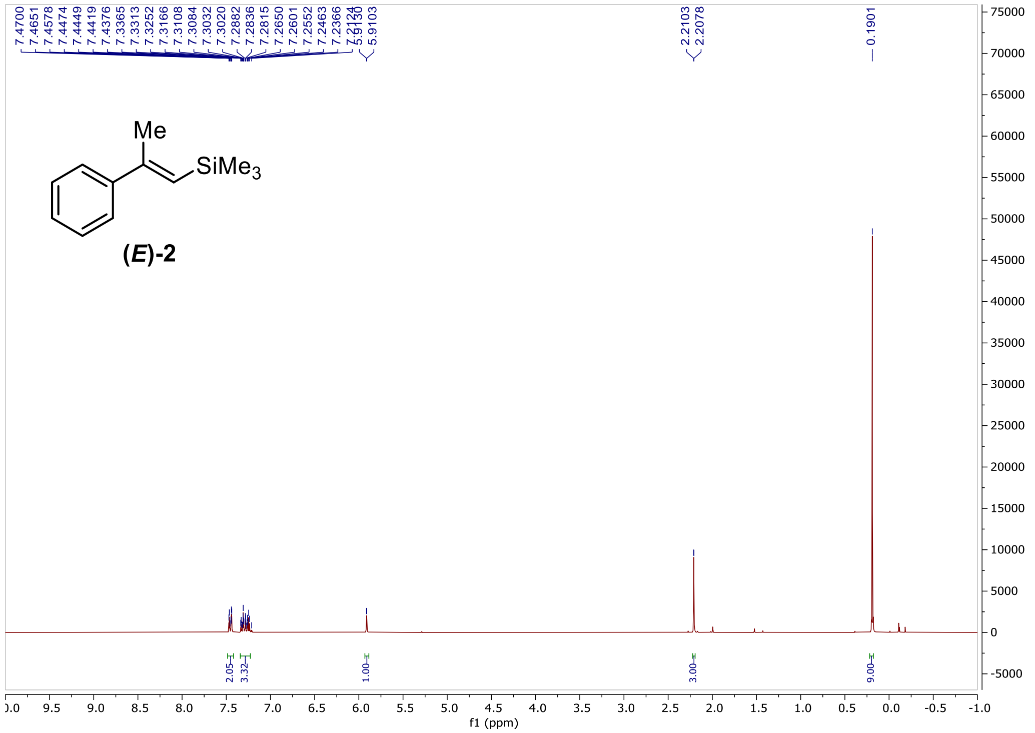

^{13}C NMR (75 MHz, $CDCl_3$)

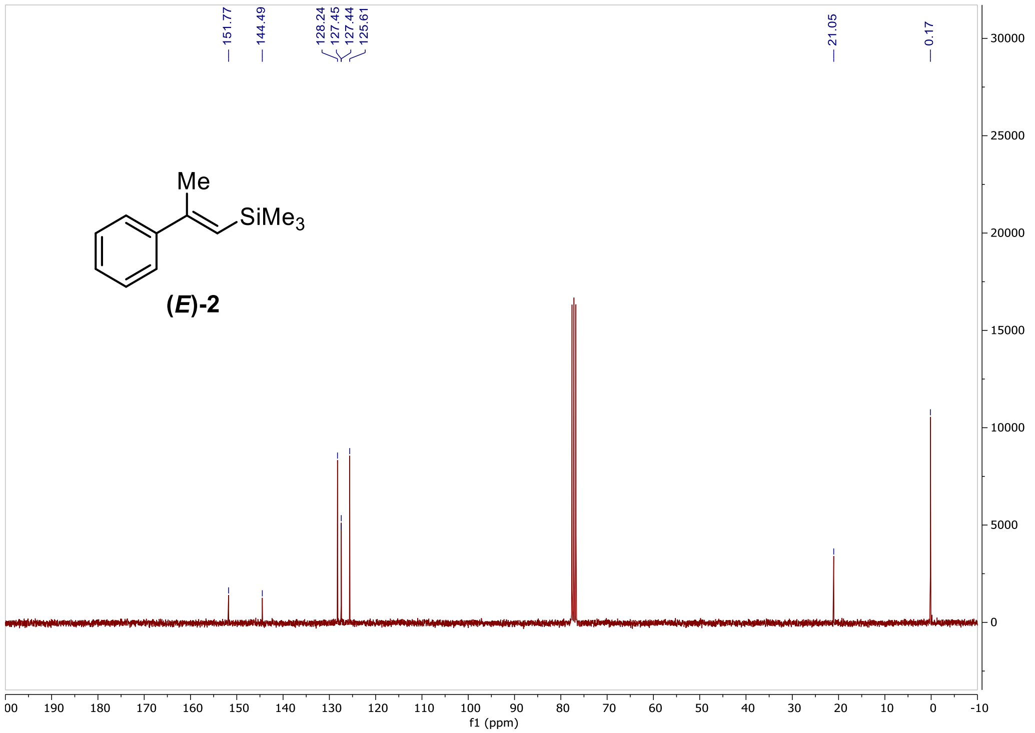

(*E*)-trimethyl(2-phenylpenta-1,4-dien-1-yl)silane ((*E*)-3)

^{1}H NMR (300 MHz, $CDCl_3$)

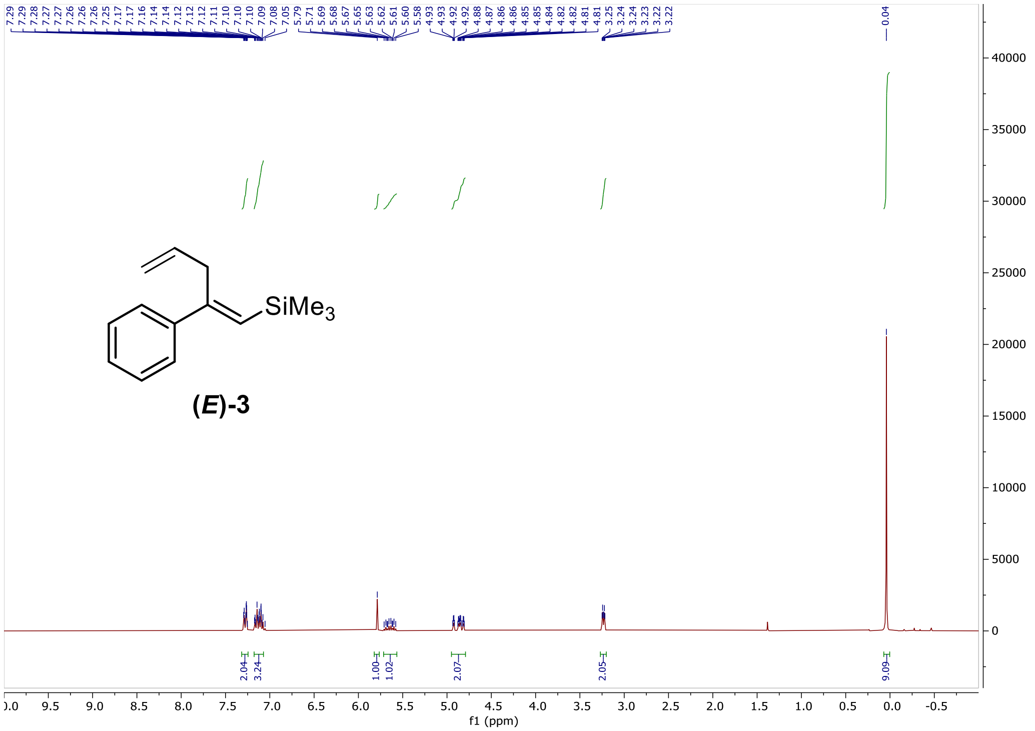

^{13}C NMR (75 MHz, $CDCl_3$)

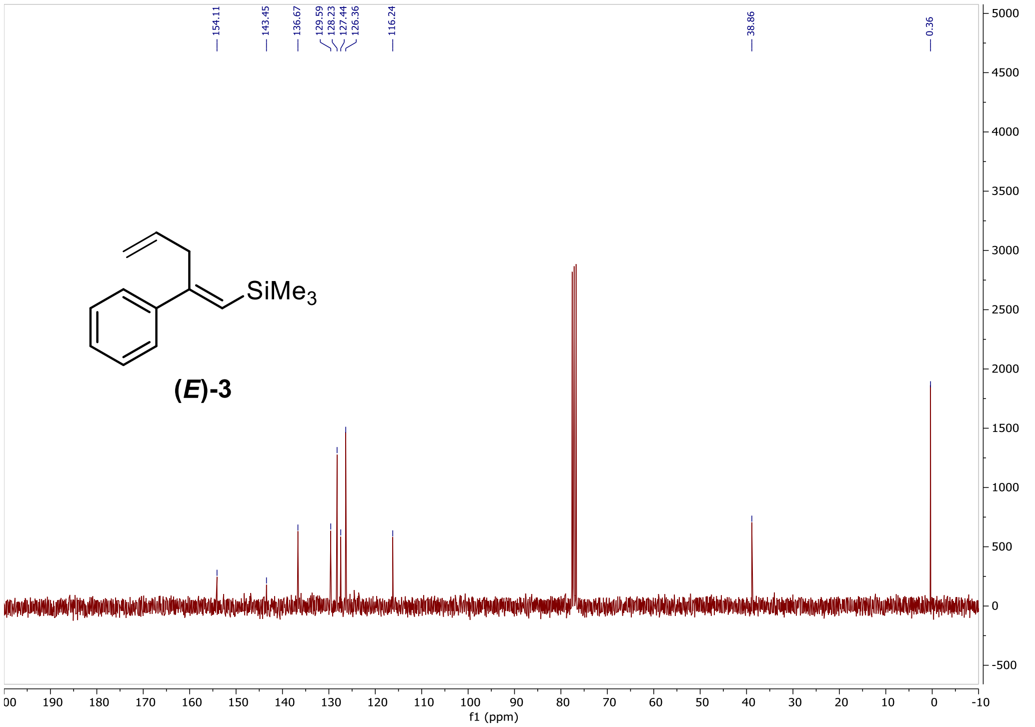

(Z)-trimethyl(2-phenyl-2-(tributylstannyl)vinyl)silane ((Z)-4)

^{1}H NMR (300 MHz, $CDCl_3$)

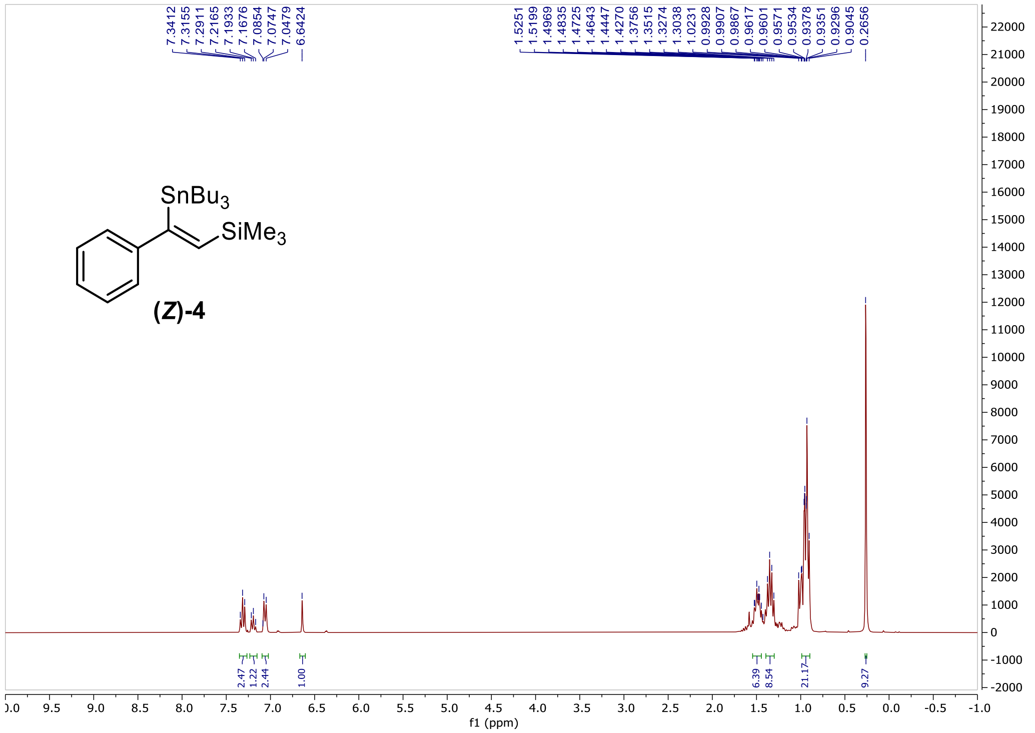

^{13}C NMR (75 MHz, $CDCl_3$)

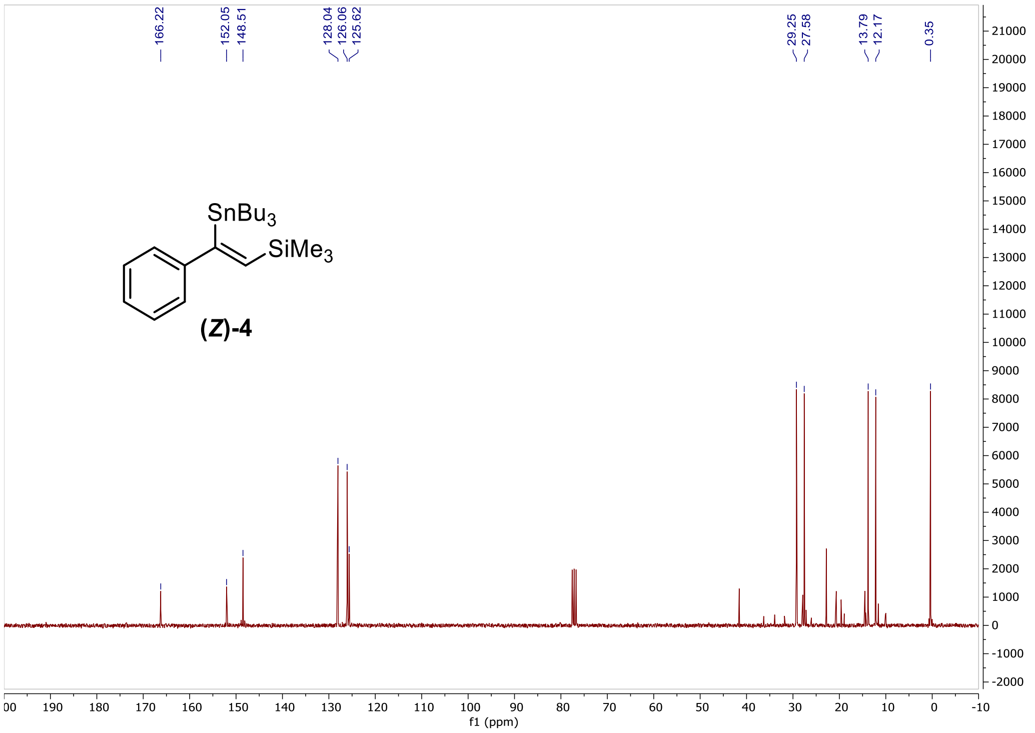

(*E*)-tert-butyldimethyl(styryl)silane ((*E*)-5)

^{1}H NMR (300 MHz, $CDCl_3$)

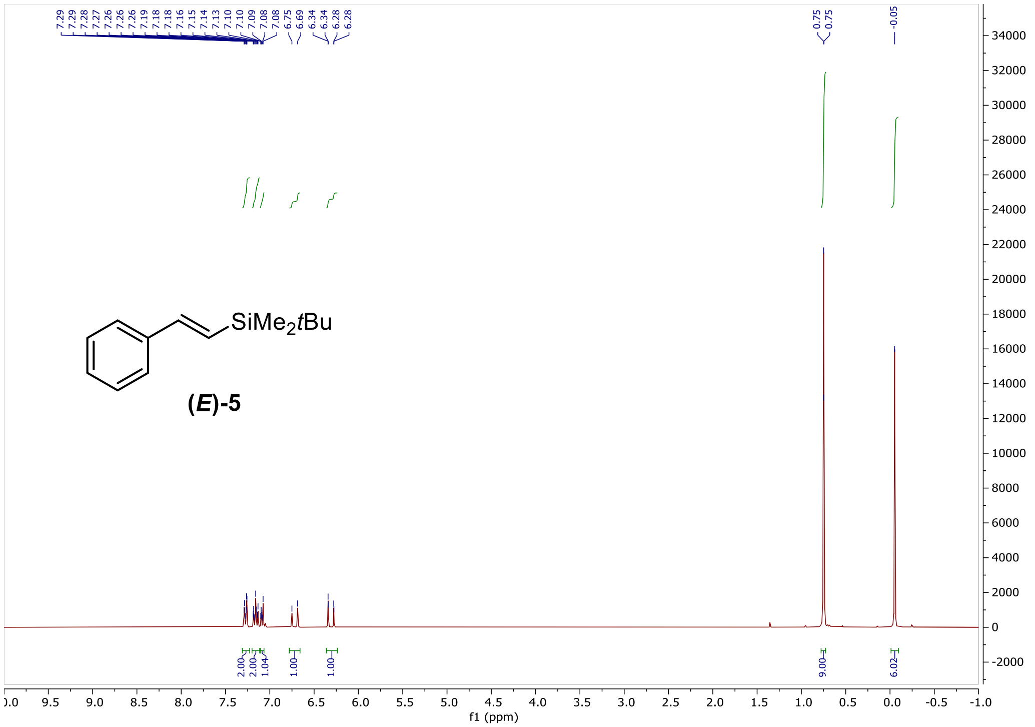

^{13}C NMR (75 MHz, $CDCl_3$)

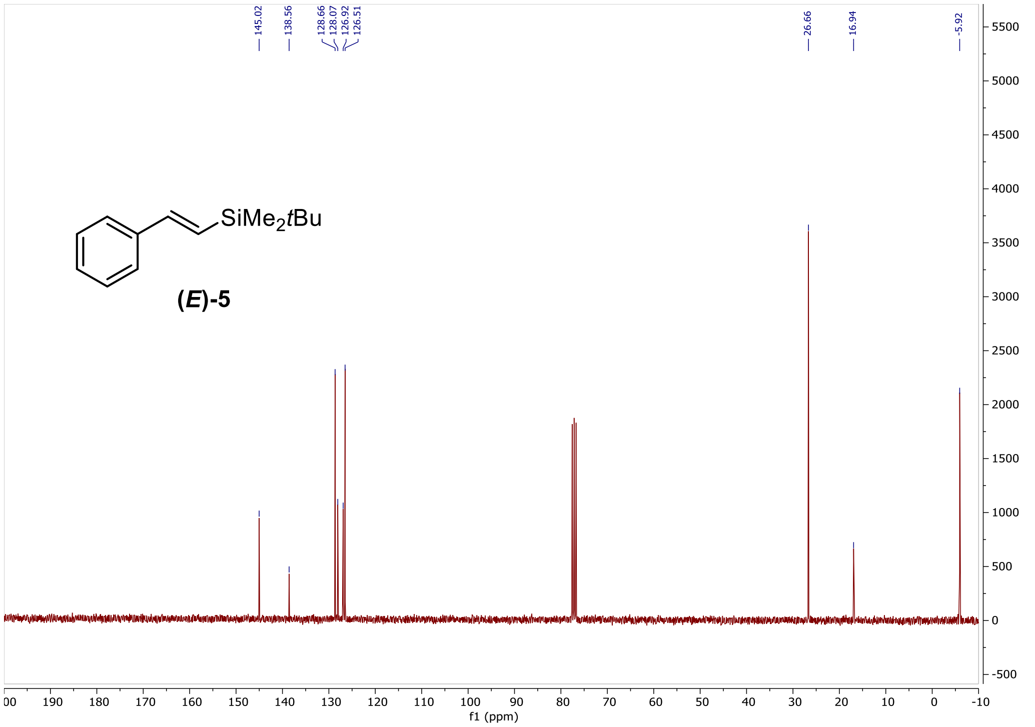

(*E*)-dimethyl(2-phenylbut-1-en-1-yl)silanol ((*E*)-6)

^{1}H NMR (300 MHz, $CDCl_3$)

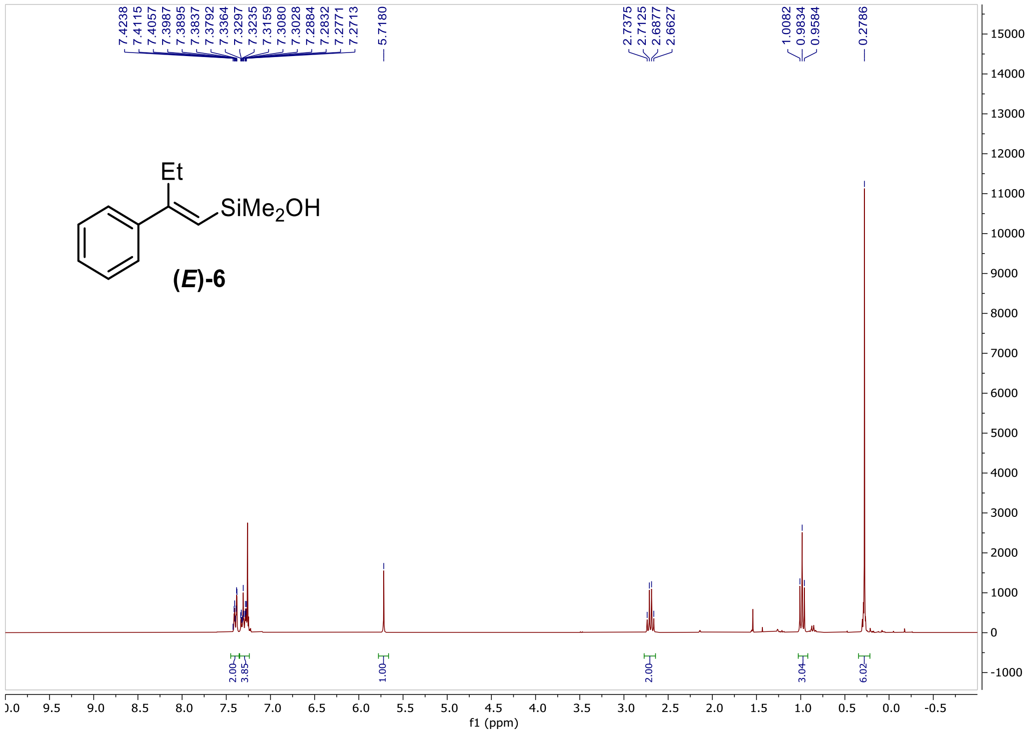

^{13}C NMR (75 MHz, $CDCl_3$)

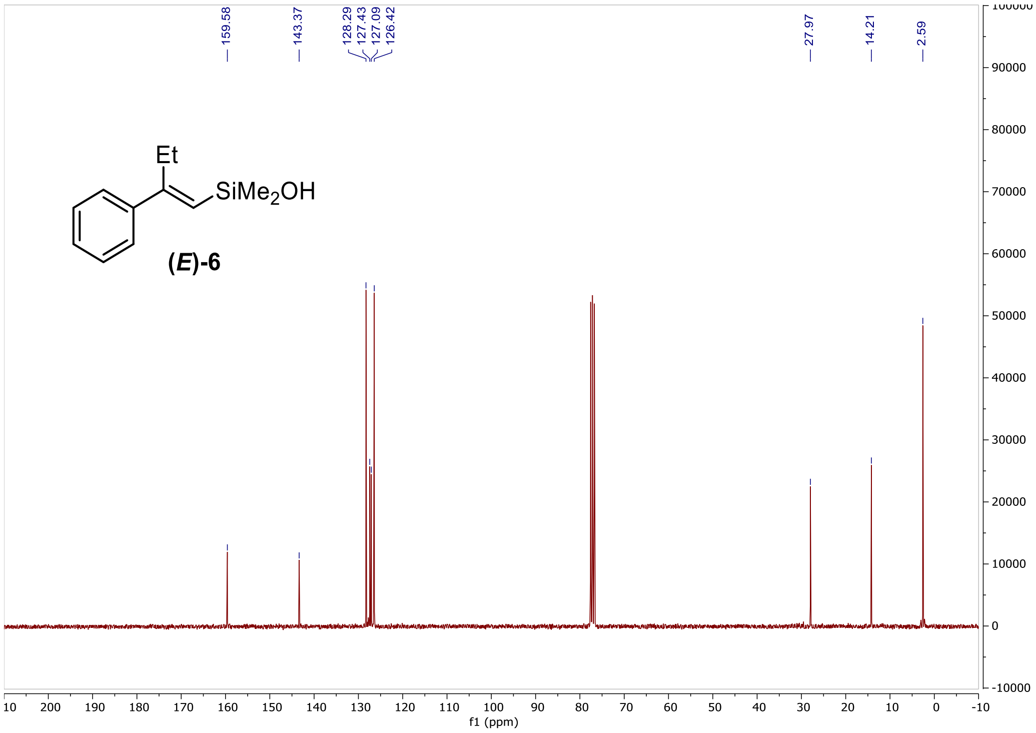

(*E*)-trimethyl(2-(p-tolyl)but-1-en-1-yl)silane ((*E*)-7)

^{1}H NMR (300 MHz, $CDCl_3$)

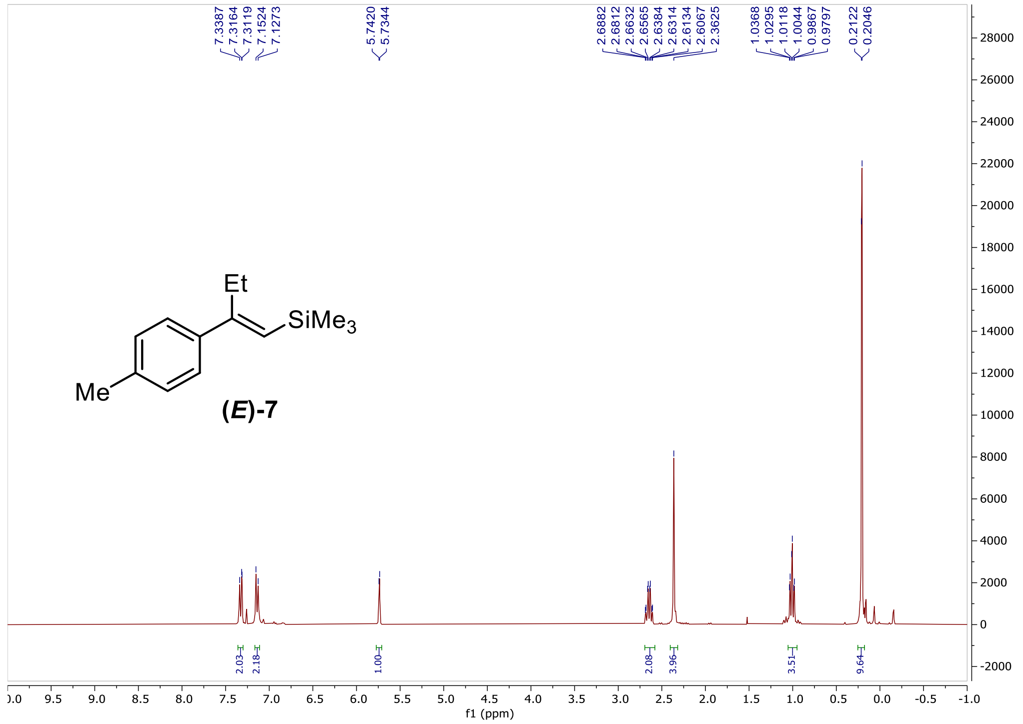

^{13}C NMR (75 MHz, $CDCl_3$)

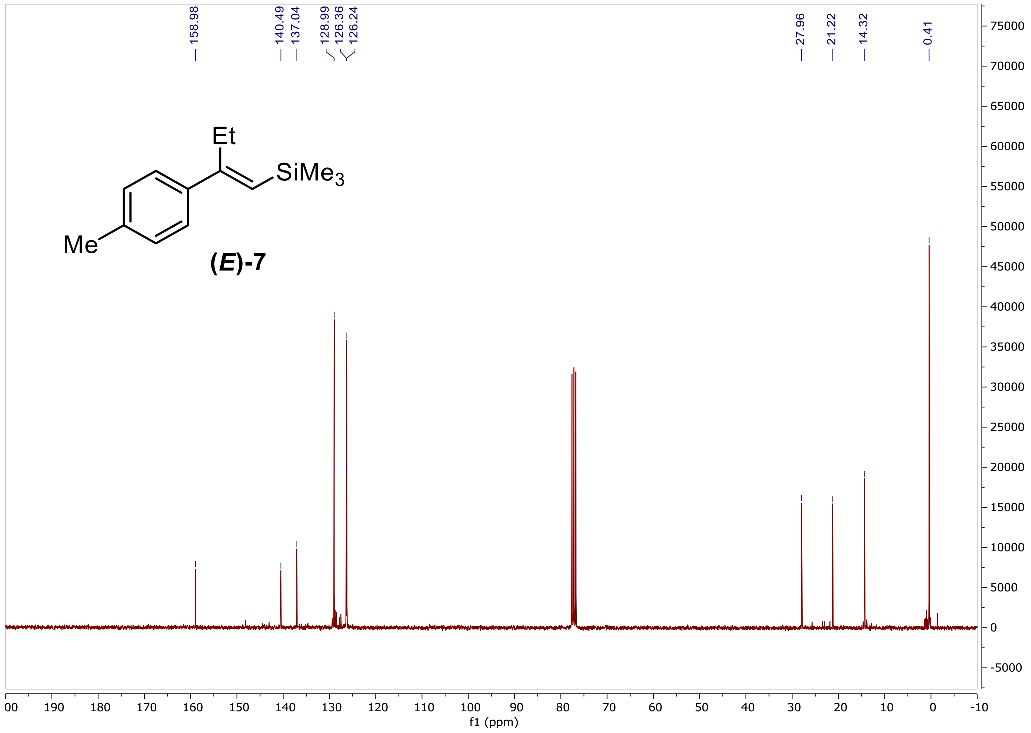

(*E*)-(2-(4-(tert-butyl)phenyl)but-1-en-1-yl)trimethylsilane ((*E*)-8)

^{1}H NMR (300 MHz, $CDCl_3$)

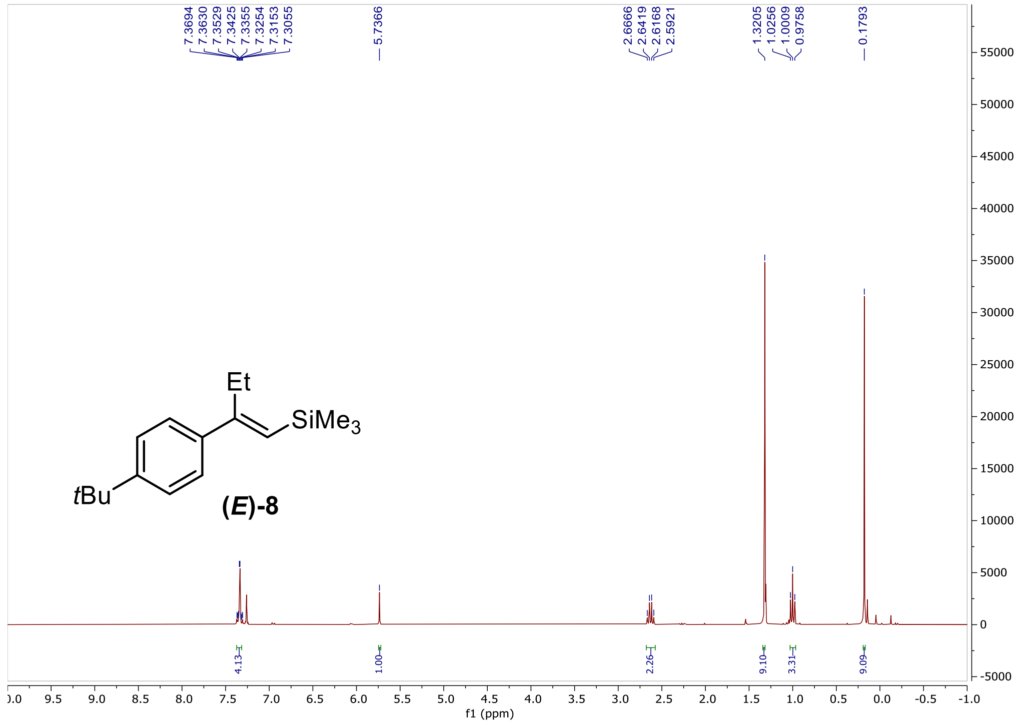

^{13}C NMR (75 MHz, $CDCl_3$)

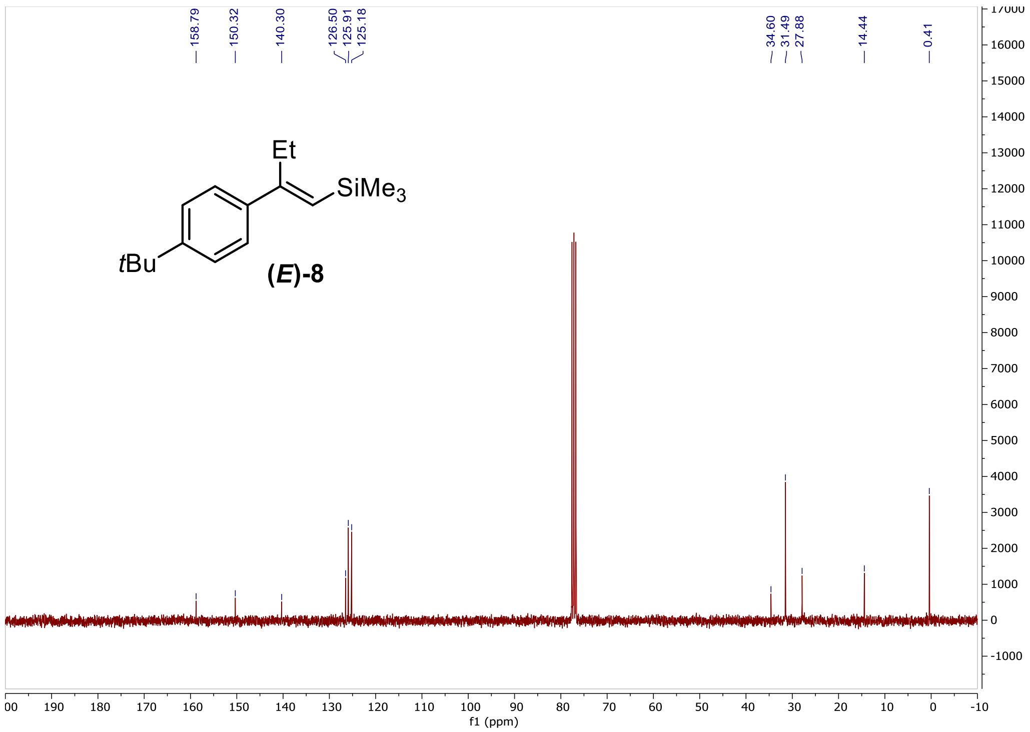

(*E*)-trimethyl(2-(m-tolyl)but-1-en-1-yl)silane ((*E*)-9)

^{1}H NMR (300 MHz, $CDCl_3$)

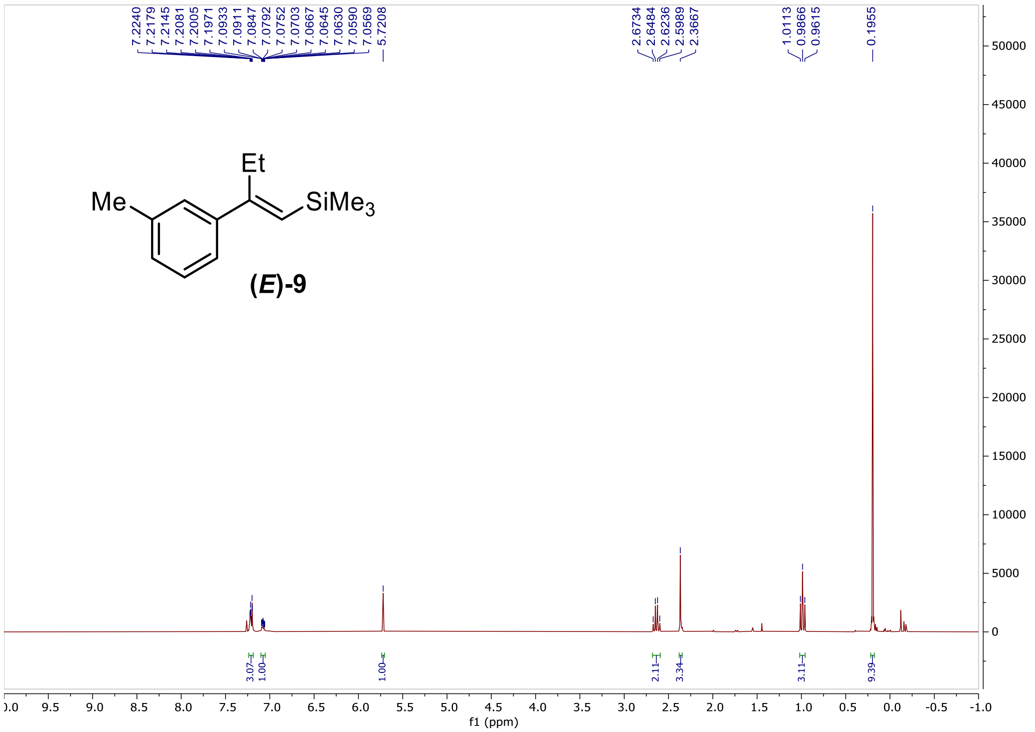

^{13}C NMR (75 MHz, $CDCl_3$)

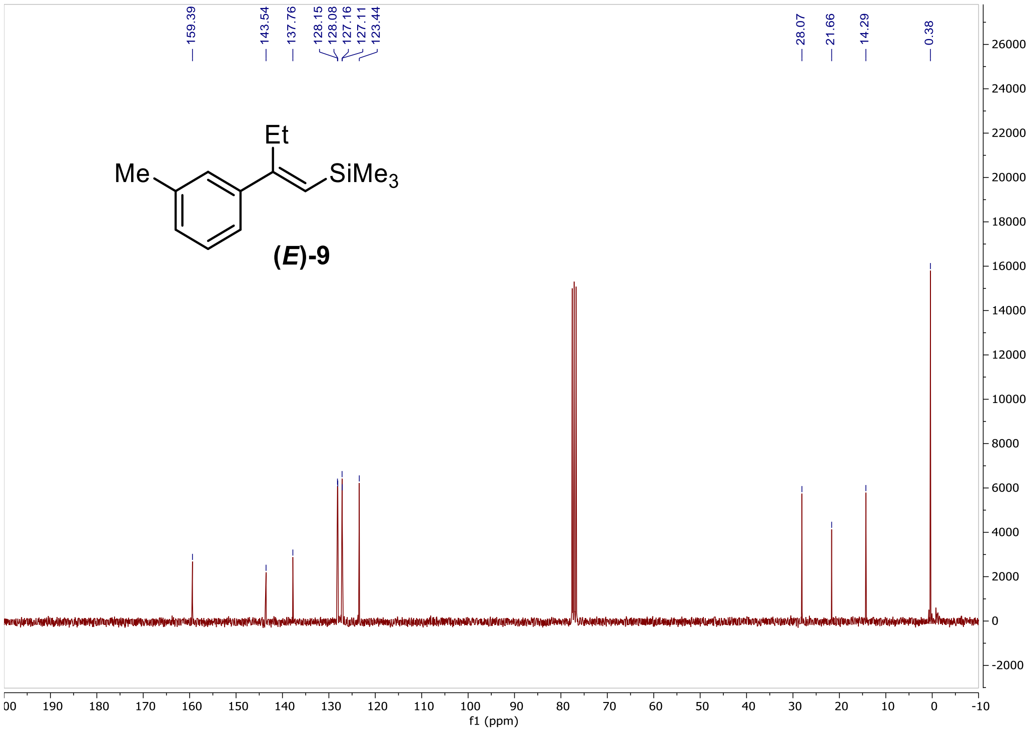

(*E*)-(2-([1,1'-biphenyl]-4-yl)but-1-en-1-yl)trimethylsilane ((*E*)-10)

^{1}H NMR (300 MHz, $CDCl_3$)

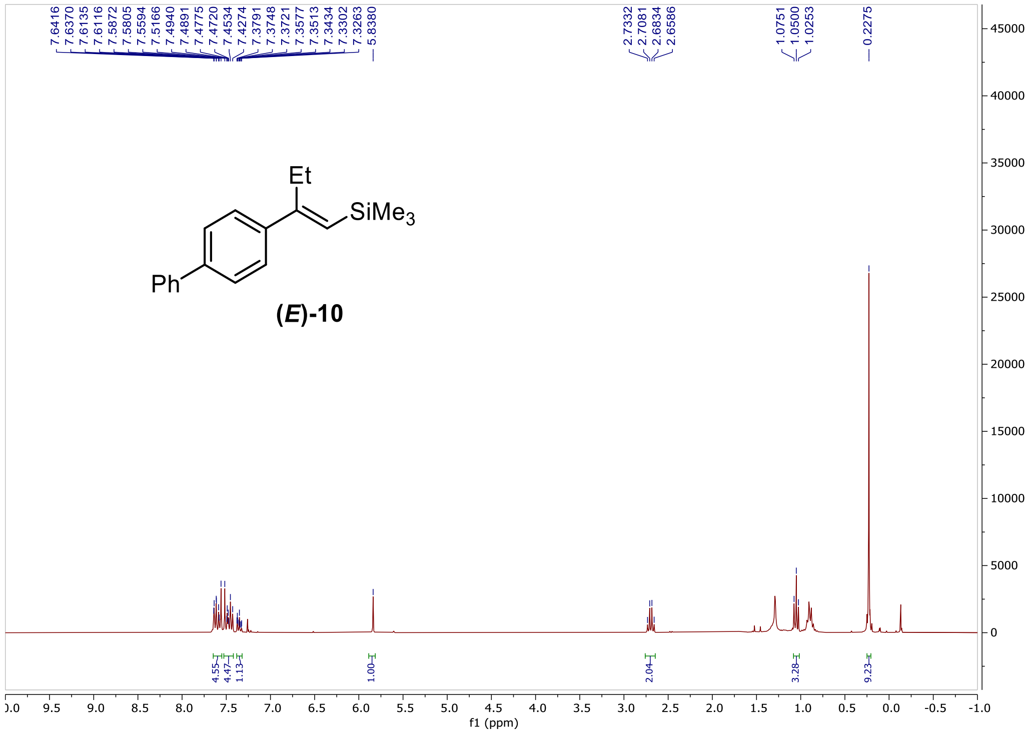

^{13}C NMR (75 MHz, $CDCl_3$)

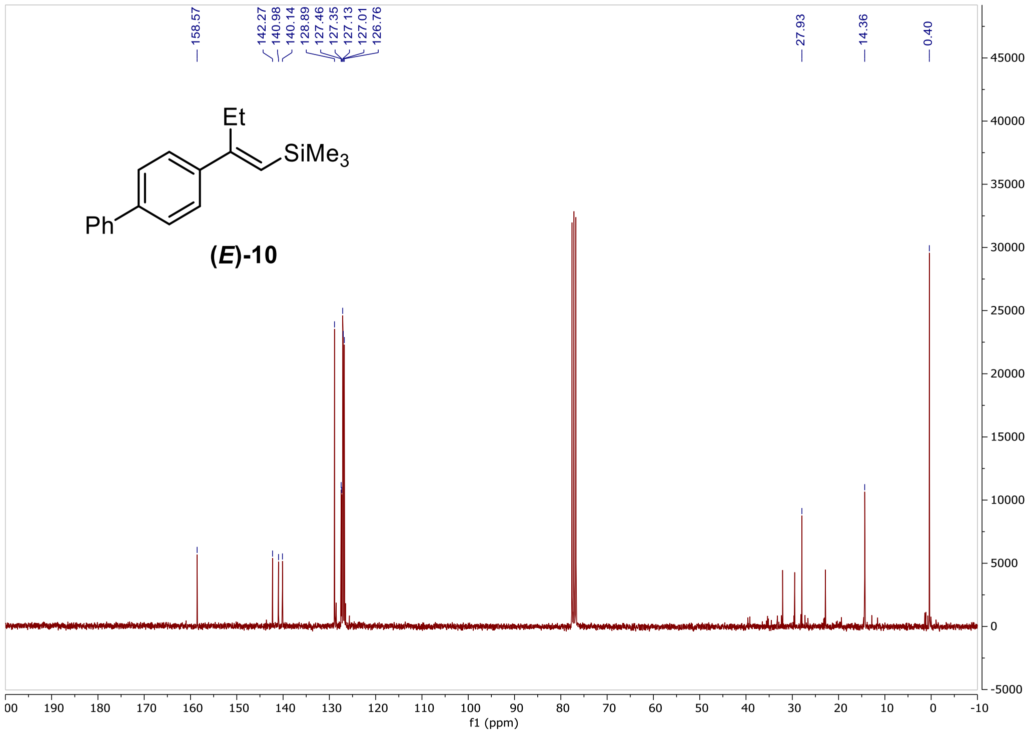

(*E*)-trimethyl(2-(4-(methylthio)phenyl)but-1-en-1-yl)silane ((*E*)-11)

^{1}H NMR (300 MHz, $CDCl_3$)

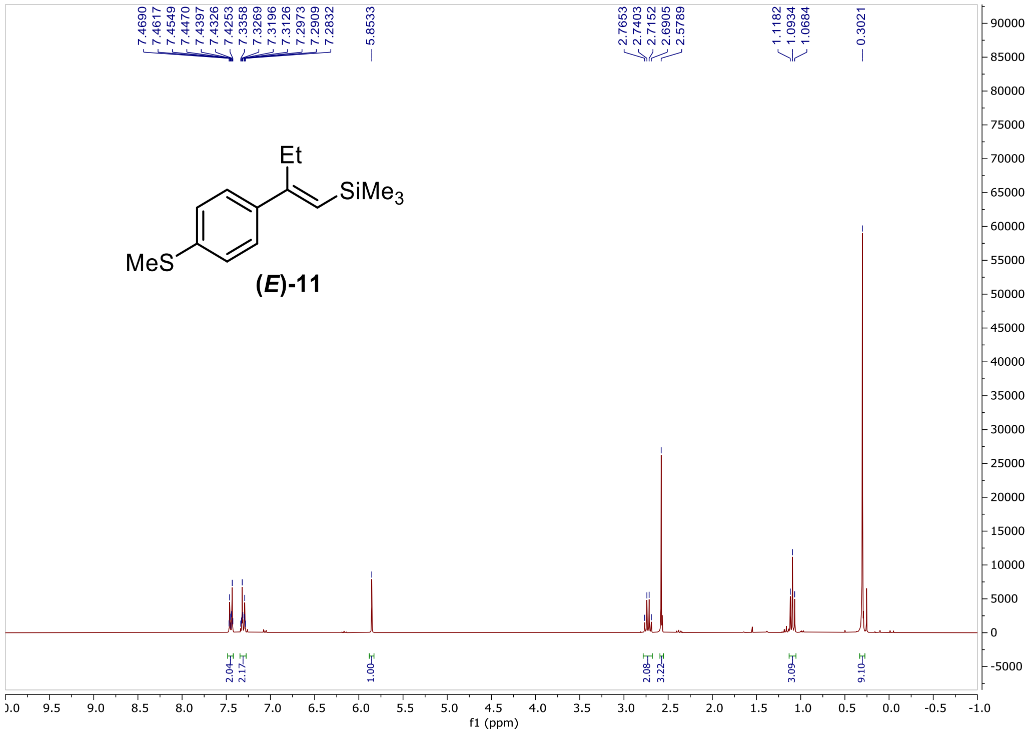

^{1}H NMR (75 MHz, $CDCl_3$)

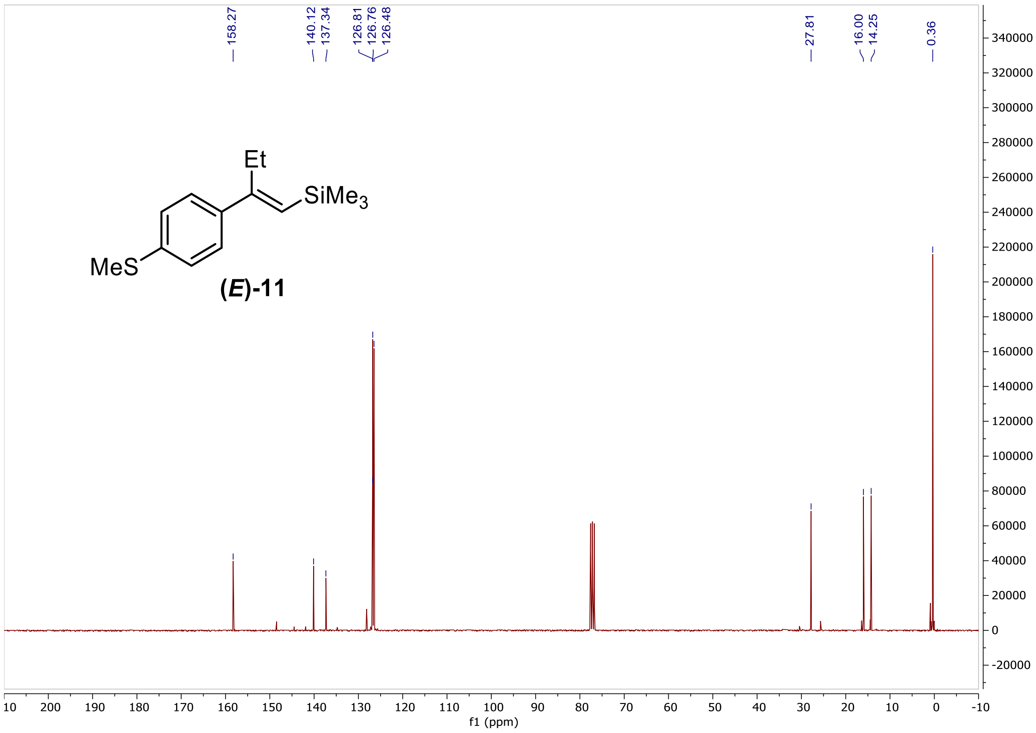

(*E*)-trimethyl(2-(4-(trifluoromethoxy)phenyl)but-1-en-1-yl)silane ((*E*)-12)

^{1}H NMR (300 MHz, $CDCl_3$)

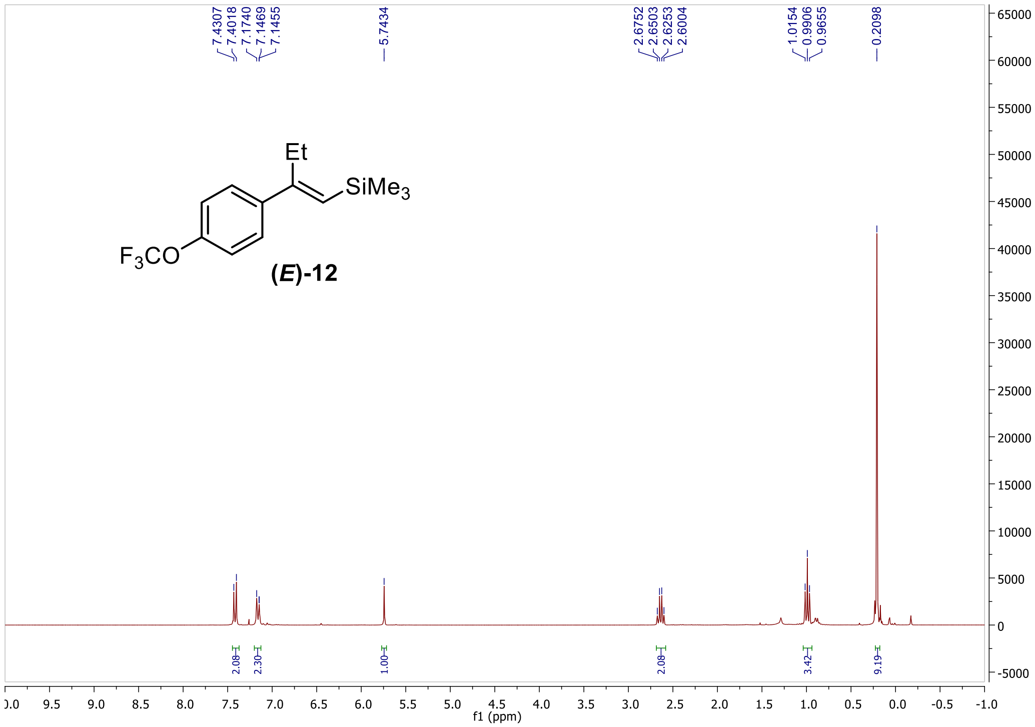

^{13}C NMR (75 MHz, $CDCl_3$)

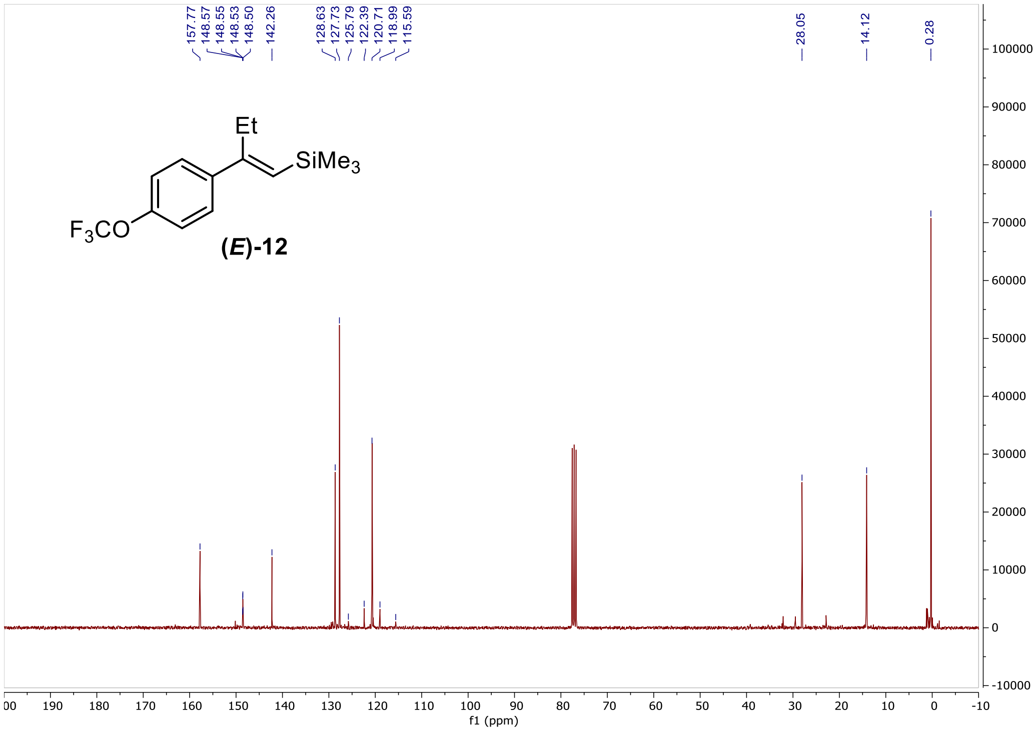

(*E*)-trimethyl(2-(4-(4,4,5,5-tetramethyl-1,3,2-dioxaborolan-2-yl)phenyl)but-1-en-1-yl)silane ((*E*)-13)

^{1}H NMR (300 MHz, $CDCl_3$)

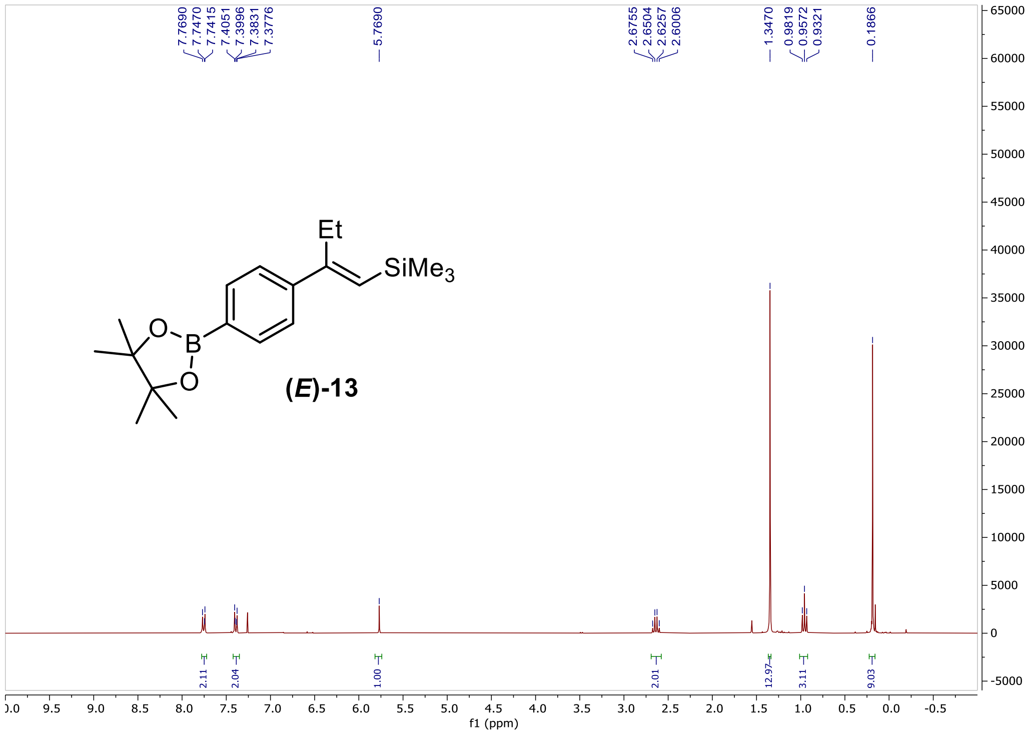

^{13}C NMR (75 MHz, $CDCl_3$)

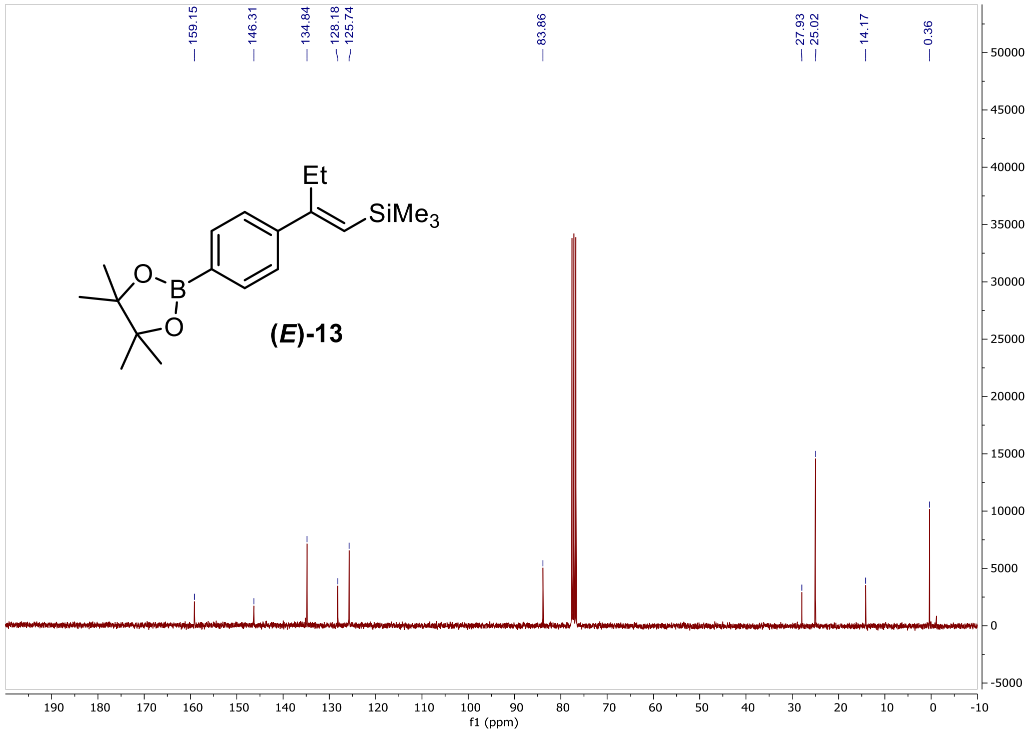

(*E*)-(2-(4-fluorophenyl)but-1-en-1-yl)trimethylsilane ((*E*)-14)

^{1}H NMR (300 MHz, $CDCl_3$)

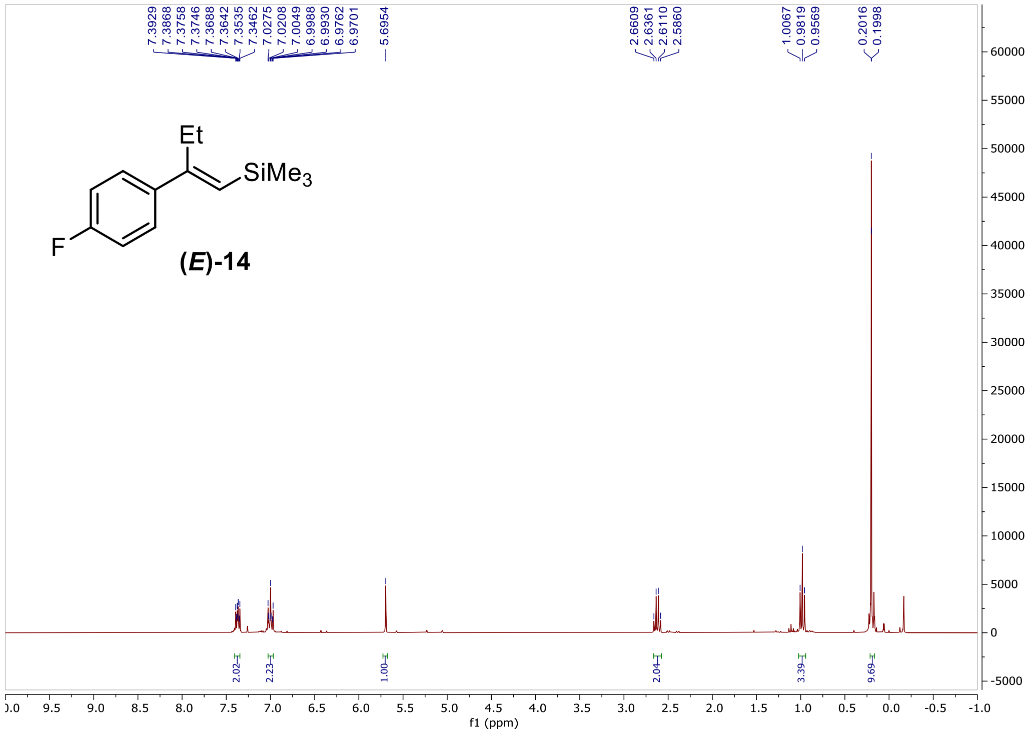

^{13}C NMR (75 MHz, $CDCl_3$)

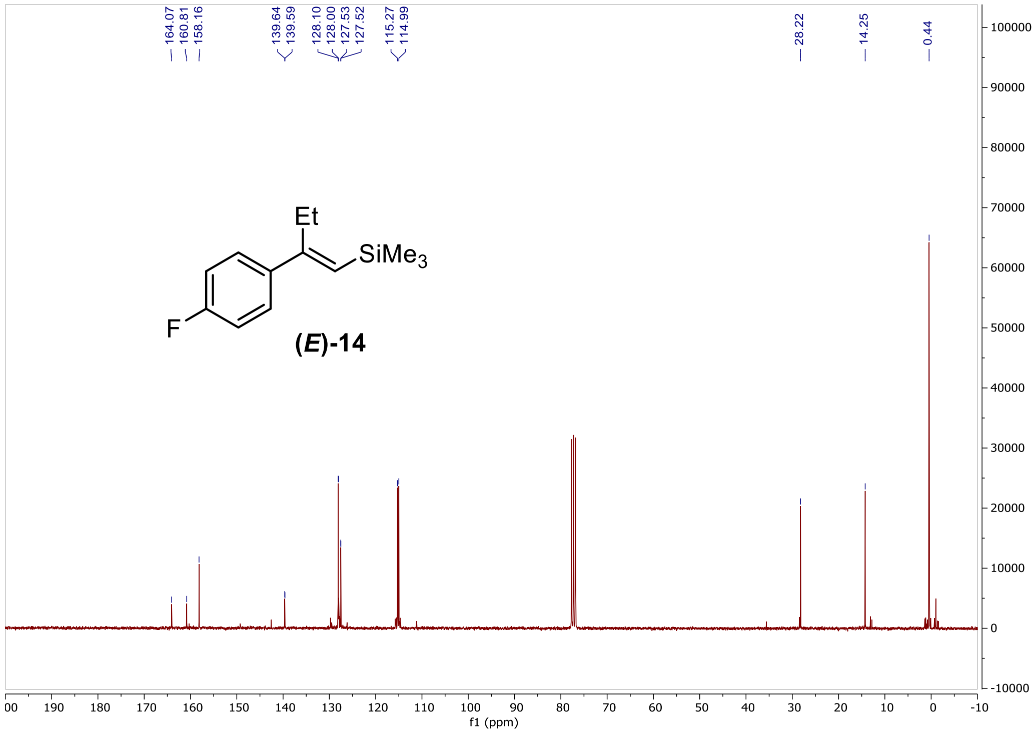

(*E*)-trimethyl(2-(4-(trifluoromethyl)phenyl)but-1-en-1-yl)silane ((*E*)-15)

^{1}H NMR (300 MHz, $CDCl_3$)

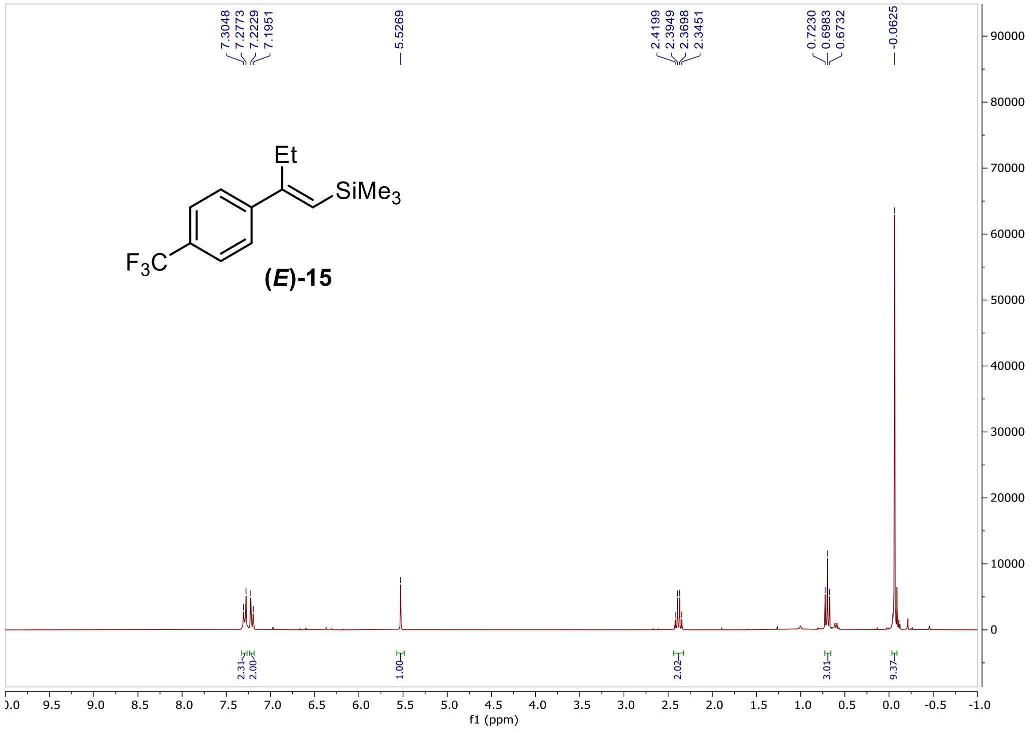

^{13}C NMR (75 MHz, $CDCl_3$)

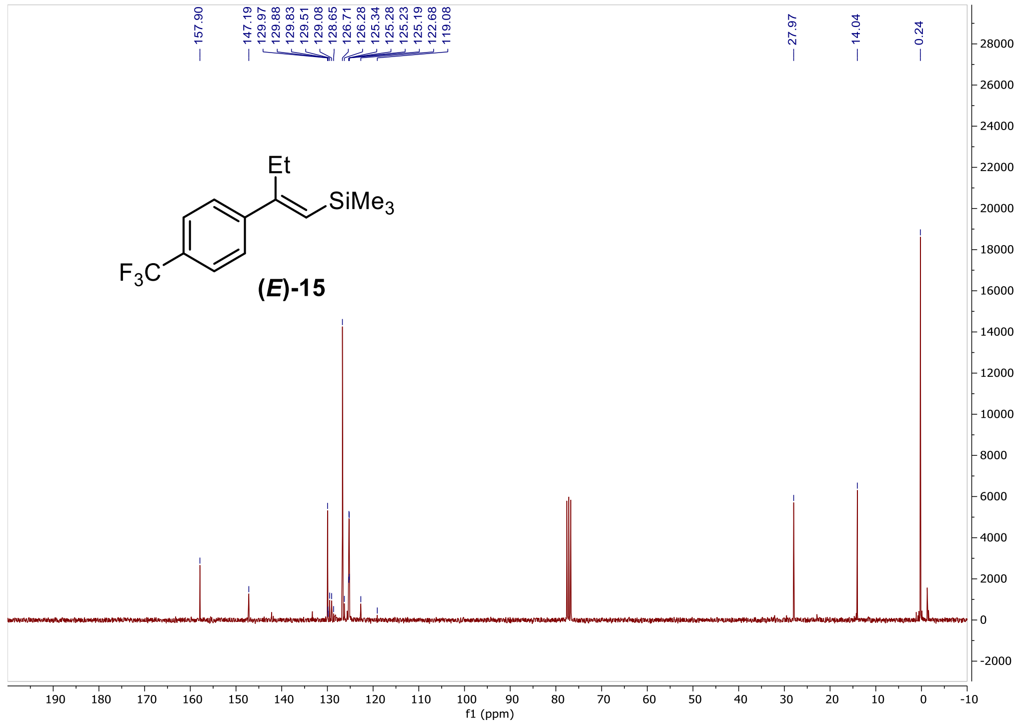

(*E*)-(2-(4-bromophenyl)but-1-en-1-yl)trimethylsilane ((*E*)-16)

^{1}H NMR (300 MHz, $CDCl_3$)

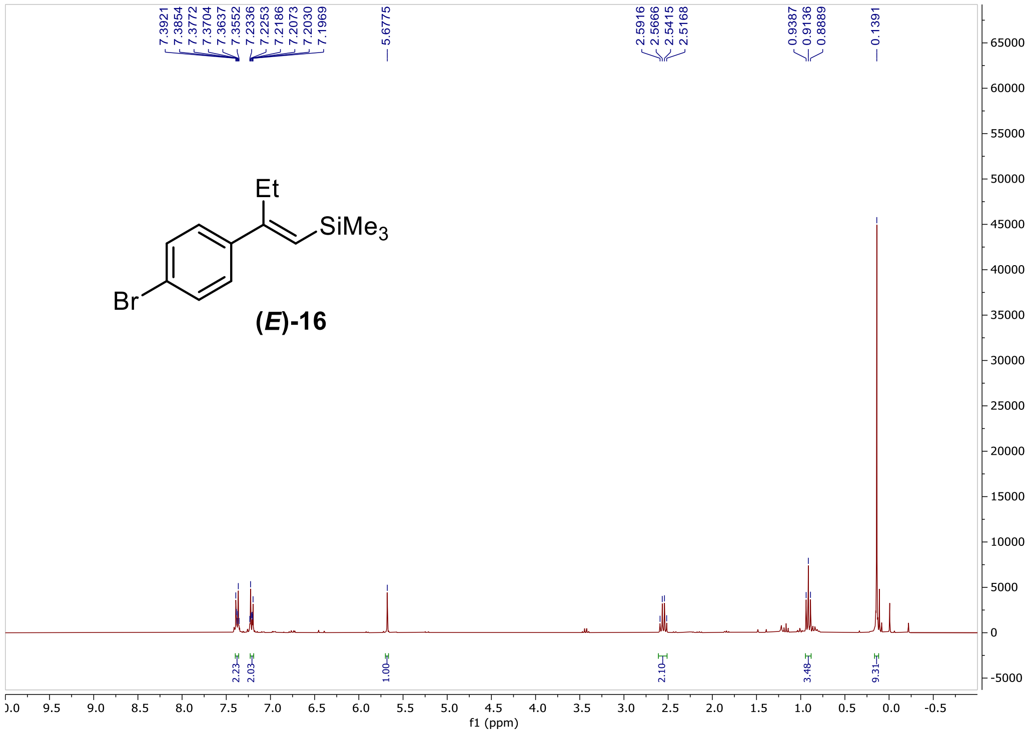

^{13}C NMR (75 MHz, $CDCl_3$)

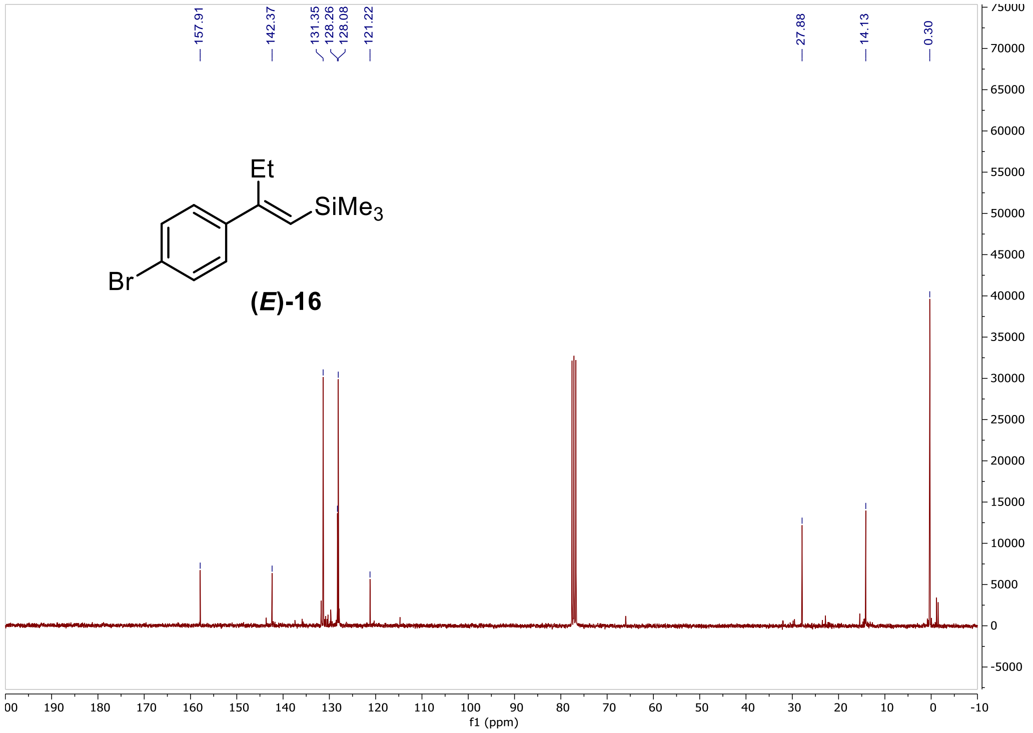

(*E*)-trimethyl(2-(4-(methylsulfonyl)phenyl)but-1-en-1-yl)silane ((*E*)-17)

^{1}H NMR (300 MHz, $CDCl_3$)

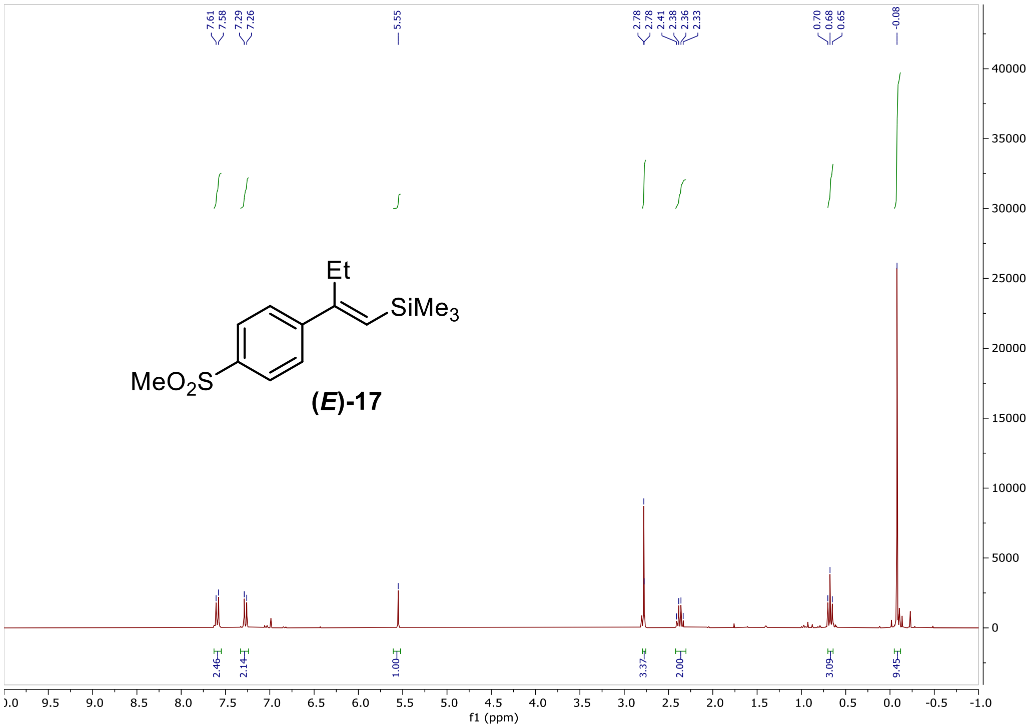

^{13}C NMR (75 MHz, $CDCl_3$)

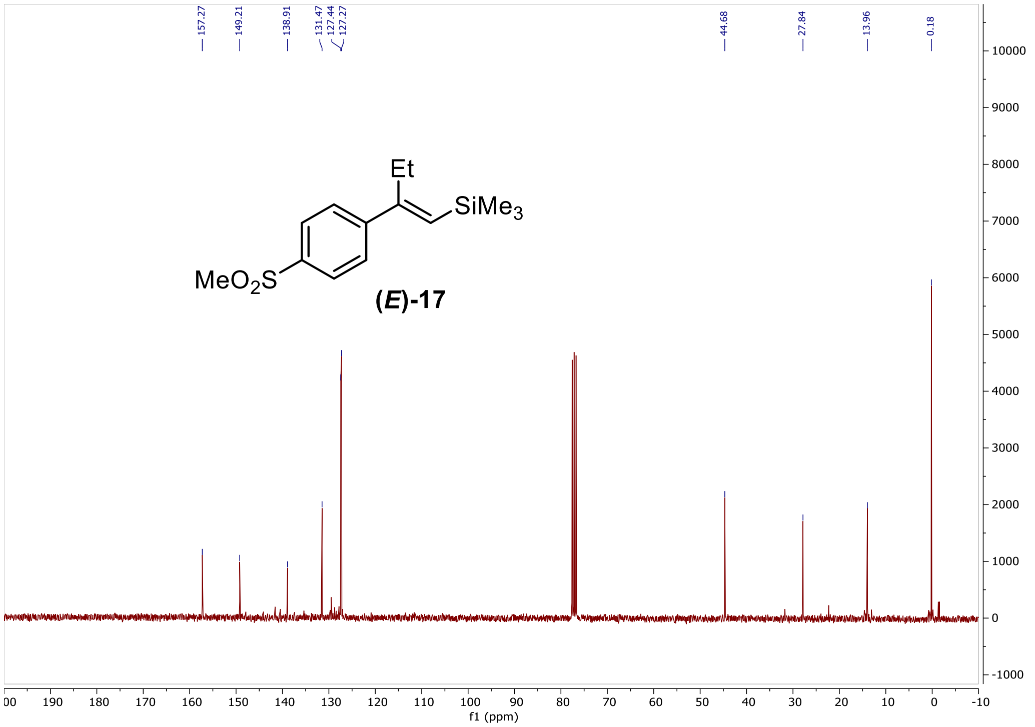

(*E*)-trimethyl(2-(naphthalen-2-yl)but-1-en-1-yl)silane ((*E*)-18)

^{1}H NMR (300 MHz, $CDCl_3$)

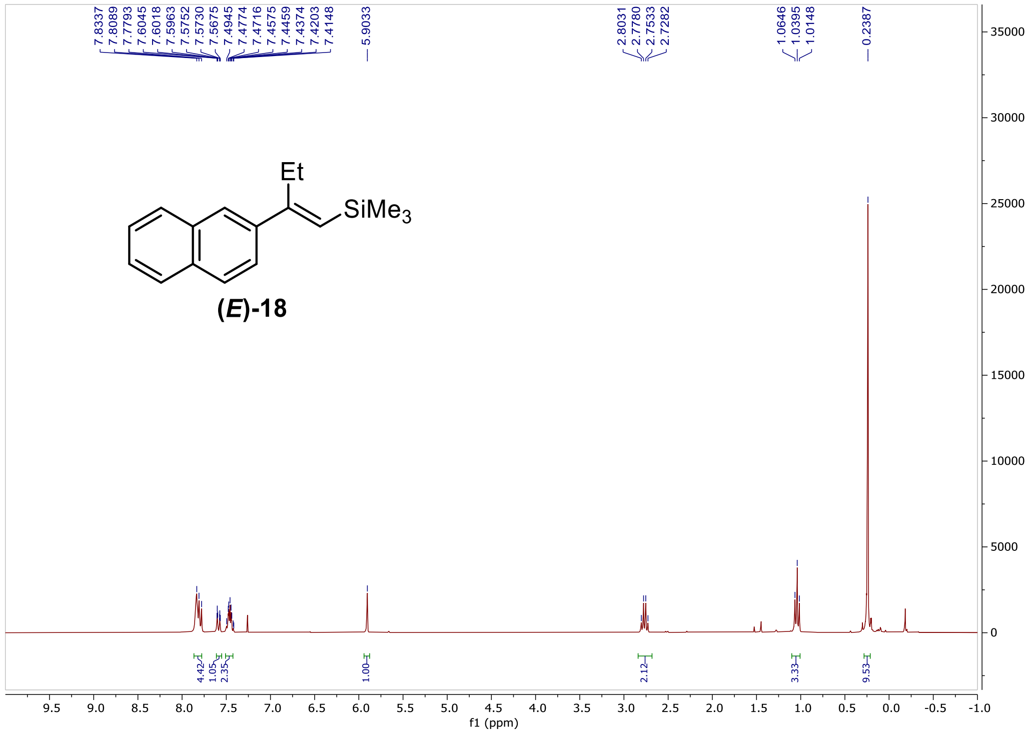

^{13}C NMR (75 MHz, $CDCl_3$)

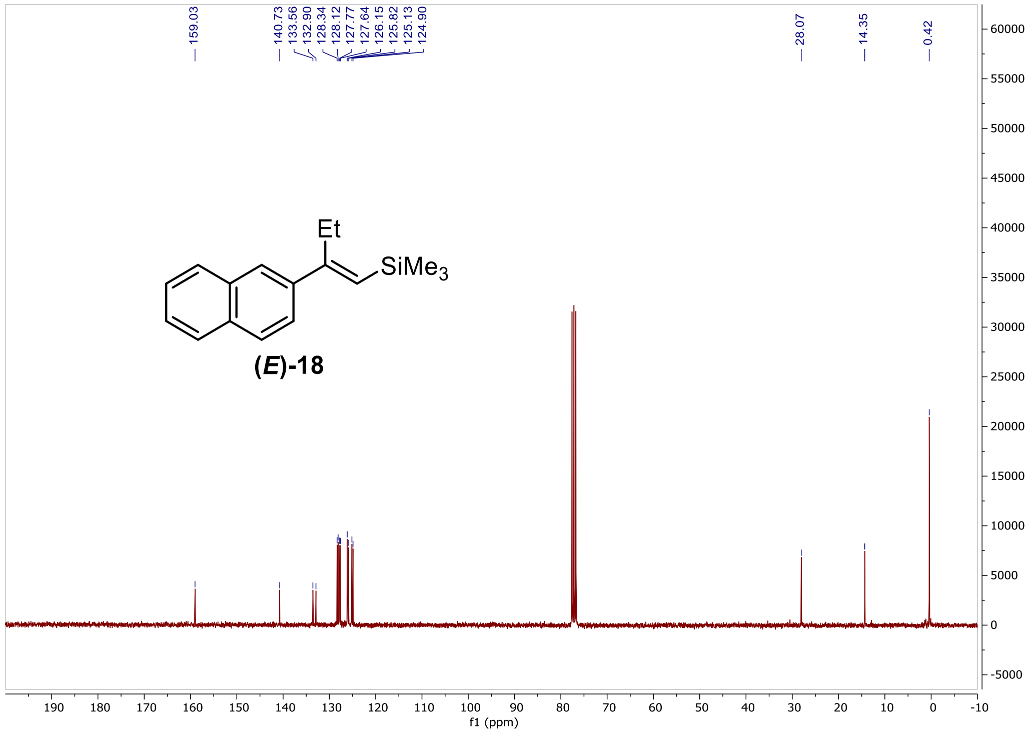

(*Z*)-isomers

(*Z*)-trimethyl(2-phenylbut-1-en-1-yl)silane ((*Z*)-1)

^{1}H NMR (300 MHz, $CDCl_3$)

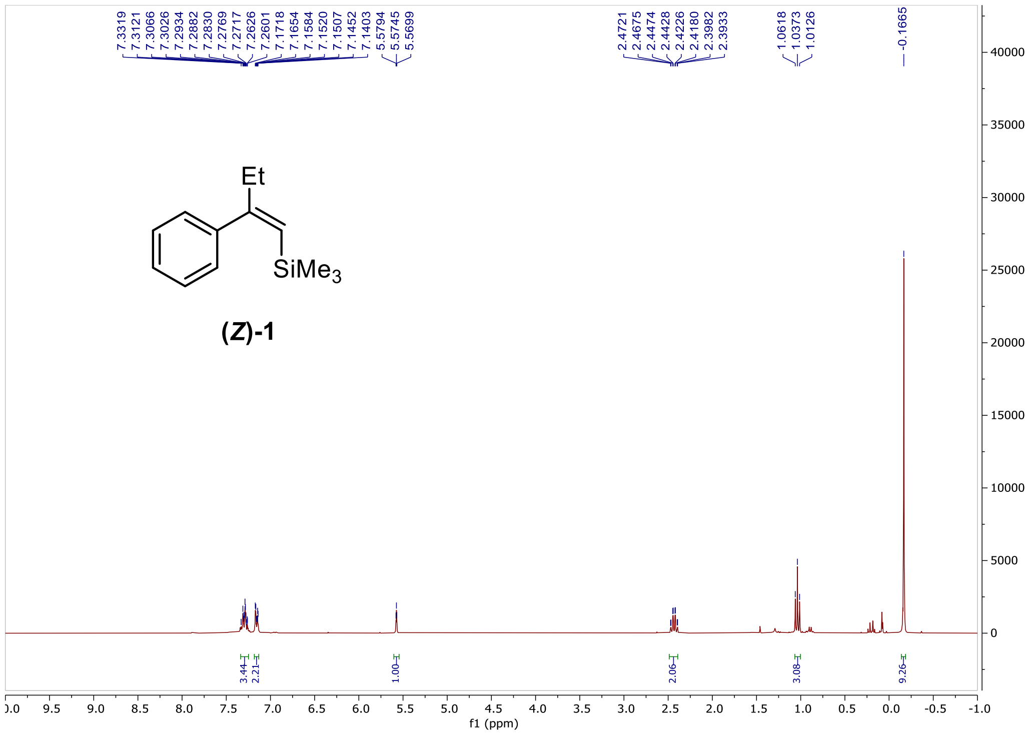

^{13}C NMR (75 MHz, $CDCl_3$)

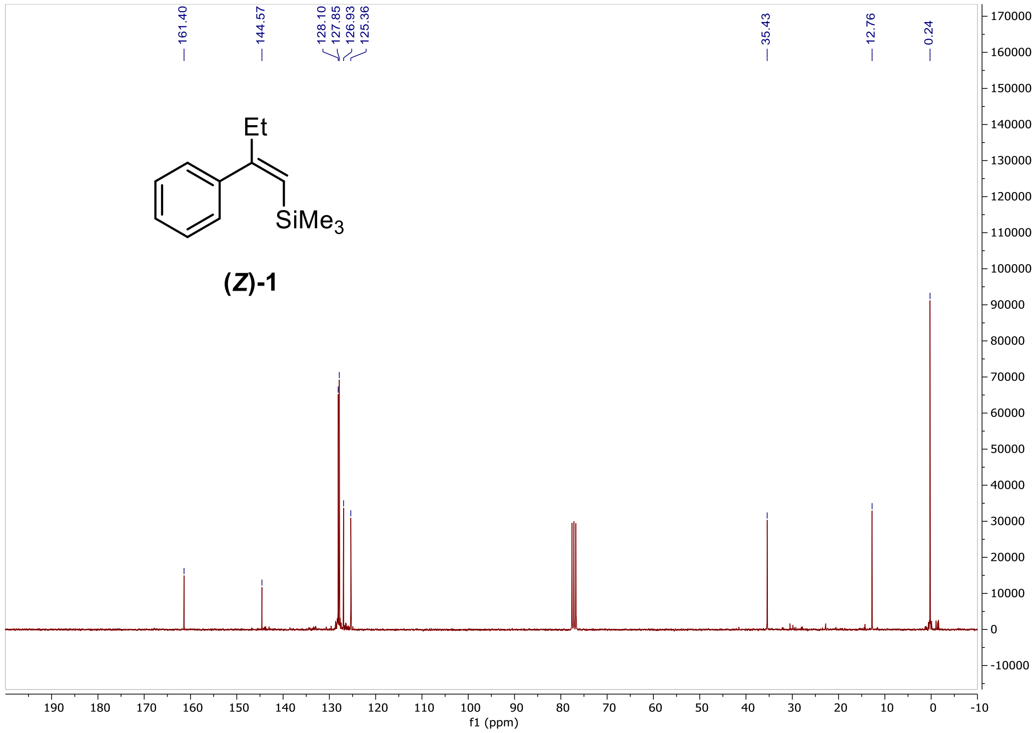

(*Z*)-trimethyl(2-phenylprop-1-en-1-yl)silane ((*Z*)-2)

^{1}H NMR (300 MHz, $CDCl_3$)

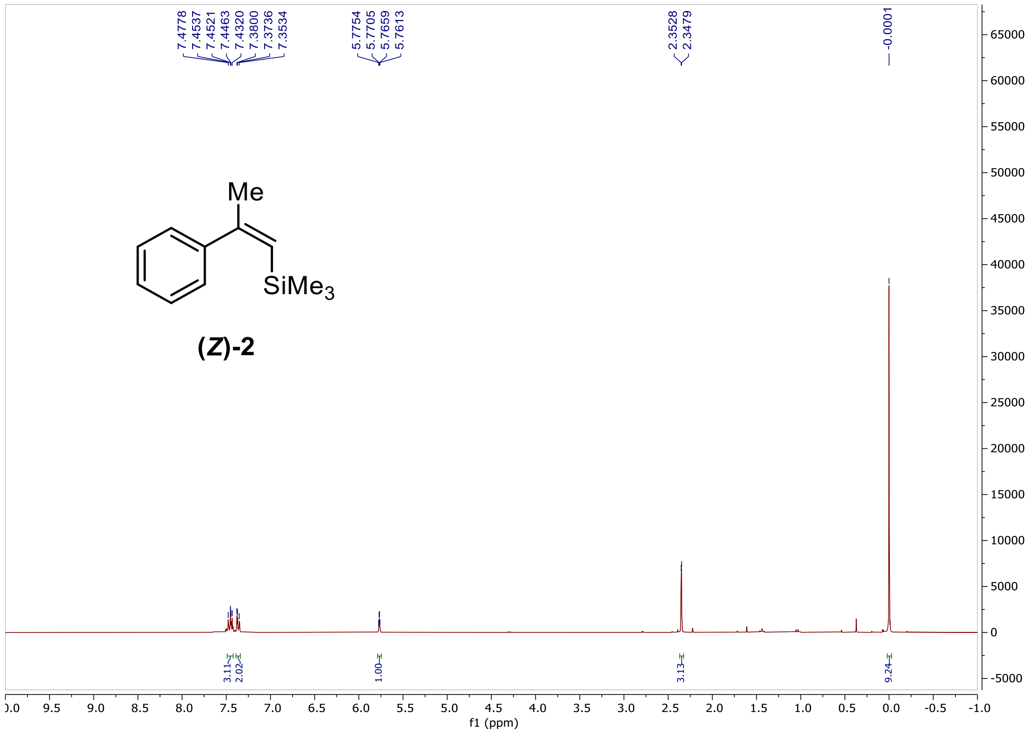

^{13}C NMR (75 MHz, $CDCl_3$)

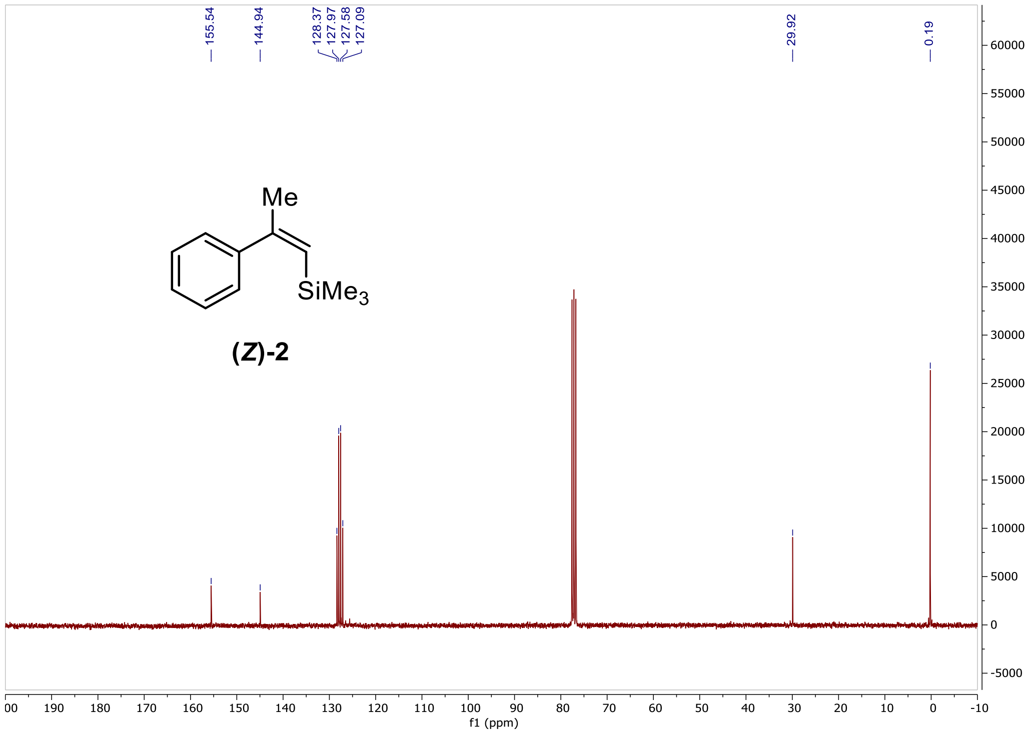

(*Z*)-trimethyl(2-phenylpenta-1,4-dien-1-yl)silane ((*Z*)-3)

^{1}H NMR (300 MHz, $CDCl_3$)

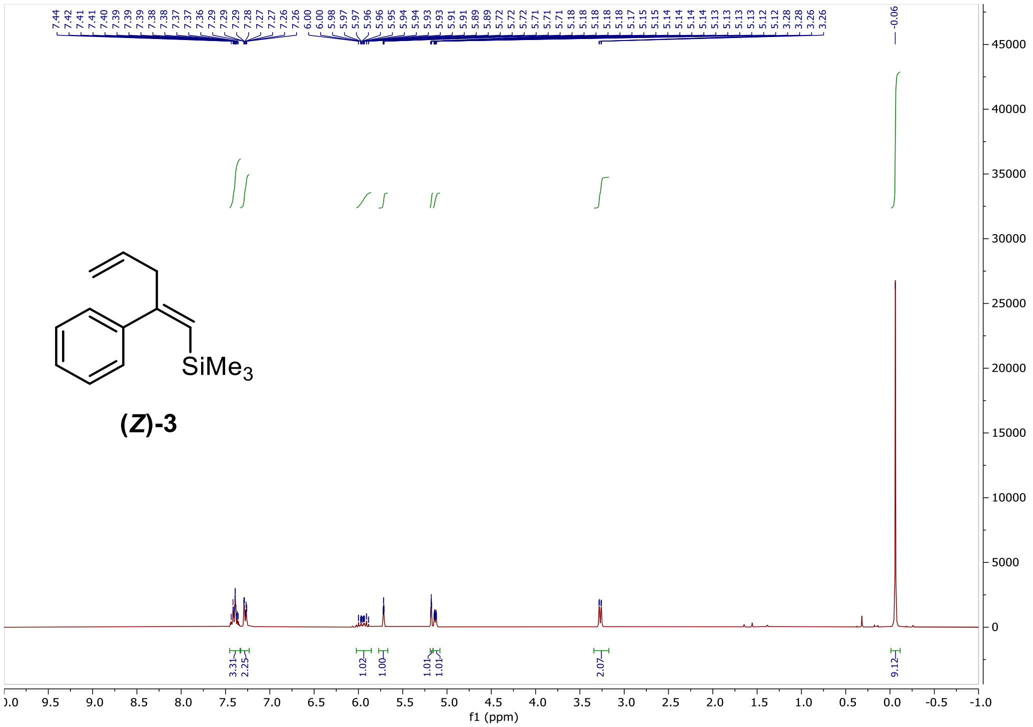

^{13}C NMR (75 MHz, $CDCl_3$)

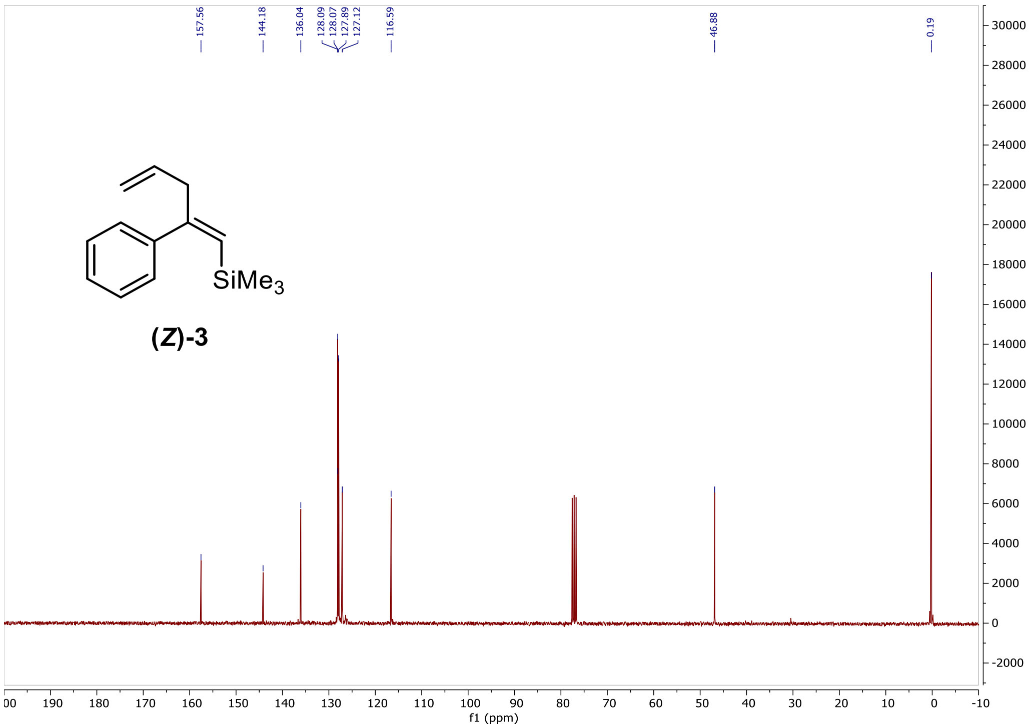

(*E*)-trimethyl(2-phenyl-2-(trimethylstannyl)vinyl)silane ((*E*)-4)

^{1}H NMR (300 MHz, $CDCl_3$)

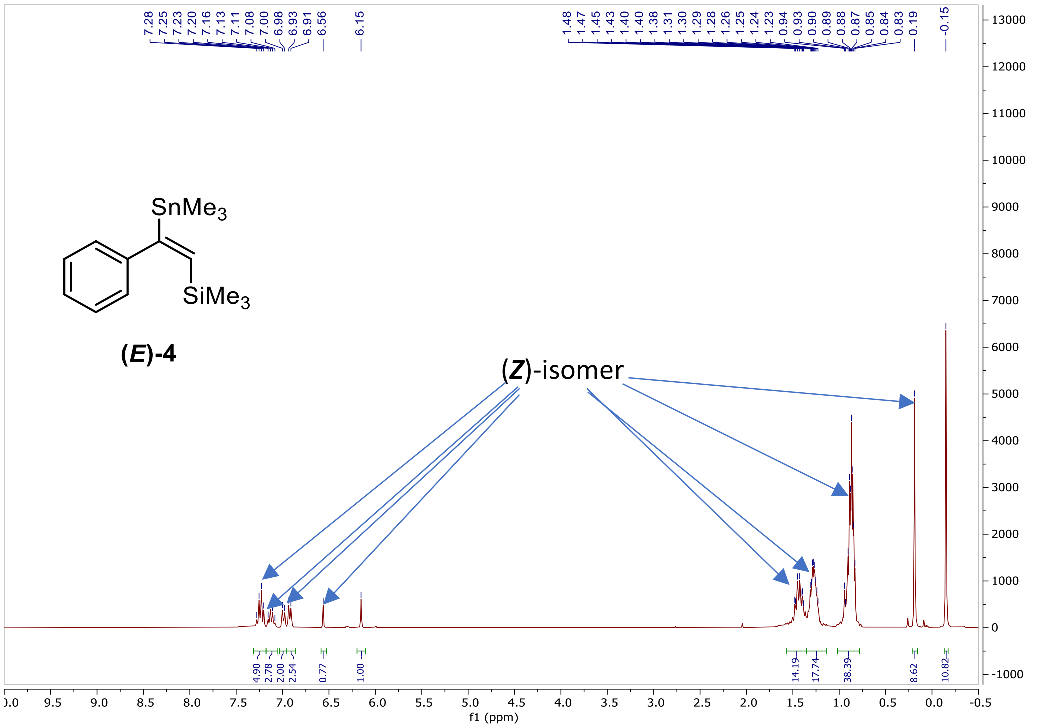

^{13}C NMR (75 MHz, $CDCl_3$)

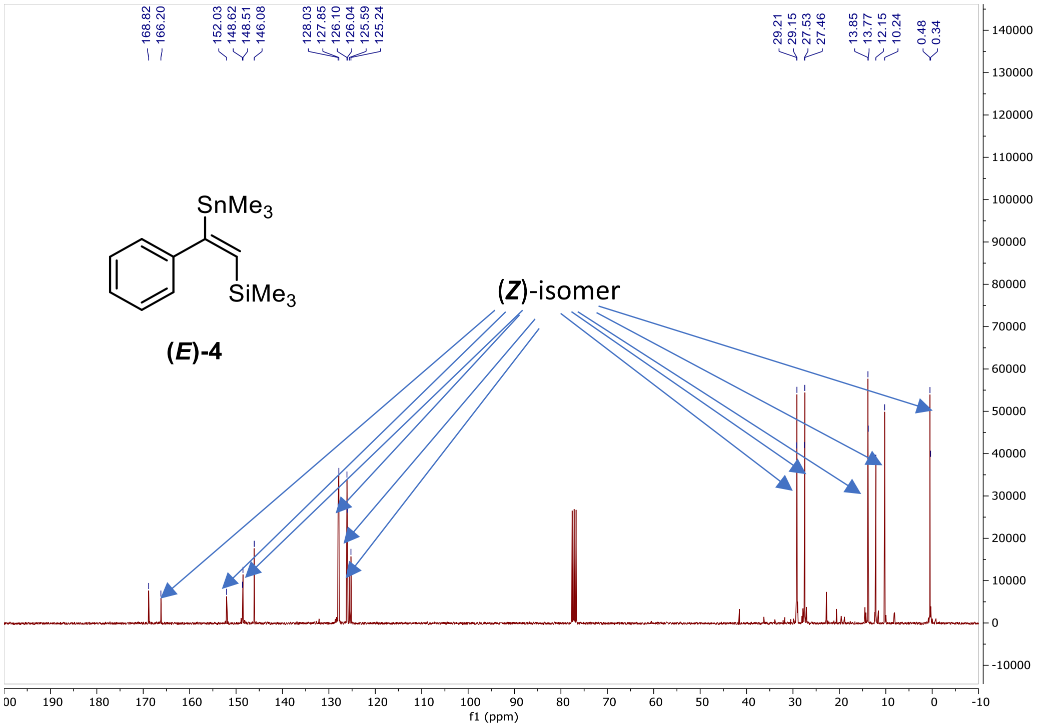

(*Z*)-tert-butyldimethyl(styryl)silane ((*Z*)-5)

^{1}H NMR (300 MHz, $CDCl_3$)

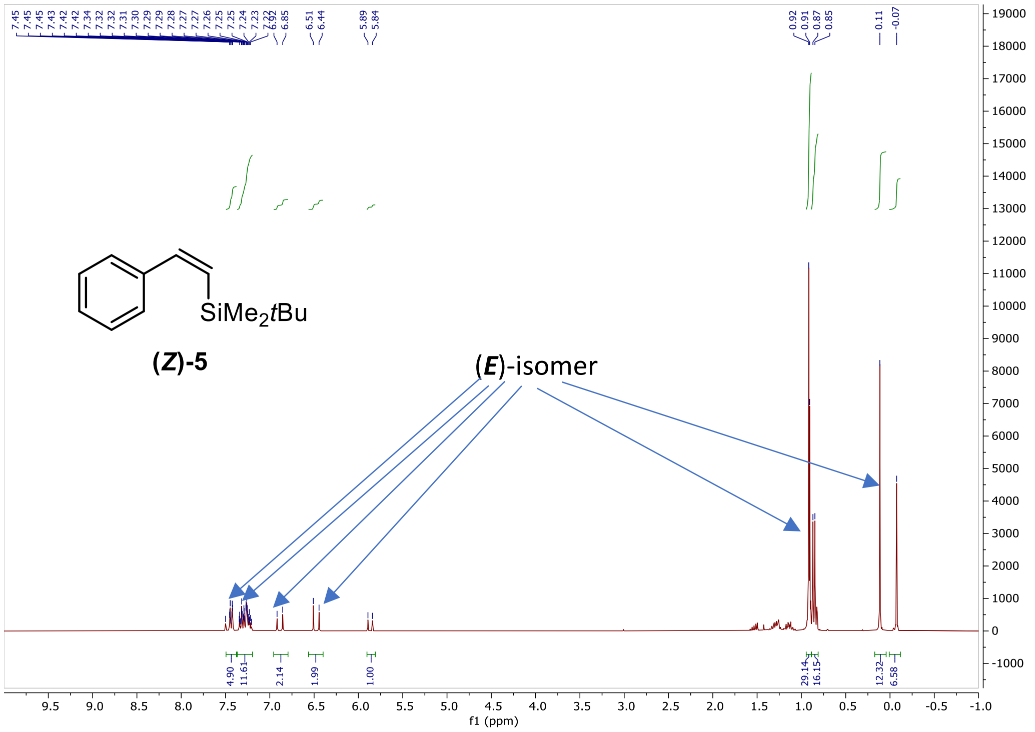

(*Z*)-dimethyl(2-phenylbut-1-en-1-yl)silanol ((*Z*)-6)

^{1}H NMR (300 MHz, $CDCl_3$)

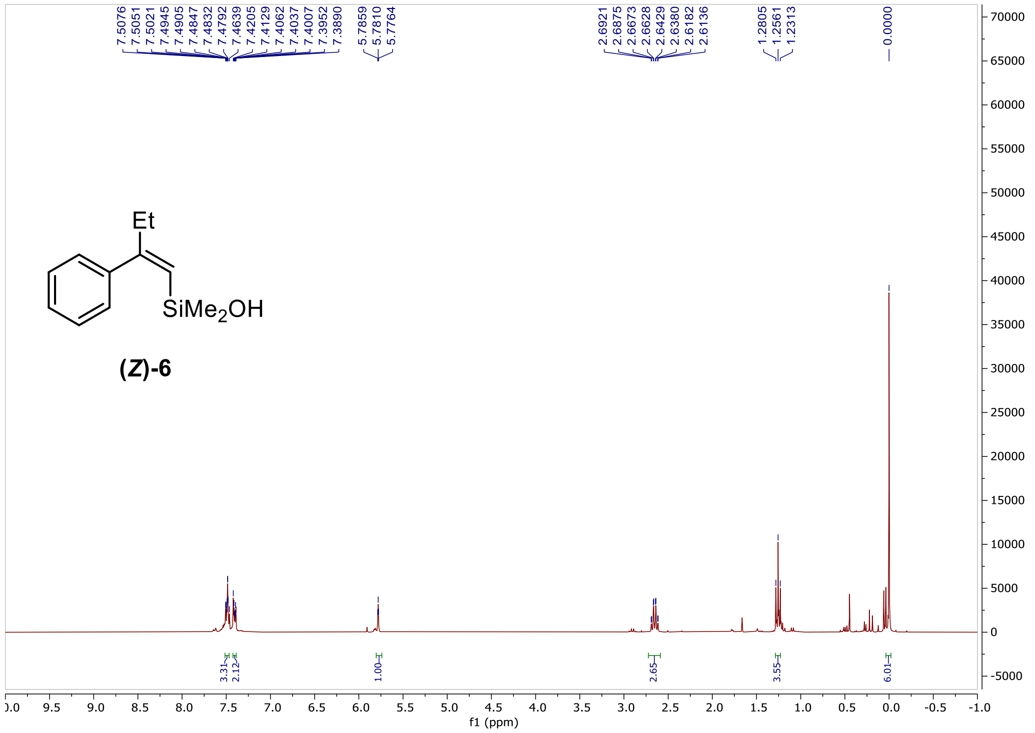

^{13}C NMR (75 MHz, $CDCl_3$)

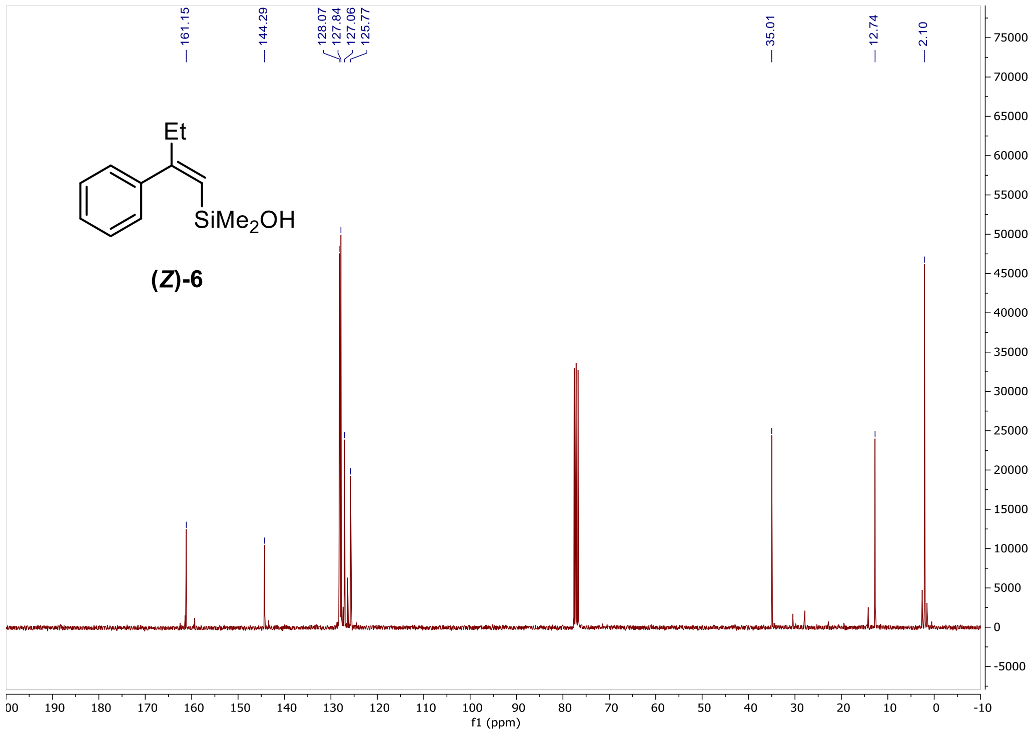

(*Z*)-trimethyl(2-(p-tolyl)but-1-en-1-yl)silane ((*Z*)-7)

^{1}H NMR (300 MHz, $CDCl_3$)

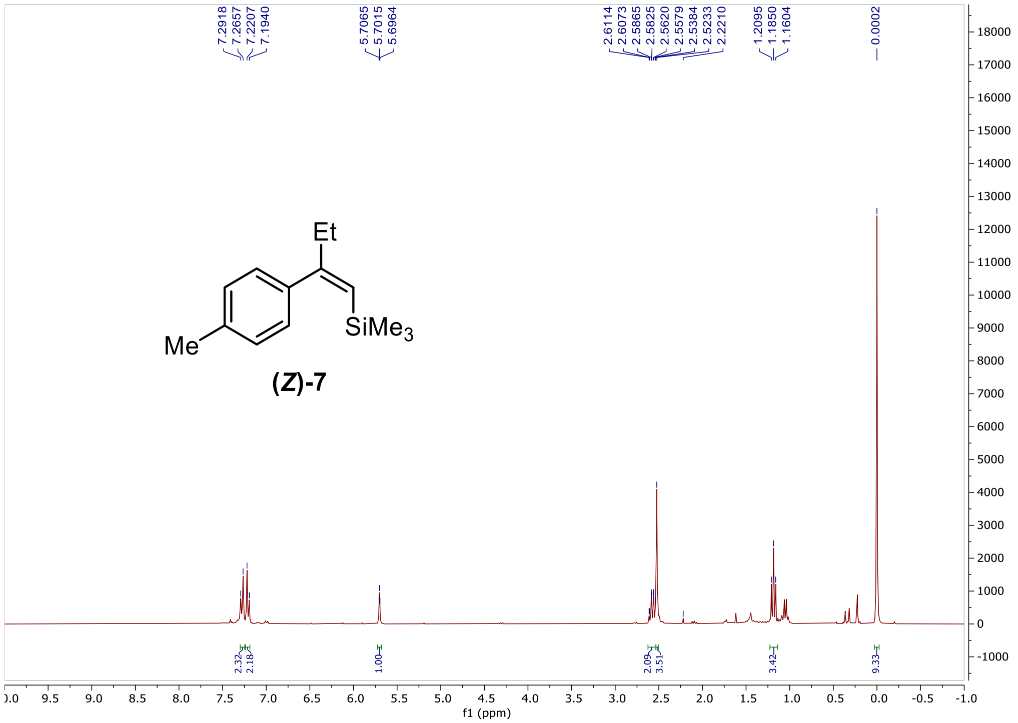

^{13}C NMR (75 MHz, $CDCl_3$)

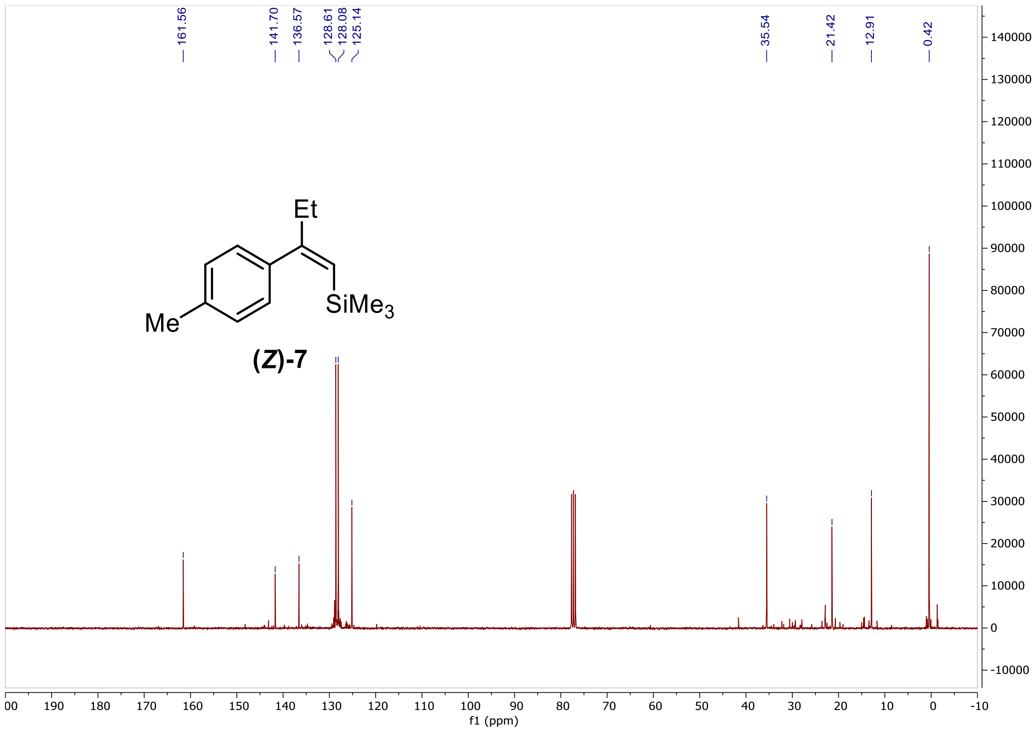

(*Z*)-(2-(4-(tert-butyl)phenyl)but-1-en-1-yl)trimethylsilane ((*Z*)-8)

^{1}H NMR (300 MHz, $CDCl_3$)

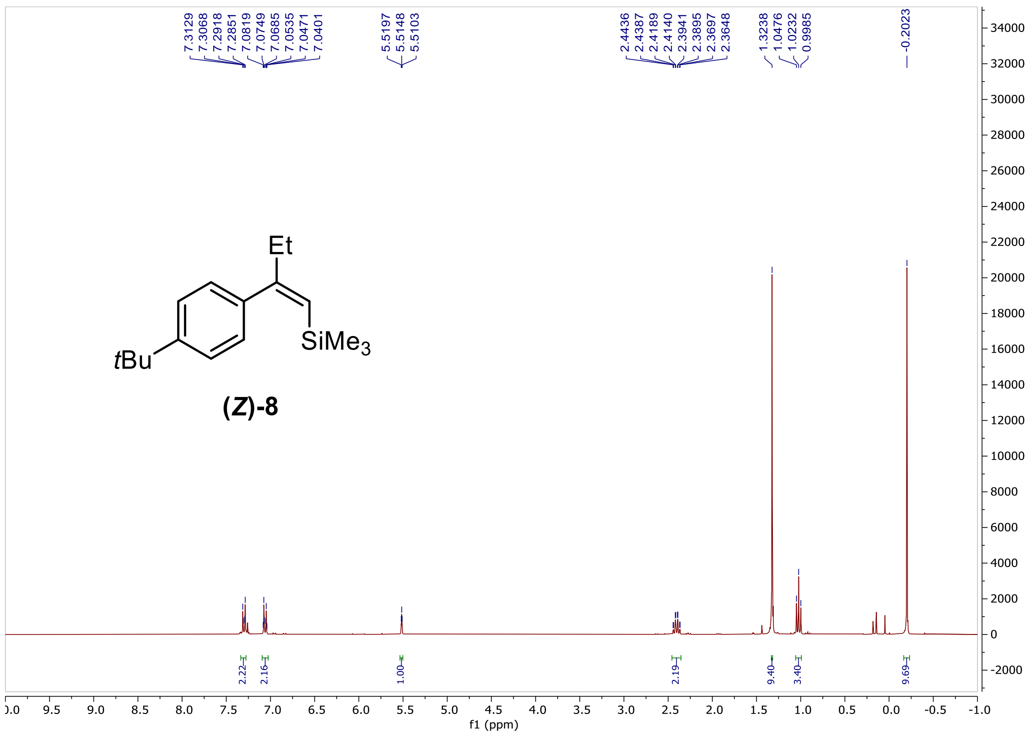

^{13}C NMR (75 MHz, $CDCl_3$)

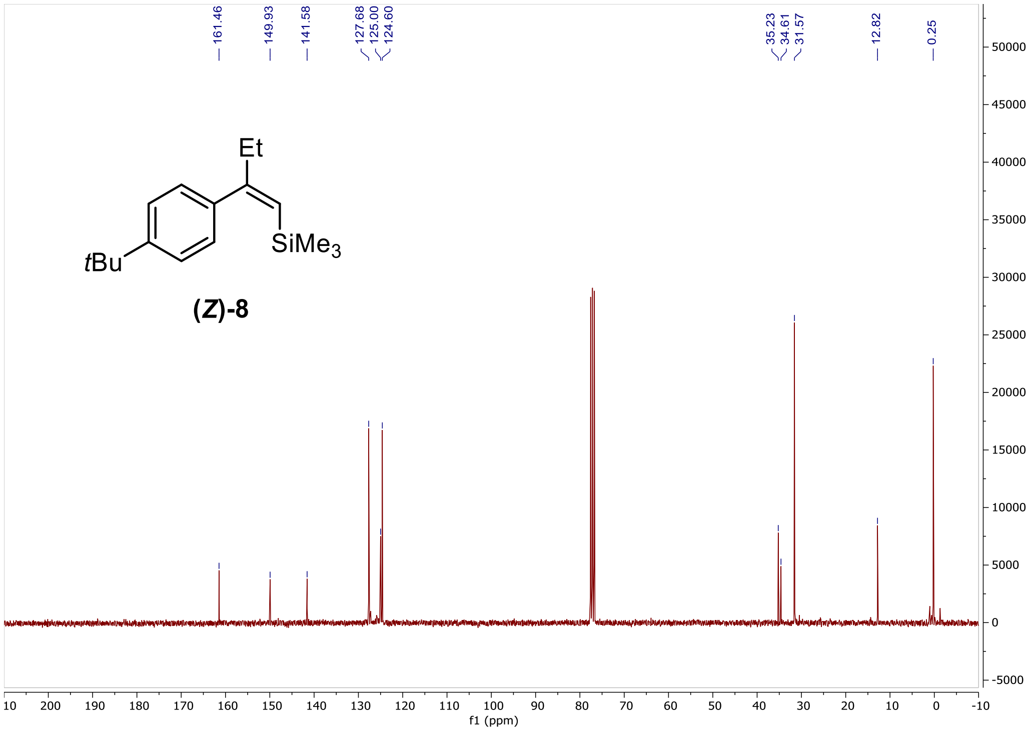

(*Z*)-trimethyl(2-(m-tolyl)but-1-en-1-yl)silane ((*Z*)-9)

^{1}H NMR (300 MHz, $CDCl_3$)

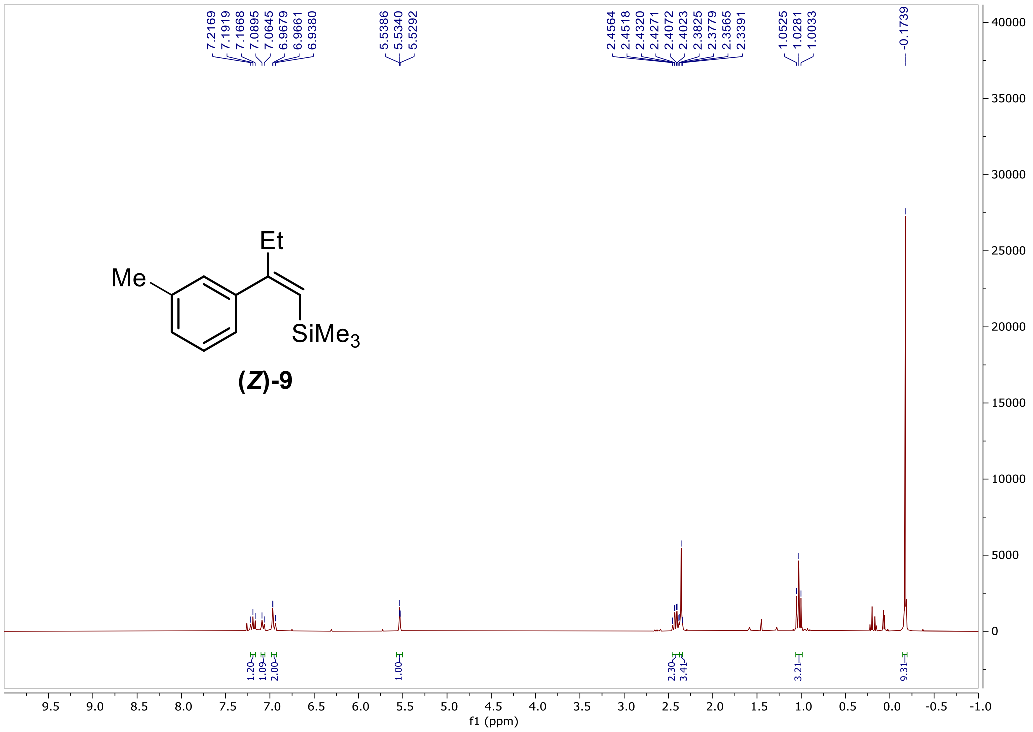

^{13}C NMR (75 MHz, CDCl$_3$)

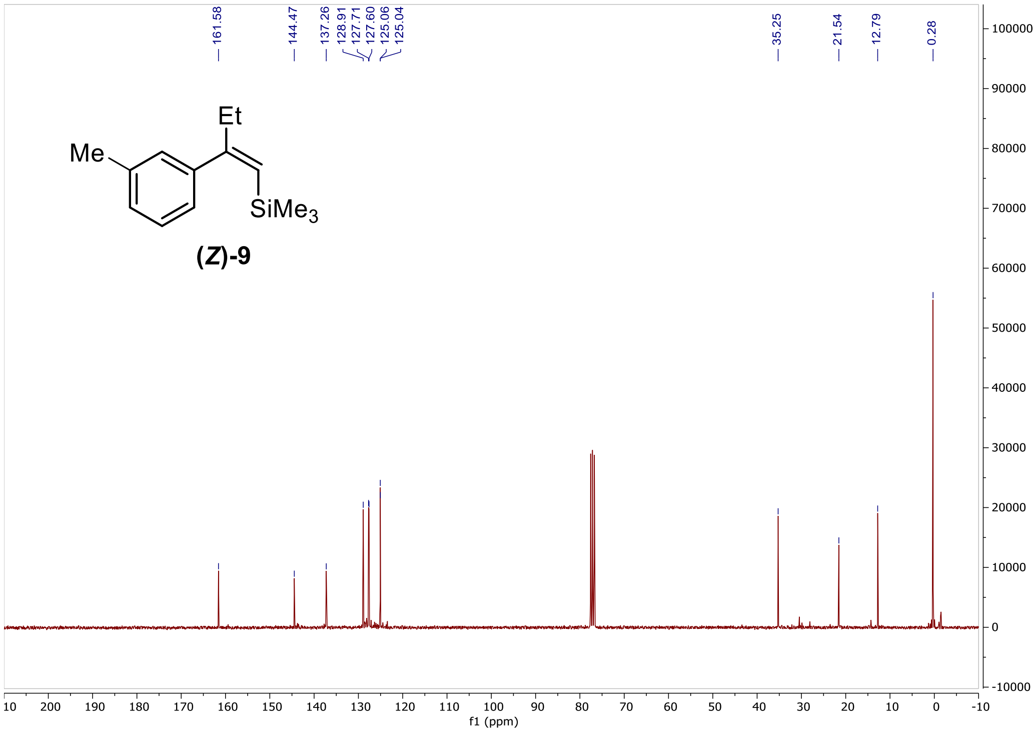

(*Z*)-(2-([1,1'-biphenyl]-4-yl)but-1-en-1-yl)trimethylsilane ((*Z*)-10)

^{1}H NMR (300 MHz, CDCl$_3$)

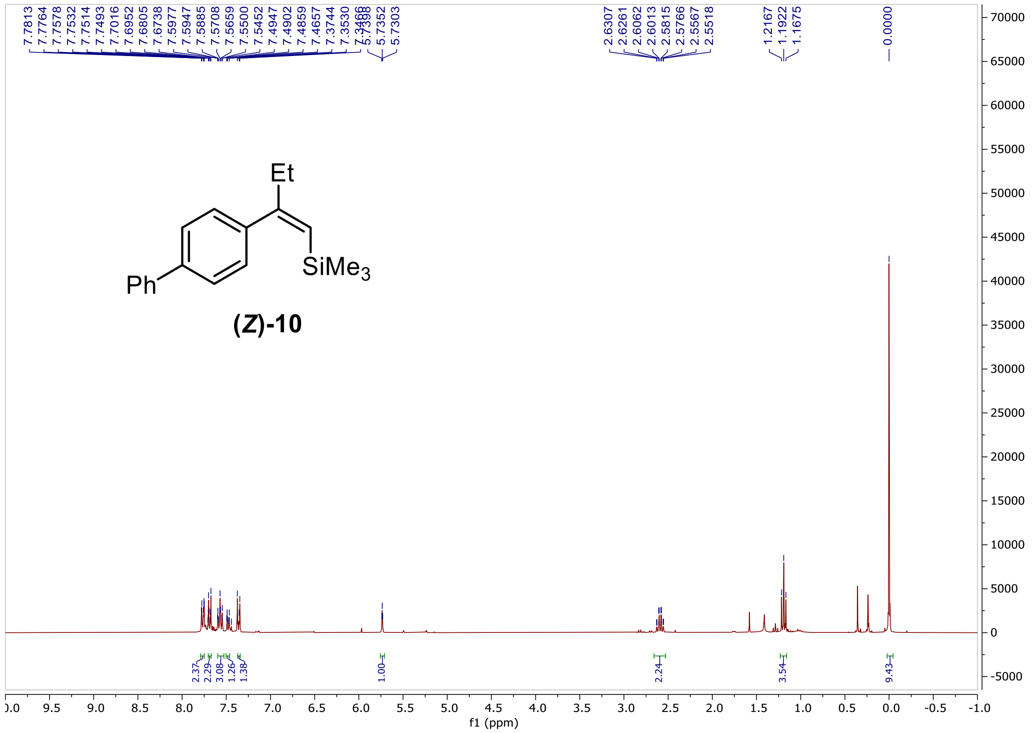

^{13}C NMR (75 MHz, $CDCl_3$)

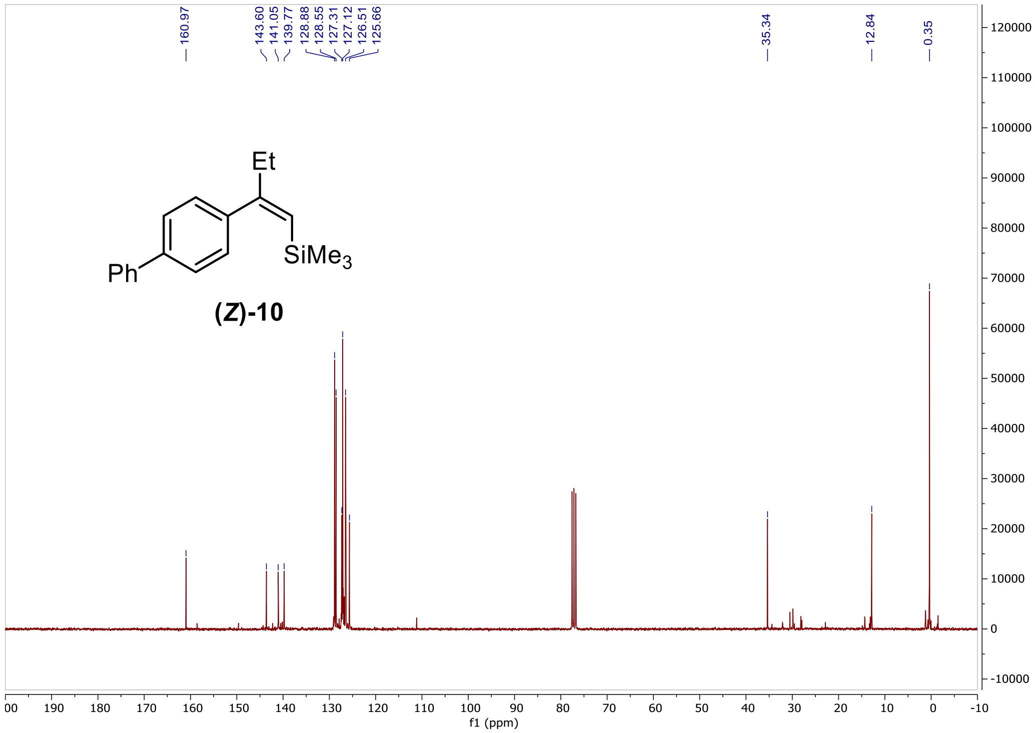

(*Z*)-trimethyl(2-(4-(methylthio)phenyl)but-1-en-1-yl)silane ((*Z*)-11)

^{1}H NMR (300 MHz, $CDCl_3$)

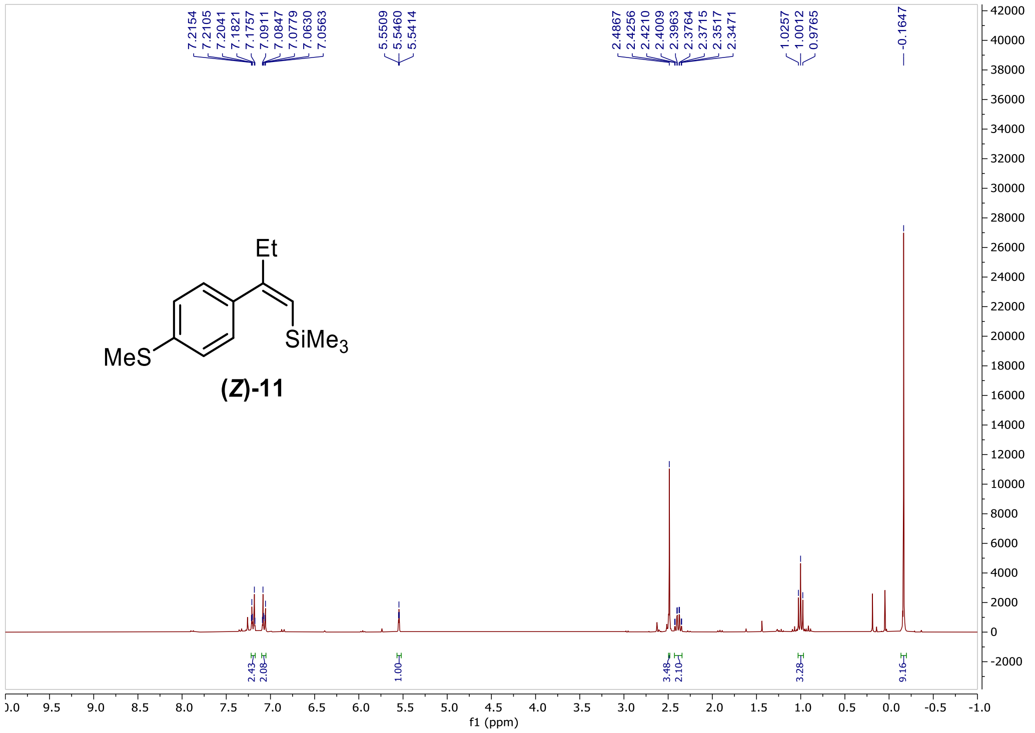

^{13}C NMR (75 MHz, $CDCl_3$)

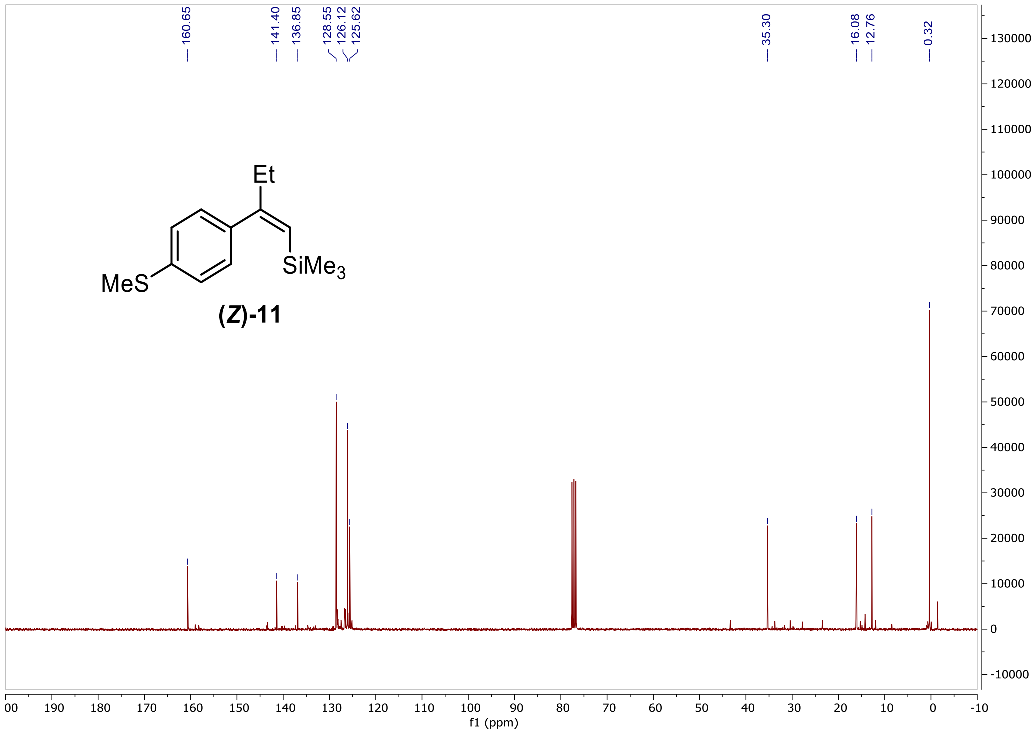

(*Z*)-trimethyl(2-(4-(trifluoromethoxy)phenyl)but-1-en-1-yl)silane ((*Z*)-12)

^{1}H NMR (300 MHz, $CDCl_3$)

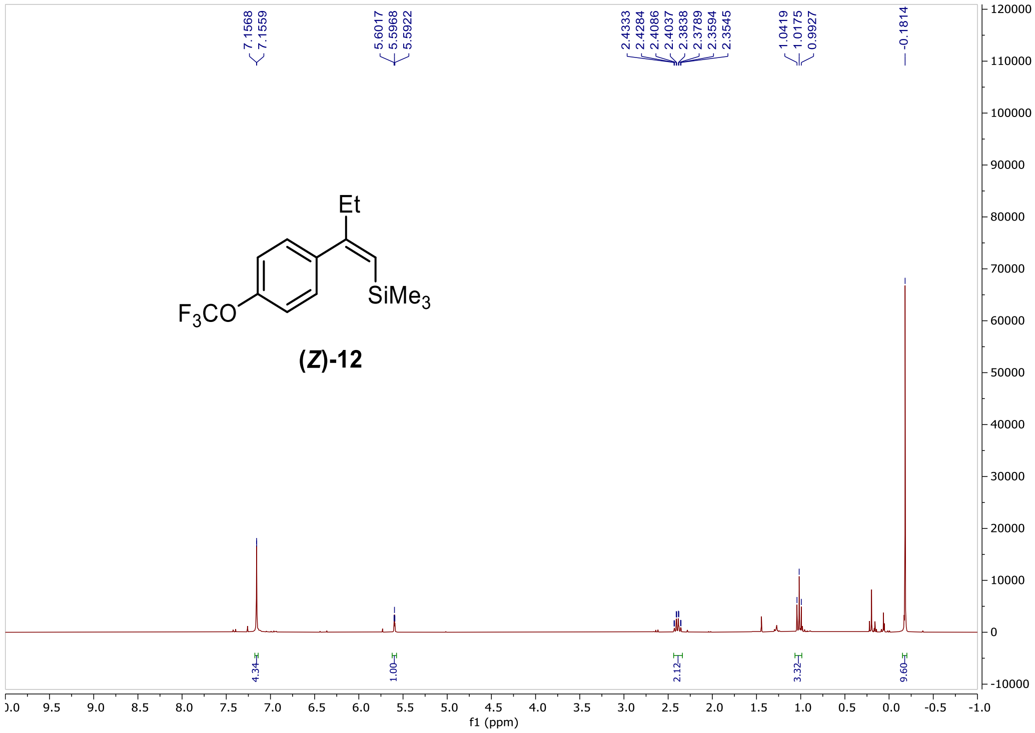

^{13}C NMR (75 MHz, $CDCl_3$)

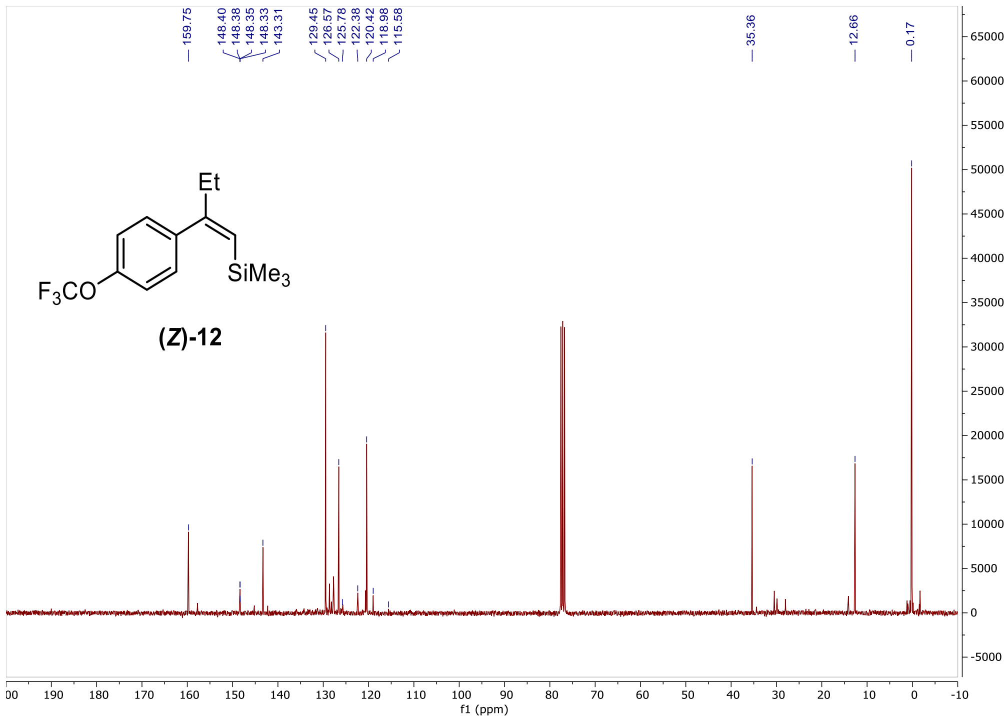

(*Z*)-trimethyl(2-(4-(4,4,5,5-tetramethyl-1,3,2-dioxaborolan-2-yl)phenyl)but-1-en-1-yl)silane ((*Z*)-13)

^{1}H NMR (300 MHz, $CDCl_3$)

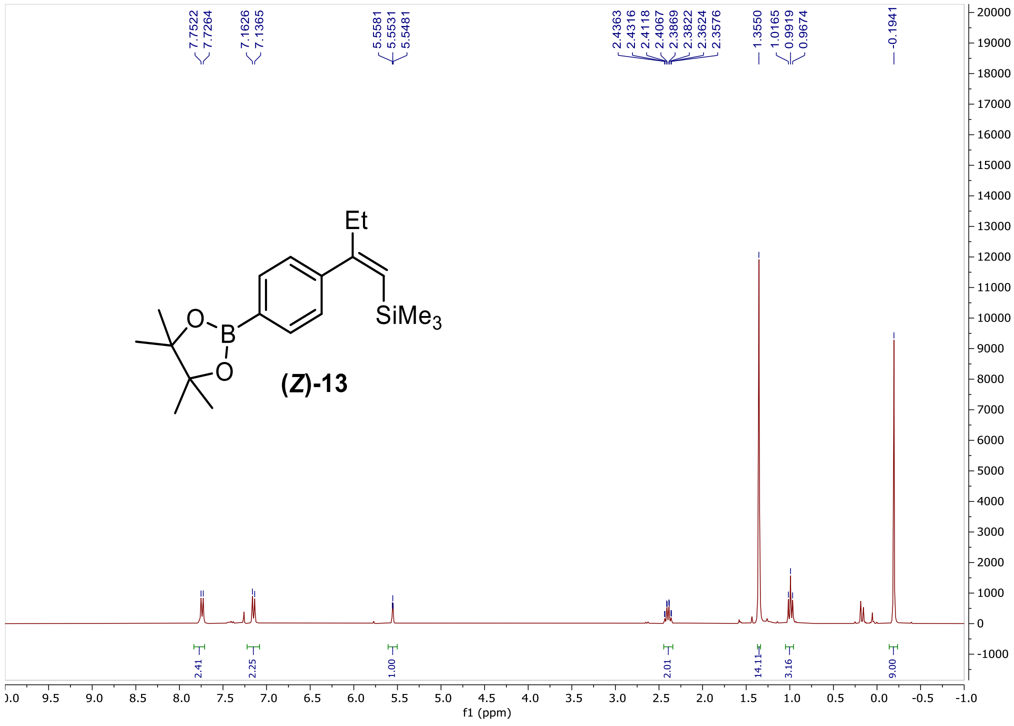

^{13}C NMR (75 MHz, $CDCl_3$)

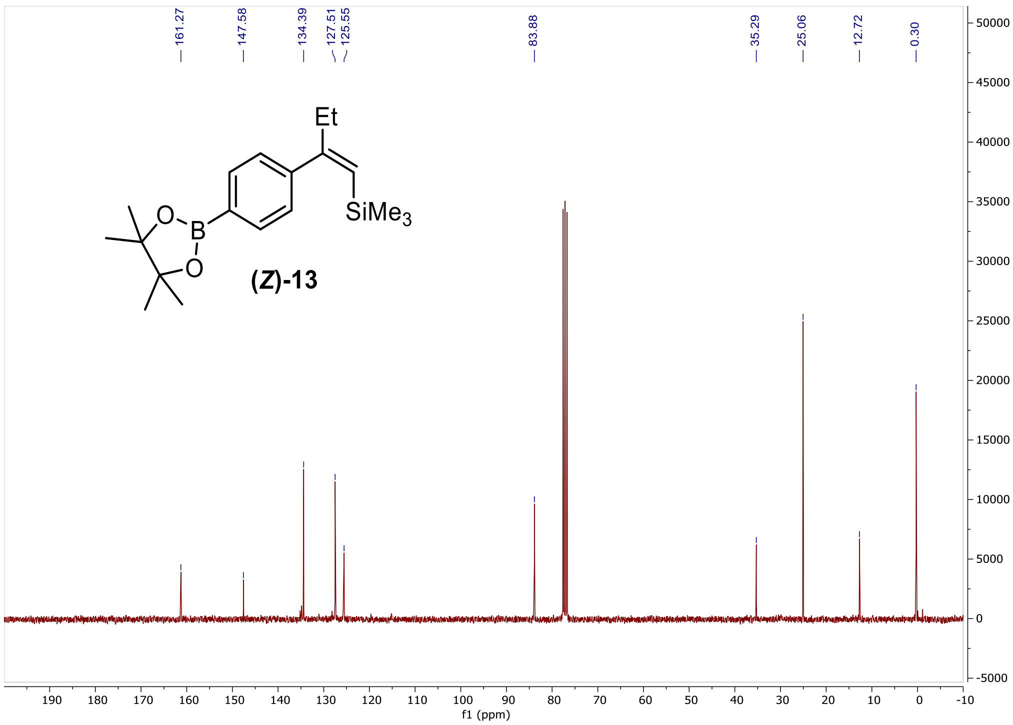

(*Z*)-(2-(4-fluorophenyl)but-1-en-1-yl)trimethylsilane ((*Z*)-14)

^{1}H NMR (300 MHz, $CDCl_3$)

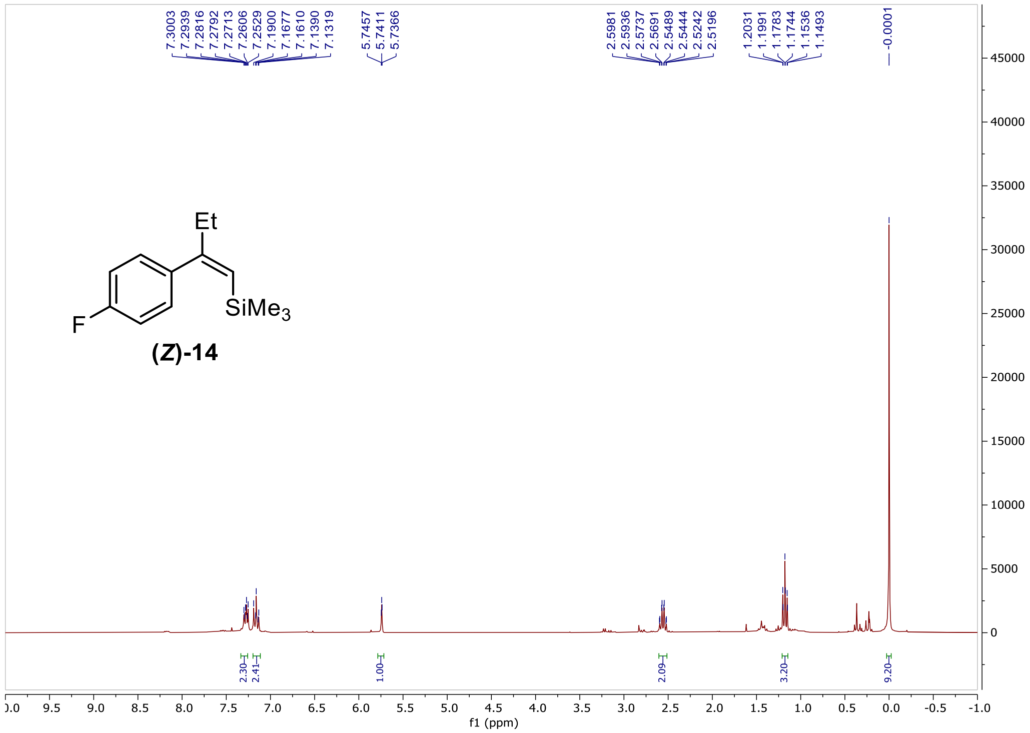

^{13}C NMR (75 MHz, $CDCl_3$)

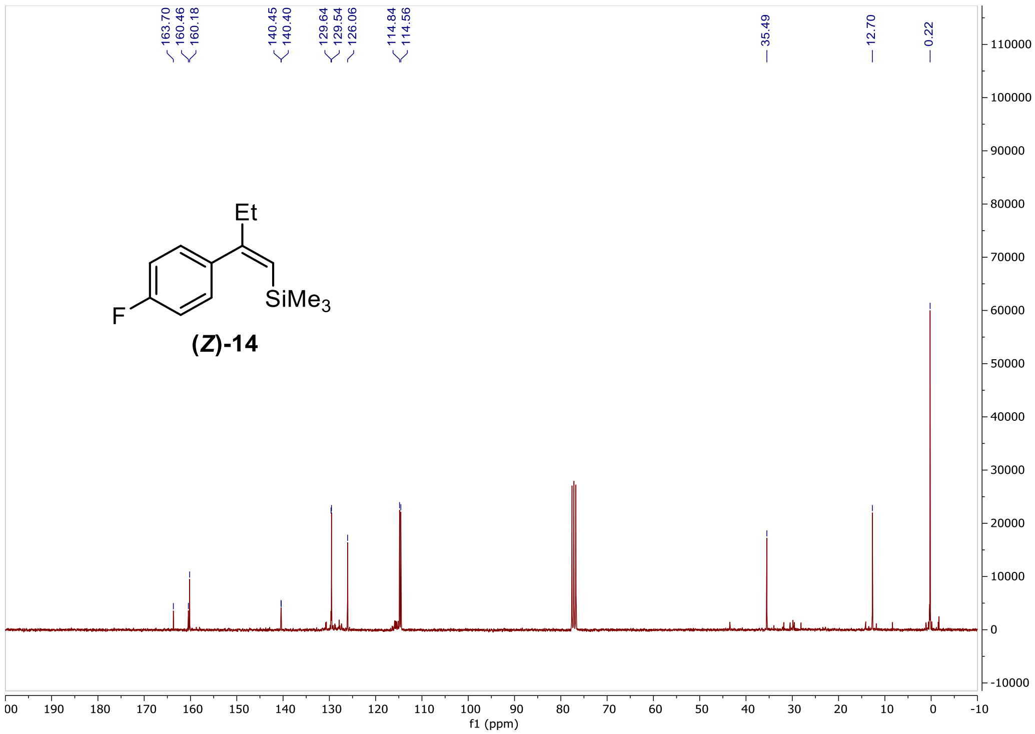

(*Z*)-trimethyl(2-(4-(trifluoromethyl)phenyl)but-1-en-1-yl)silane ((*Z*)-15)

^{1}H NMR (300 MHz, $CDCl_3$)

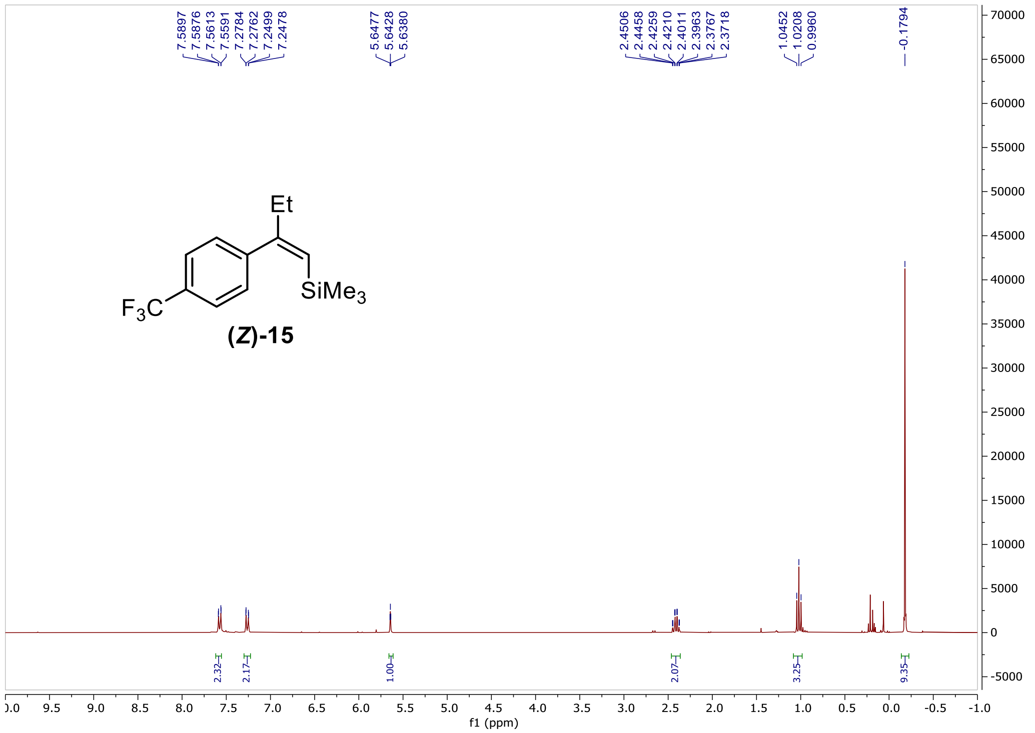

^{13}C NMR (75 MHz, $CDCl_3$)

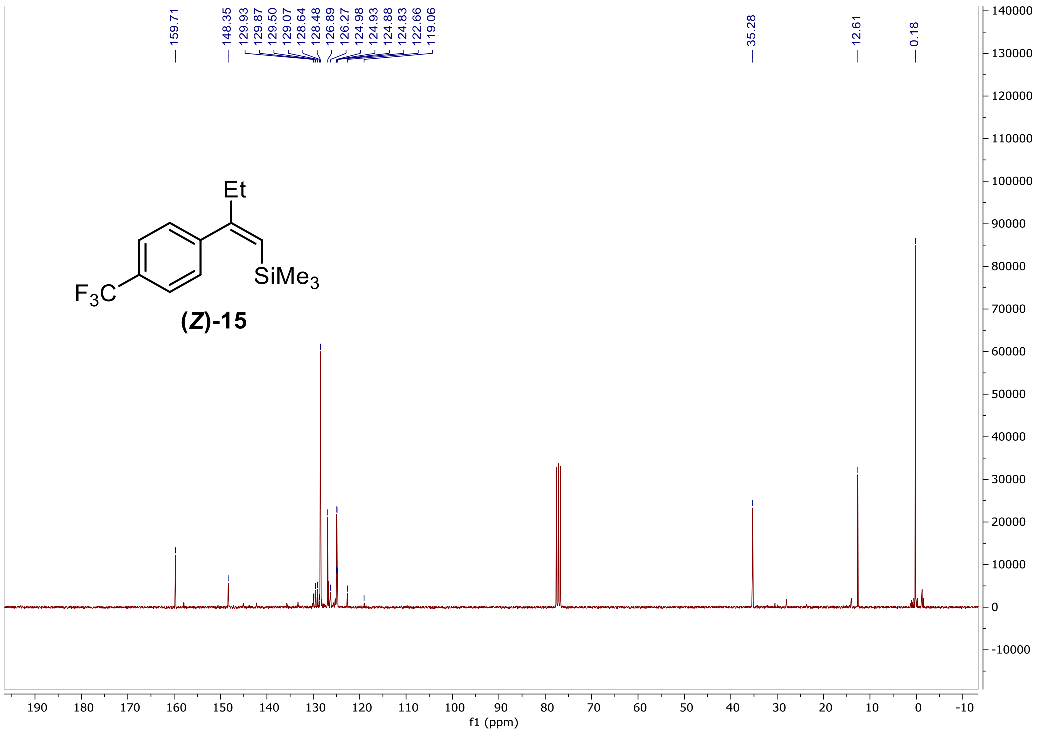

(*Z*)-(2-(4-bromophenyl)but-1-en-1-yl)trimethylsilane ((*Z*)-16)

^{1}H NMR (300 MHz, $CDCl_3$)

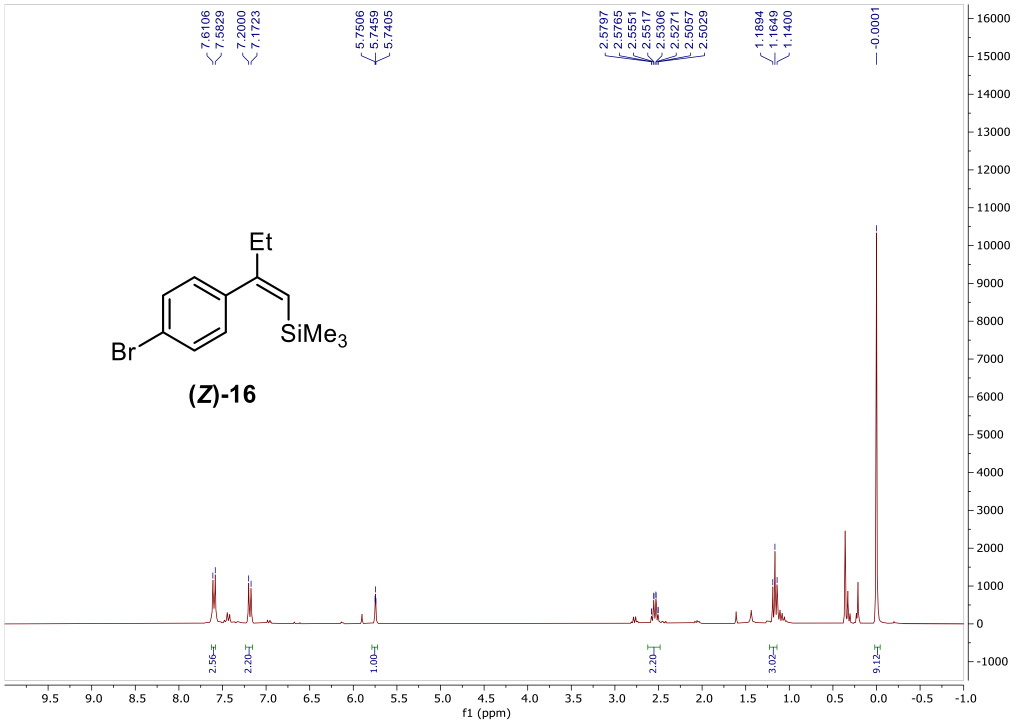

^{13}C NMR (75 MHz, $CDCl_3$)

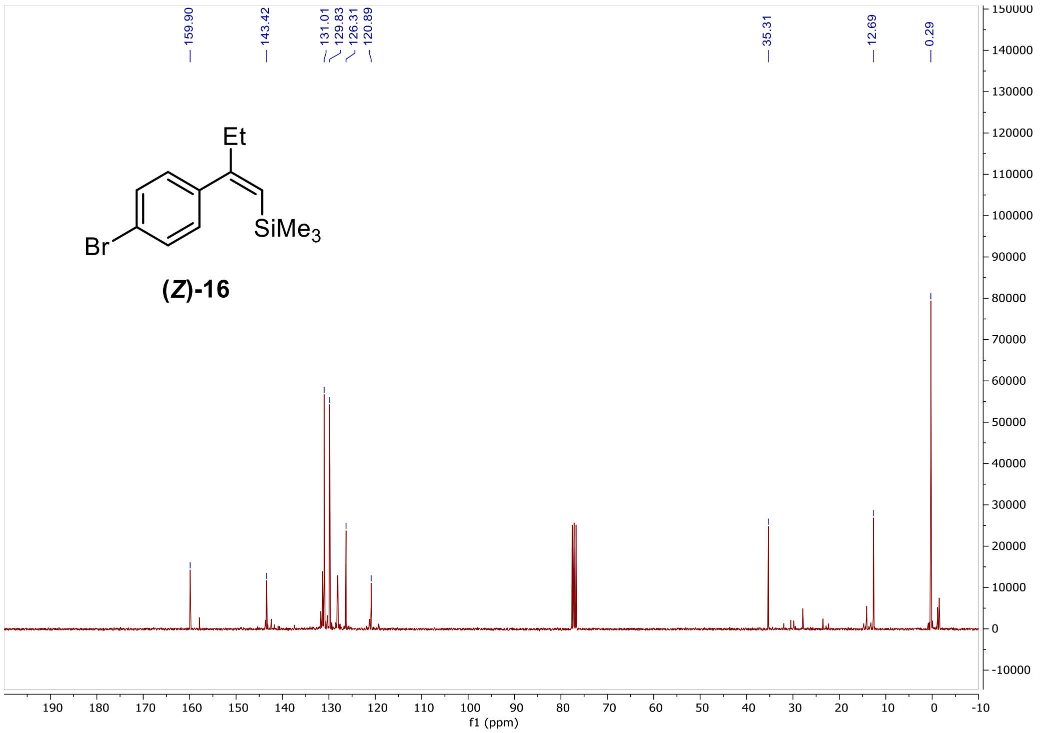

(*Z*)-trimethyl(2-(4-(methylsulfonyl)phenyl)but-1-en-1-yl)silane ((*Z*)-17)

^{1}H NMR (300 MHz, $CDCl_3$)

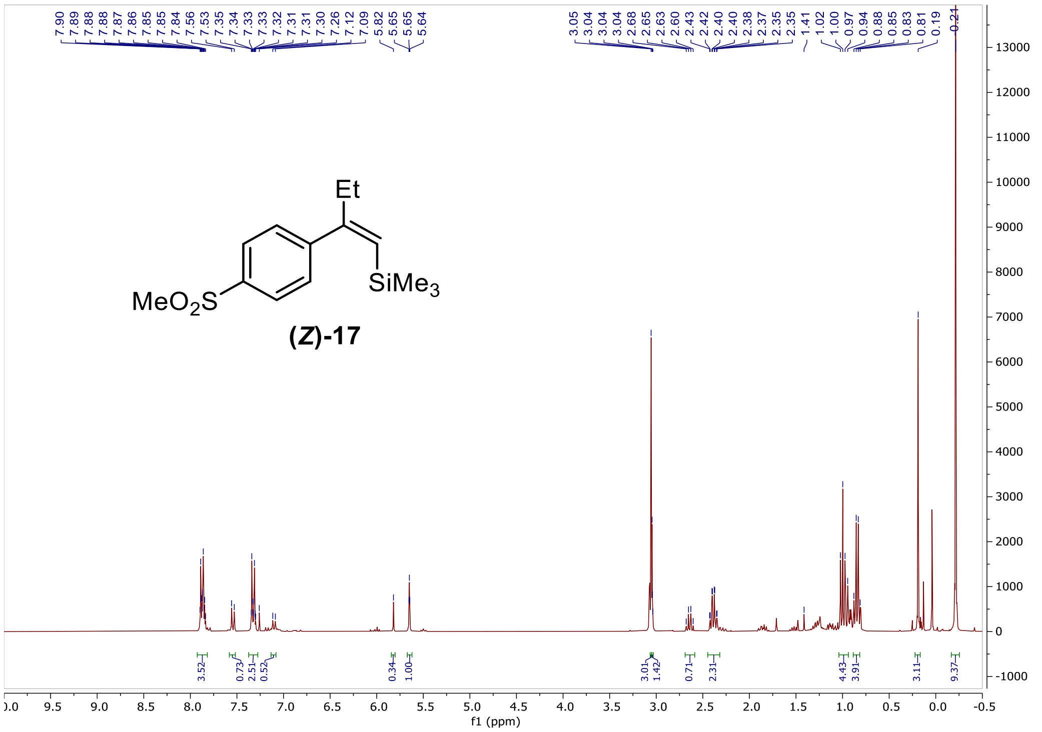

^{13}C NMR (75 MHz, $CDCl_3$)

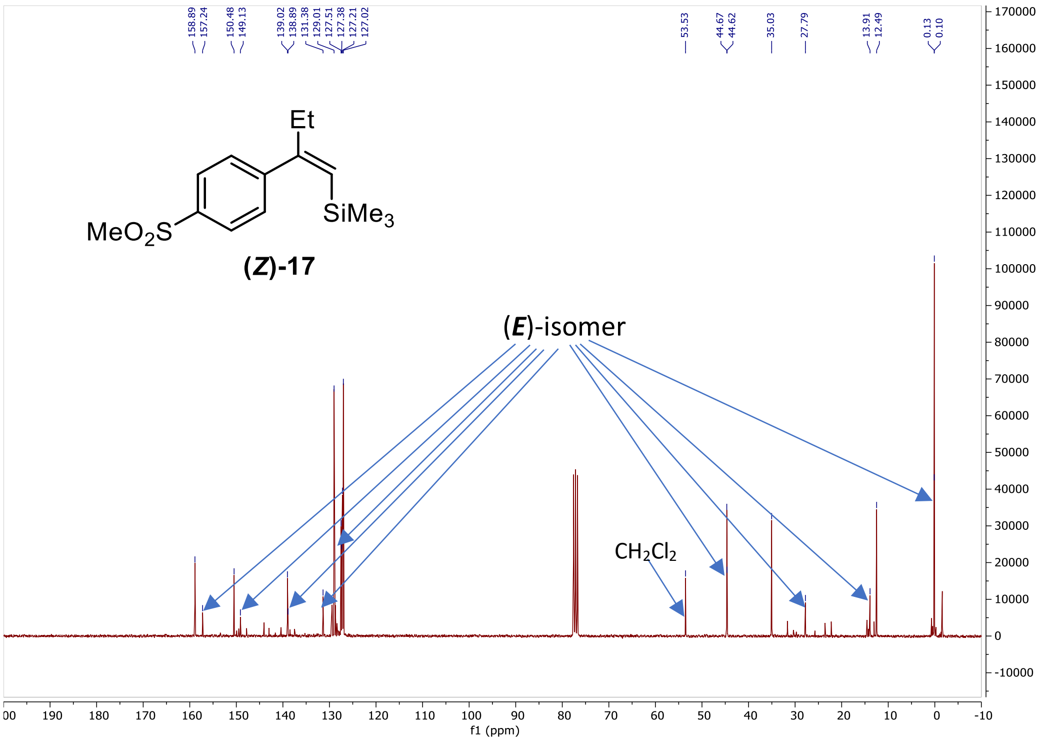

(*Z*)-trimethyl(2-(naphthalen-2-yl)but-1-en-1-yl)silane ((*Z*)-18)

^1H NMR (300 MHz, $CDCl_3$)

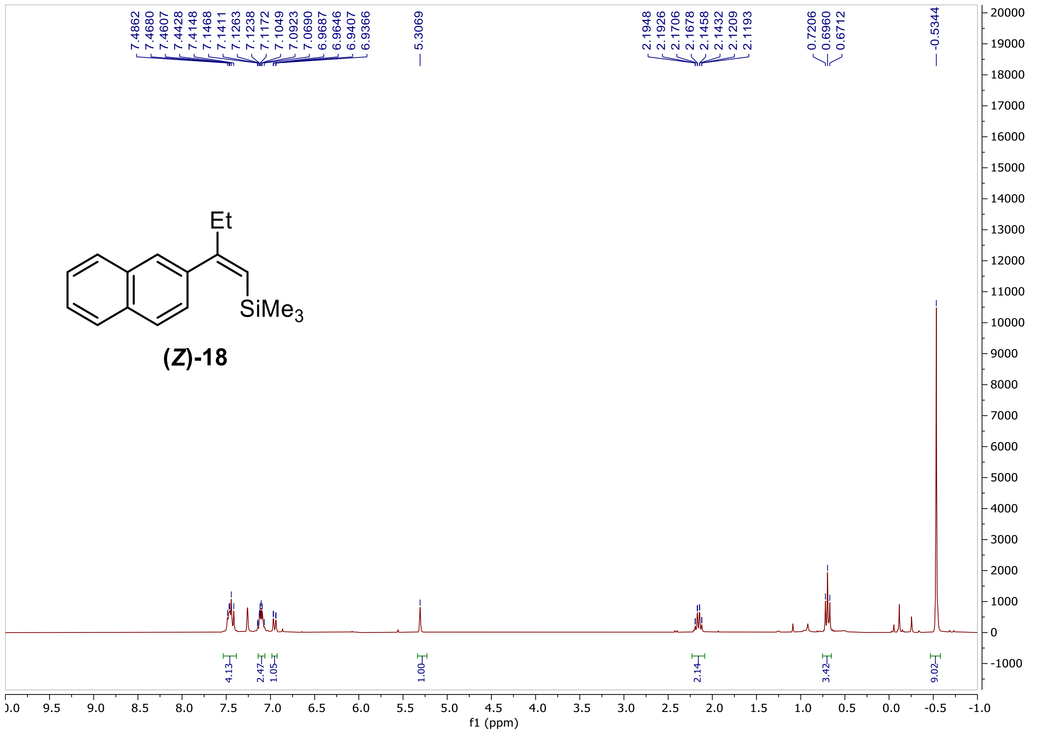

^{13}C NMR (75 MHz, $CDCl_3$)

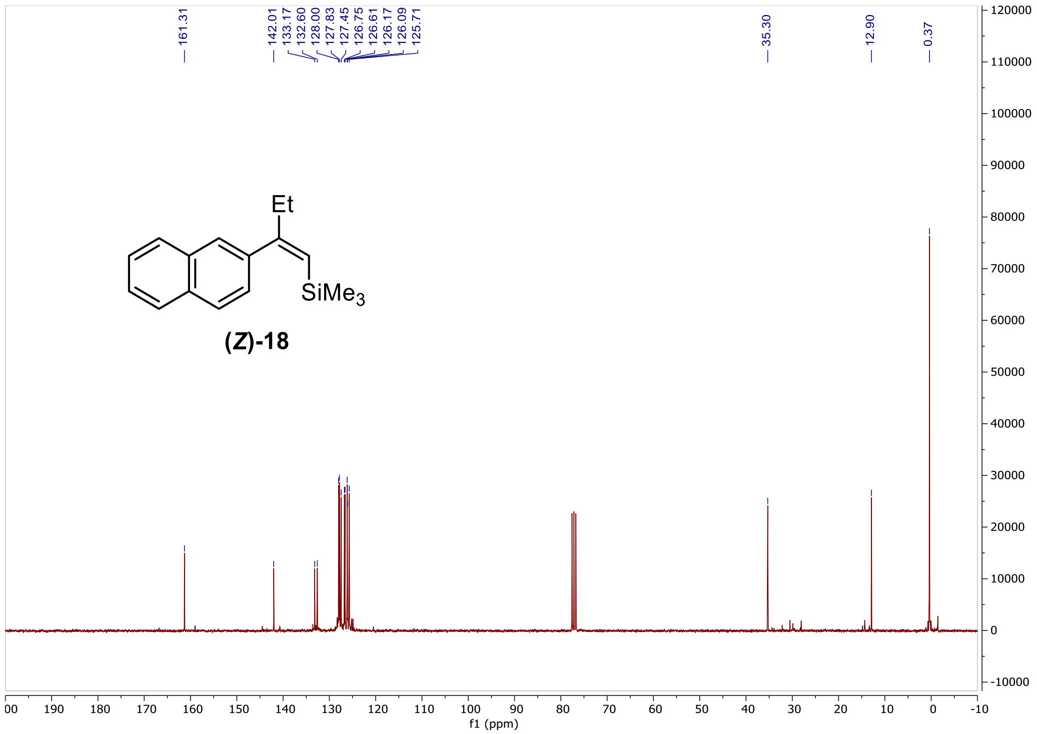

9. GC-FID spectra

(*Z*)-trimethyl(2-phenylbut-1-en-1-yl)silane ((*Z*)-1)

System : GC436 Droite
Method : MARTIN50 250 droite
User : vahine
Volume injecté : 1,000 µl

Acquired : 26/01/2022 15:36:23
Processed : 26/03/2022 09:24:33
Printed : 26/03/2022 09:24:54
Nom colonne : Rt-bDEXsm
Vial : 2

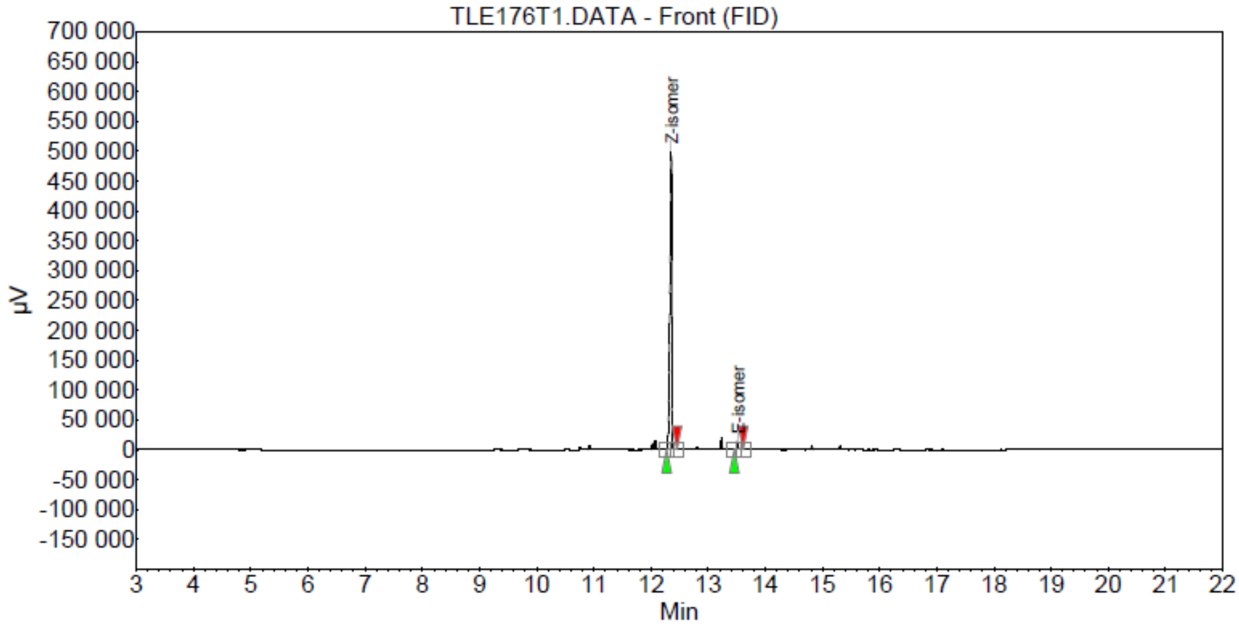

Peak results :

Index	Name	Time [Min]	Quantity [% Area]	Height [µV]	Area [µV.Min]	Area % [%]
1	Z-isomer	12,36	97,78	499568,9	17619,5	97,784
2	E-isomer	13,51	2,22	14159,0	399,4	2,216
Total			100,00	513727,9	18018,9	100,000

(*Z*)-trimethyl(2-phenylprop-1-en-1-yl)silane ((*Z*)-2)

System : GC436 Droite
Method : MARTIN50 250 droite
User : vahine
Volume injecté : 1,000 µl

Acquired : 10/03/2022 07:24:12
Processed : 26/03/2022 10:32:05
Printed : 26/03/2022 10:33:06
Nom colonne : Rt-bDEXsm
Vial : 1

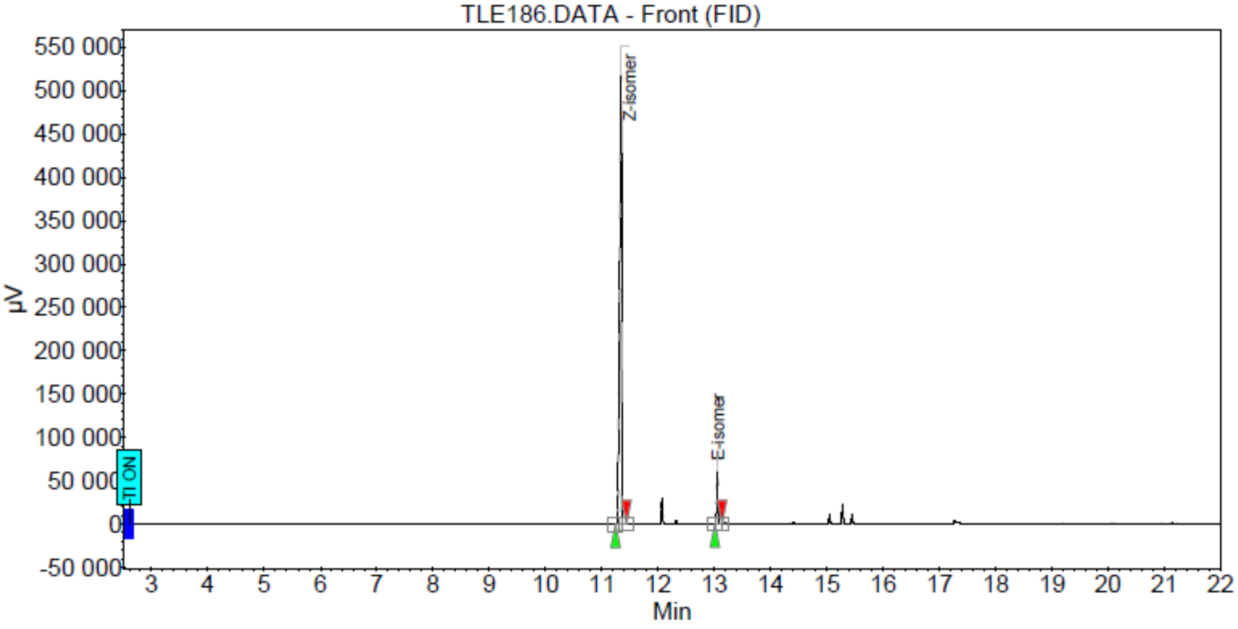

Peak results :

Index	Name	Time [Min]	Area % [%]	Quantity [% Area]	Height [µV]	Area [µV.Min]	As. PE.	Res. HW
1	Z-isomer	11,34	92,743	92,74	518434,1	19013,6	0,62	0,00
2	E-isomer	13,05	7,257	7,26	61251,1	1487,8	0,92	34,71
Total			100,000	100,00	579685,2	20501,4		

(*Z*)-trimethyl(2-phenylpenta-1,4-dien-1-yl)silane ((*Z*)-3)

System : GC436 Droite
Method : MARTIN50 250 droite
User : vahine
Volume injecté : 1,000 µl

Acquired : 09/03/2022 21:12:35
Processed : 11/03/2022 19:37:17
Printed : 26/03/2022 10:00:28
Nom colonne : Rt-bDEXsm
Vial : 5

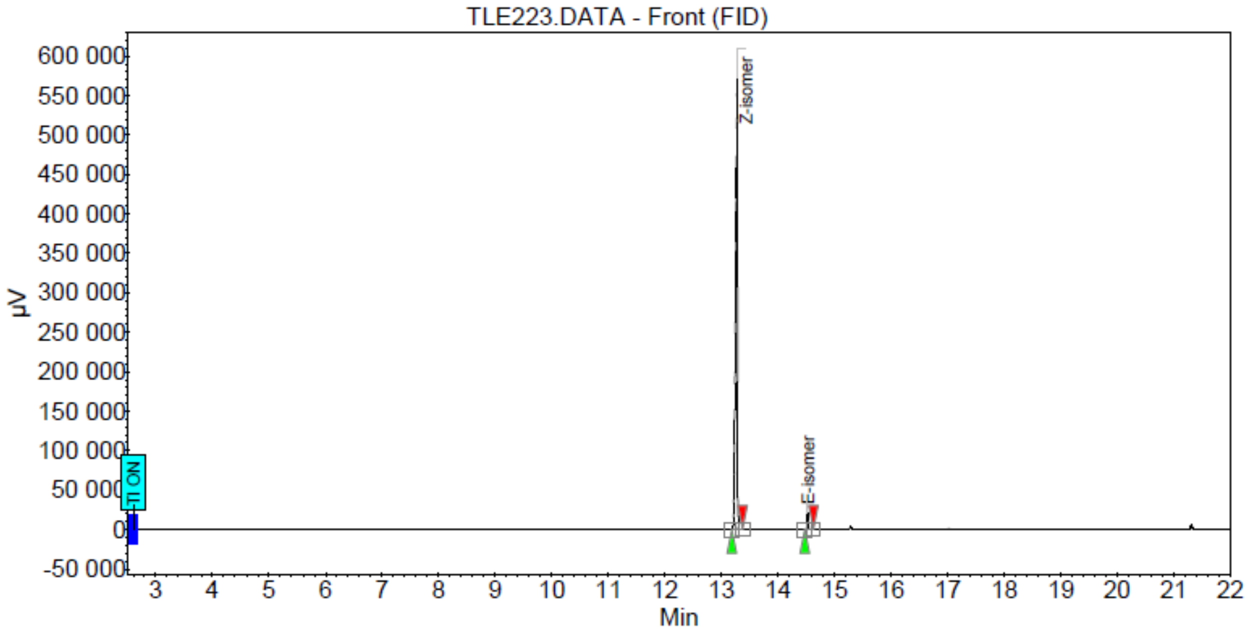

Peak results :

Index	Name	Time [Min]	Area % [%]	Quantity [% Area]	Height [µV]	Area [µV.Min]	As. PE.	Res. HW
1	Z-isomer	13,28	97,754	97,75	572852,5	22576,0	0,60	0,00
2	E-isomer	14,52	2,246	2,25	21321,8	518,8	1,05	24,51
Total			100,000	100,00	594174,3	23094,8		

(E)-trimethyl(2-phenyl-2-(tributylstannyl)vinyl)silane ((*E*)-4)

System : GC436 Droite
Method : MARTIN50 250 droite
User : vahine
Volume injecté : 1,000 µl

Acquired : 27/01/2022 17:37:41
Processed : 26/03/2022 09:55:22
Printed : 26/03/2022 09:55:31
Nom colonne : Rt-bDEXsm
Vial : 6

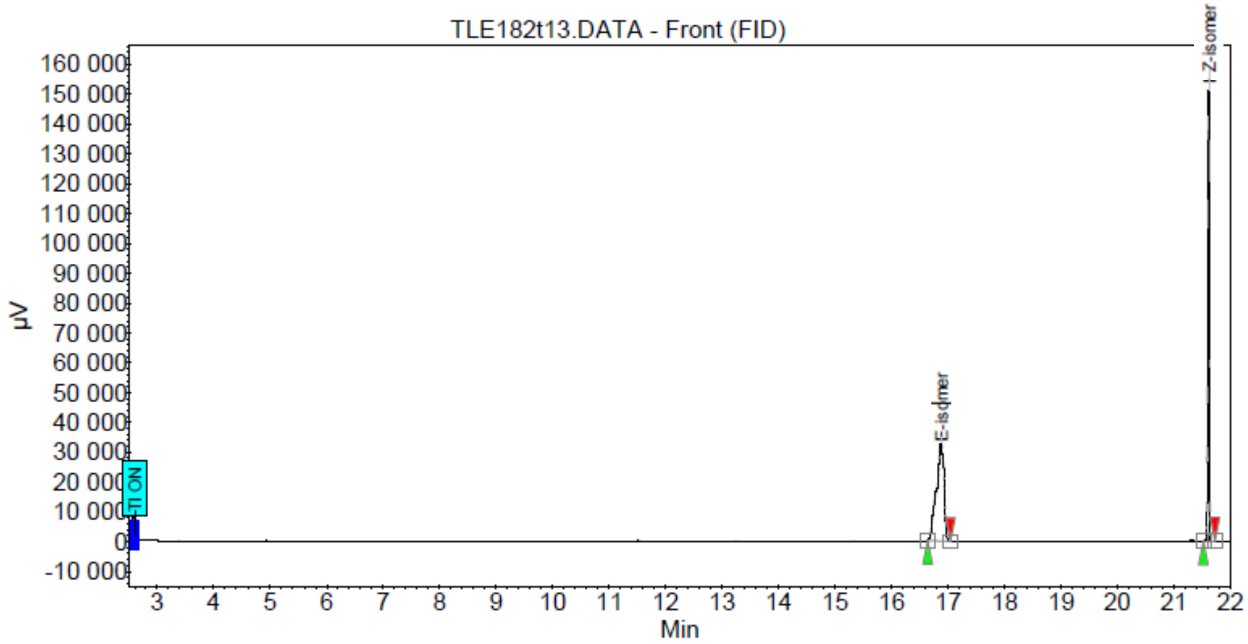

Peak results :

Index	Name	Time [Min]	Area % [%]	Quantity [% Area]	Height [µV]	Area [µV.Min]	As. PE.	Res. HW
1	E-isomer	16,87	56,049	56,05	34815,0	5138,8	0,78	0,00
2	Z-isomer	21,62	43,951	43,95	151093,1	4029,6	0,75	36,44
Total			100,000	100,00	185908,2	9168,4		

(*Z*)-tert-butyldimethyl(styryl)silane ((*Z*)-5)

System : GC436 Droite
Method : MARTIN50 250 droite
User : vahine
Volume injecté : 1,000 µl

Acquired : 09/03/2022 23:00:32
Processed : 11/03/2022 19:38:35
Printed : 11/03/2022 19:38:49
Nom colonne : Rt-bDEXsm
Vial : 9

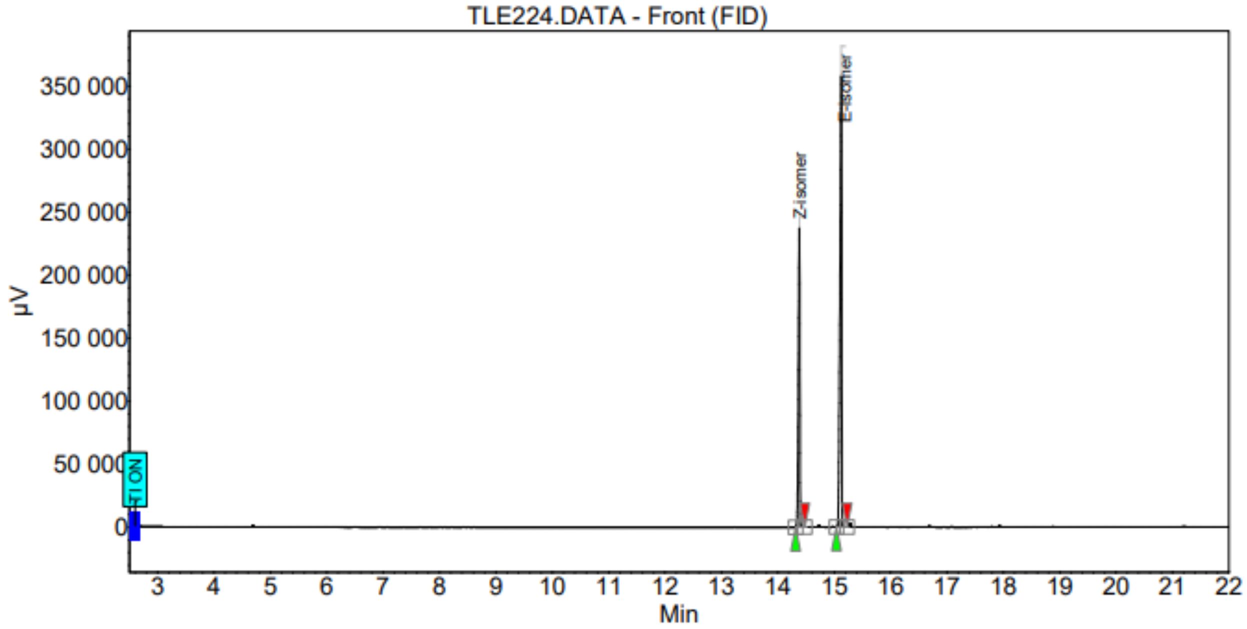

Peak results :

Index	Name	Time [Min]	Area % [%]	Quantity [% Area]	Height [µV]	Area [µV.Min]	As. PE.	Res. HW
1	Z-isomer	14,38	35,053	35,05	237939,4	6623,7	0,71	0,00
2	E-isomer	15,13	64,947	64,95	357740,9	12272,5	0,64	14,96
Total			100,000	100,00	595680,3	18896,2		

(*Z*)-trimethyl(2-(p-tolyl)but-1-en-1-yl)silane ((*Z*)-7)

System : GC436 Droite
Method : MARTIN50 250 droite
User : vahine
Volume injecté : 1,000 µl

Acquired : 26/01/2022 16:30:17
Processed : 26/01/2022 17:57:31
Printed : 26/01/2022 17:59:41
Nom colonne : Rt-bDEXsm
Vial : 4

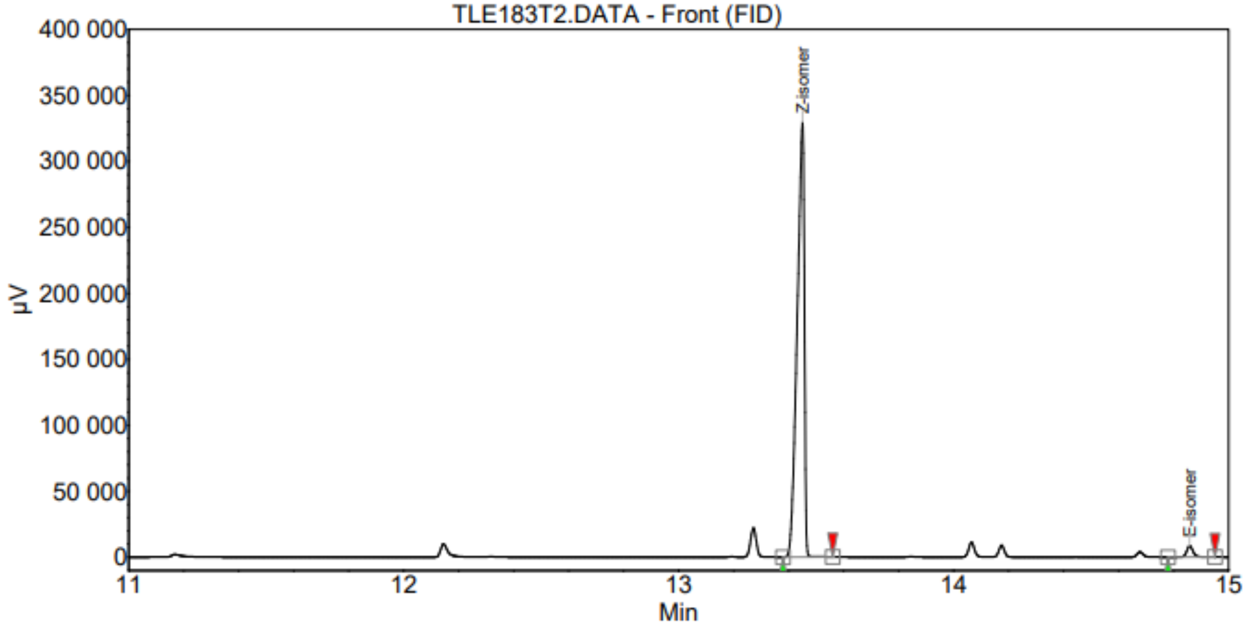

Peak results :

Index	Name	Time [Min]	Quantity [% Area]	Height [µV]	Area [µV.Min]	Area % [%]
1	Z-isomer	13,45	97,75	329818,4	9924,9	97,745
2	E-isomer	14,86	2,25	8631,0	228,9	2,255
Total			100,00	338449,4	10153,8	100,000

(*Z*)-(2-(4-(tert-butyl)phenyl)but-1-en-1-yl)trimethylsilane ((*Z*)-8)

System : GC436 Droite
Method : MARTIN50 250 droite
User : vahine
Volume injecté : 1,000 µl

Acquired : 09/03/2022 17:35:46
Processed : 11/03/2022 19:49:00
Printed : 11/03/2022 19:49:07
Nom colonne : Rt-bDEXsm
Vial : 7

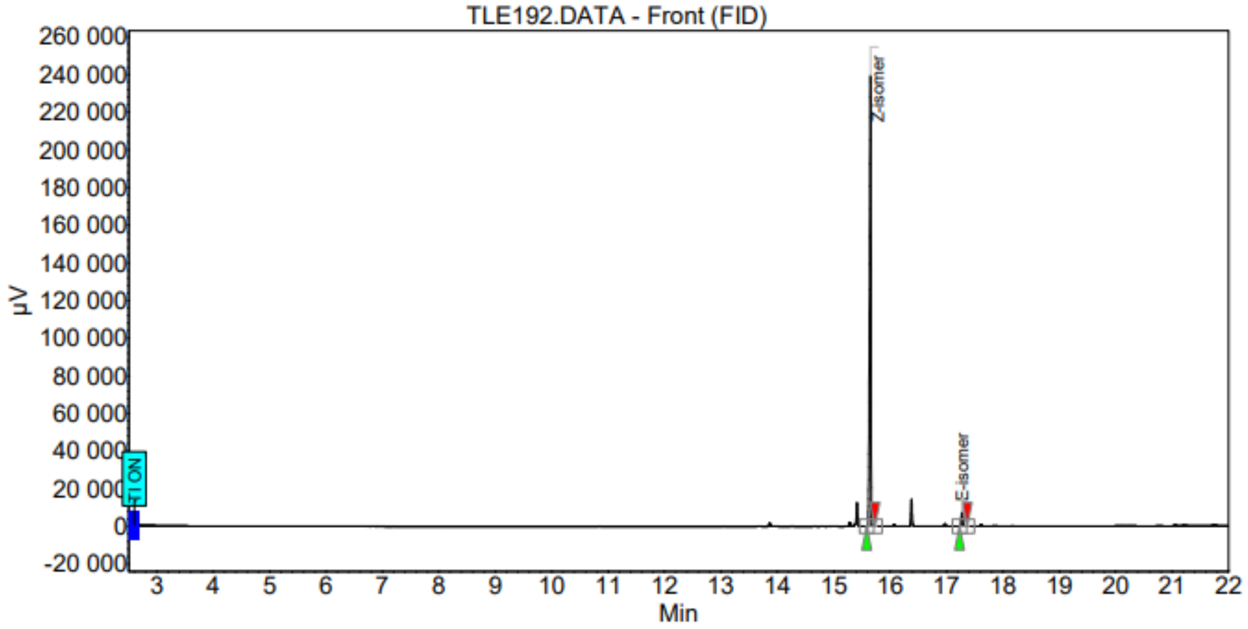

Peak results :

Index	Name	Time [Min]	Area % [%]	Quantity [% Area]	Height [µV]	Area [µV.Min]	As. PE.	Res. HW
1	Z-isomer	15,65	97,267	97,27	239232,9	6681,8	0,72	0,00
2	E-isomer	17,27	2,733	2,73	7687,3	187,7	0,99	39,13
Total			100,000	100,00	246920,2	6869,5		

(*Z*)-trimethyl(2-(m-tolyl)but-1-en-1-yl)silane ((*Z*)-9)

System : GC436 Droite
Method : MARTIN50 250 droite
User : vahine
Volume injecté : 1,000 µl

Acquired : 09/03/2022 15:20:58
Processed : 26/03/2022 09:26:16
Printed : 26/03/2022 09:27:25
Nom colonne : Rt-bDEXsm
Vial : 2

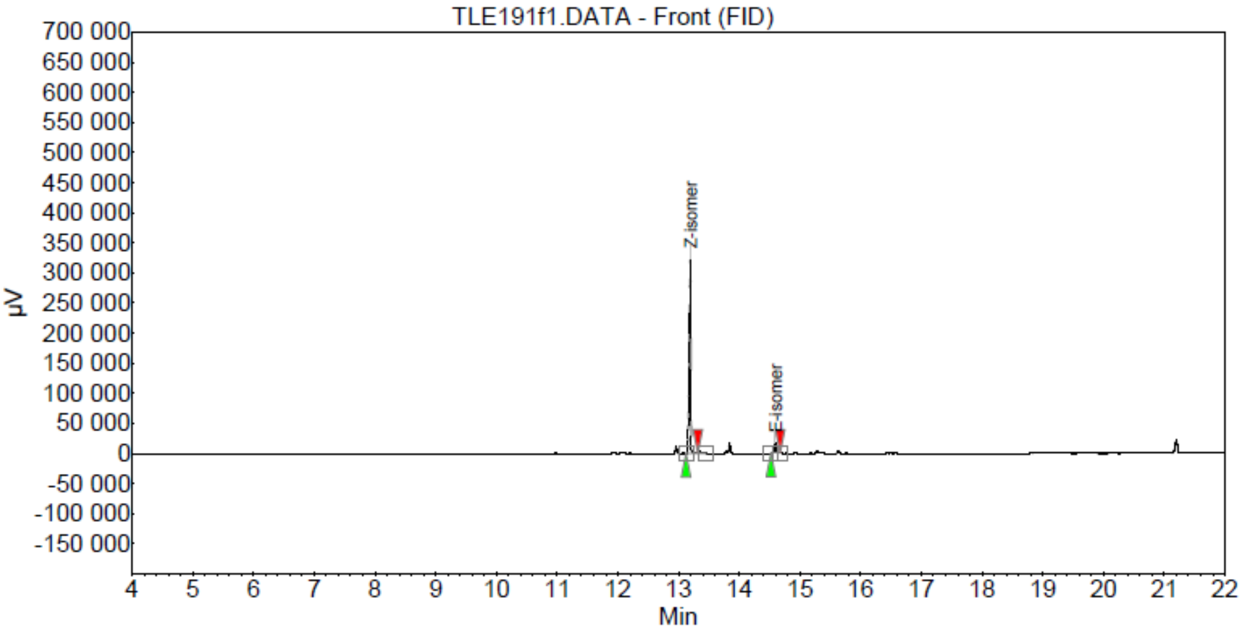

Peak results :

Index	Name	Time [Min]	Quantity [% Area]	Height [µV]	Area [µV.Min]	Area % [%]
1	Z-isomer	13,20	95,77	324760,3	9569,9	95,767
2	E-isomer	14,59	4,23	17429,2	423,0	4,233
Total			100,00	342189,5	9992,9	100,000

(*Z*)-(2-([1,1'-biphenyl]-4-yl)but-1-en-1-yl)trimethylsilane ((*Z*)-10)

System : GC436 Droite
Method : MARTIN50 250 droite
User : vahine
Volume injecté : 1,000 µl

Acquired : 27/01/2022 15:22:33
Processed : 26/03/2022 09:45:52
Printed : 26/03/2022 09:47:15
Nom colonne : Rt-bDEXsm
Vial : 1

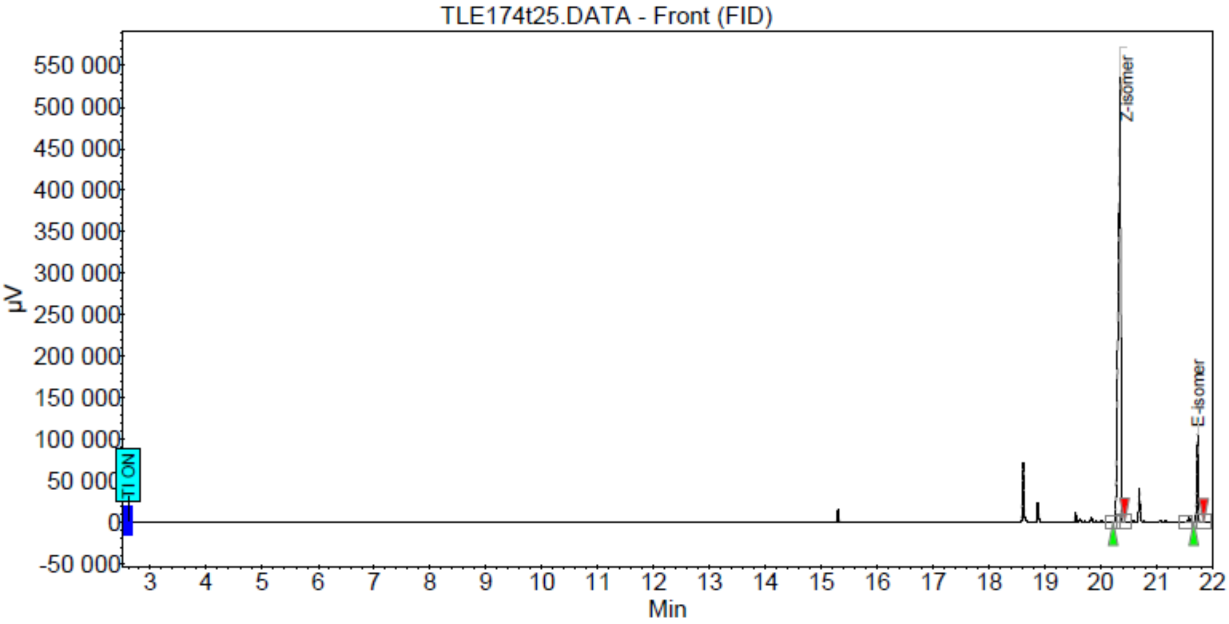

Peak results :

Index	Name	Time [Min]	Area % [%]	Quantity [% Area]	Height [µV]	Area [µV.Min]	As. PE.	Res. HW
1	Z-isomer	20,35	89,939	89,94	537473,4	26172,6	0,58	0,00
2	E-isomer	21,73	10,061	10,06	105613,9	2927,9	0,77	21,76
Total			100,000	100,00	643087,3	29100,6		

(*Z*)-trimethyl(2-(4-(methylthio)phenyl)but-1-en-1-yl)silane ((*Z*)-11)

System : GC436 Droite
Method : MARTIN50 250 droite
User : vahine
Volume injecté : 1,000 µl

Acquired : 09/03/2022 18:29:46
Processed : 11/03/2022 19:47:31
Printed : 11/03/2022 19:47:42
Nom colonne : Rt-bDEXsm
Vial : 9

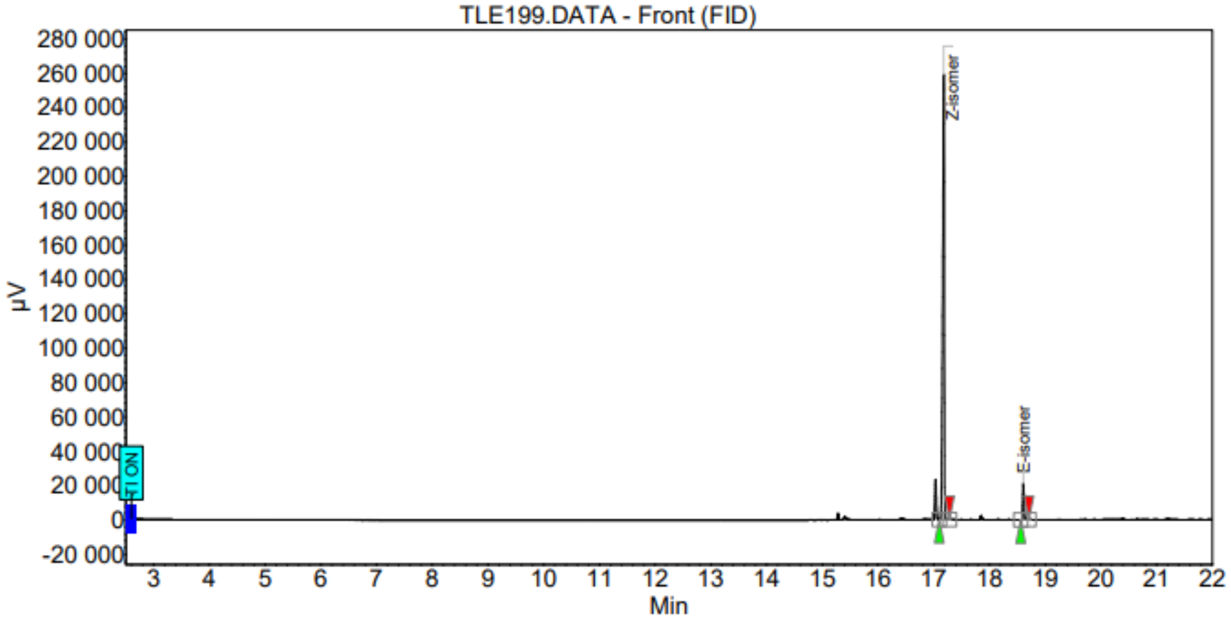

Peak results :

Index	Name	Time [Min]	Area % [%]	Quantity [% Area]	Height [µV]	Area [µV.Min]	As. PE.	Res. HW
1	Z-isomer	17,18	93,778	93,78	258931,8	8081,2	0,68	0,00
2	E-isomer	18,61	6,222	6,22	21398,0	536,2	1,01	32,36
Total			100,000	100,00	280329,8	8617,4		

(*Z*)-trimethyl(2-(4-(trifluoromethoxy)phenyl)but-1-en-1-yl)silane ((*Z*)-12)

System : GC436 Droite
Method : MARTIN50 250 droite
User : vahine
Volume injecté : 1,000 µl

Acquired : 27/01/2022 18:58:50
Processed : 26/03/2022 09:39:37
Printed : 26/03/2022 09:40:25
Nom colonne : Rt-bDEXsm
Vial : 10

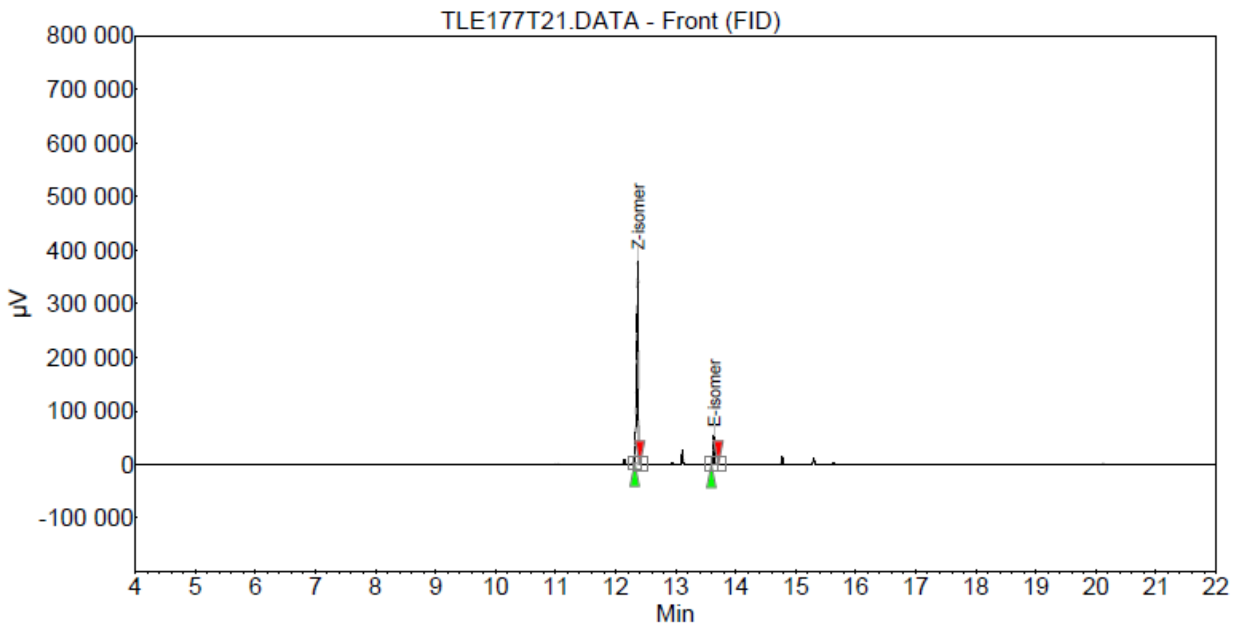

Peak results :

Index	Name	Time [Min]	Quantity [% Area]	Height [µV]	Area [µV.Min]	Area % [%]
2	Z-isomer	12,37	90,33	379635,2	11677,5	90,332
1	E-isomer	13,64	9,67	54731,9	1249,8	9,668
Total			100,00	434367,1	12927,2	100,000

(*Z*)-trimethyl(2-(4-(4,4,5,5-tetramethyl-1,3,2-dioxaborolan-2-yl)phenyl)but-1-en-1-yl)silane ((*Z*)-13)

System : GC436 Droite
Method : MARTIN50 250 droite
User : vahine
Volume injecté : 1,000 µl

Acquired : 27/01/2022 11:44:37
Processed : 26/03/2022 09:44:41
Printed : 26/03/2022 09:44:48
Nom colonne : Rt-bDEXsm
Vial : 6

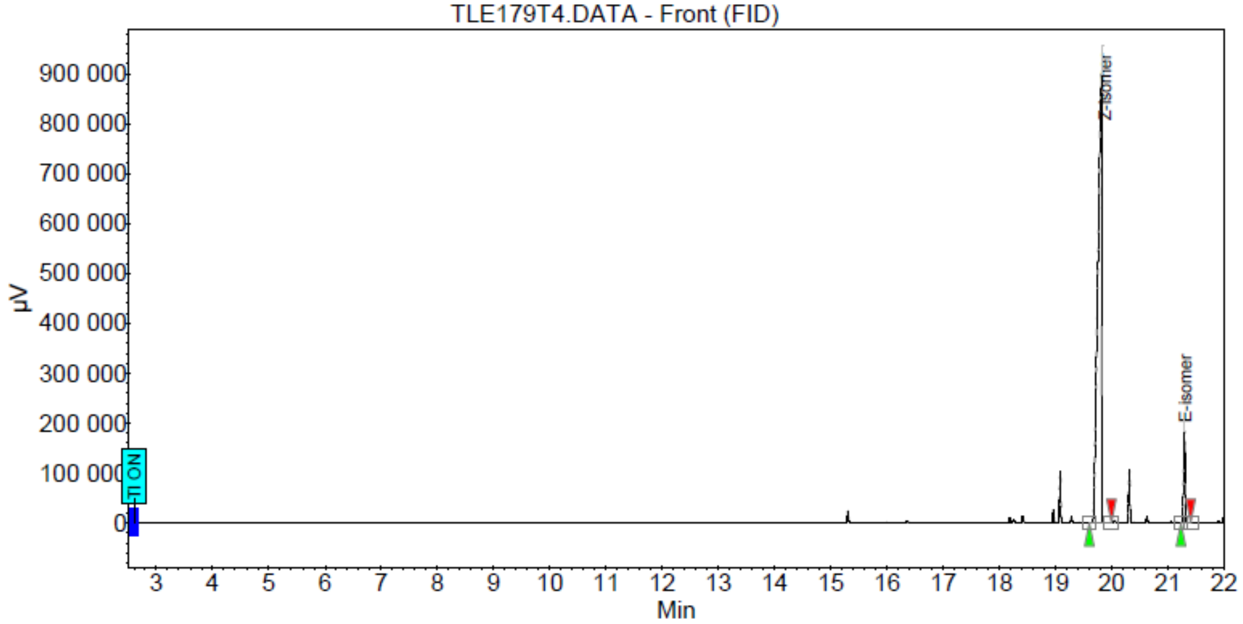

Peak results :

Index	Name	Time [Min]	Area % [%]	Quantity [% Area]	Height [µV]	Area [µV.Min]	As. PE.	Res. HW
1	Z-isomer	19,82	92,917	92,92	899315,8	70292,6	0,53	0,00
2	E-isomer	21,30	7,083	7,08	182687,9	5358,2	0,71	16,16
Total			100,000	100,00	1082003,6	75650,8		

(*Z*)-trimethyl(2-(4-(trifluoromethyl)phenyl)but-1-en-1-yl)silane ((*Z*)-15)

System : GC436 Droite
Method : MARTIN50 250 droite
User : vahine
Volume injecté : 1,000 µl

Acquired : 26/01/2022 17:51:23
Processed : 26/03/2022 09:28:41
Printed : 26/03/2022 09:28:54
Nom colonne : Rt-bDEXsm
Vial : 7

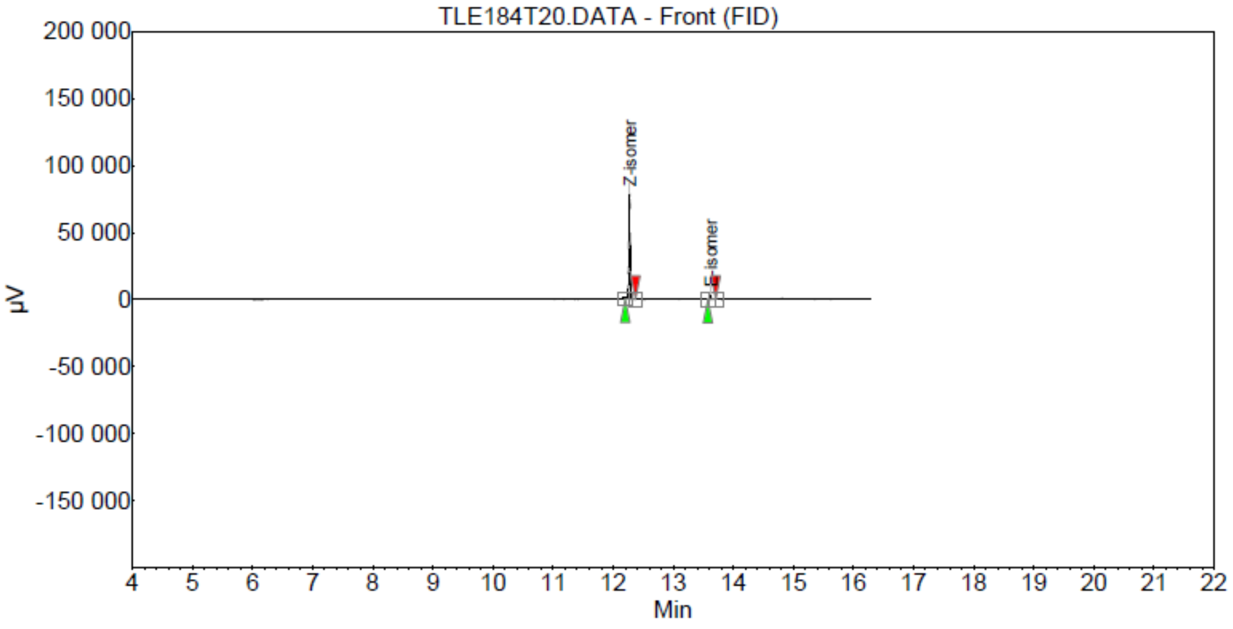

Peak results :

Index	Name	Time [Min]	Quantity [% Area]	Height [µV]	Area [µV.Min]	Area % [%]
1	Z-isomer	12,27	94,28	78648,5	1818,3	94,283
2	E-isomer	13,61	5,72	3792,7	110,3	5,717
Total			100,00	82441,1	1928,5	100,000

(*Z*)-(2-(4-bromophenyl)but-1-en-1-yl)trimethylsilane ((*Z*)-16)

System : GC436 Droite
Method : MARTIN50 250 droite
User : vahine
Volume injecté : 1,000 µl

Acquired : 27/01/2022 10:50:30
Processed : 26/03/2022 09:43:08
Printed : 26/03/2022 09:43:15
Nom colonne : Rt-bDEXsm
Vial : 4

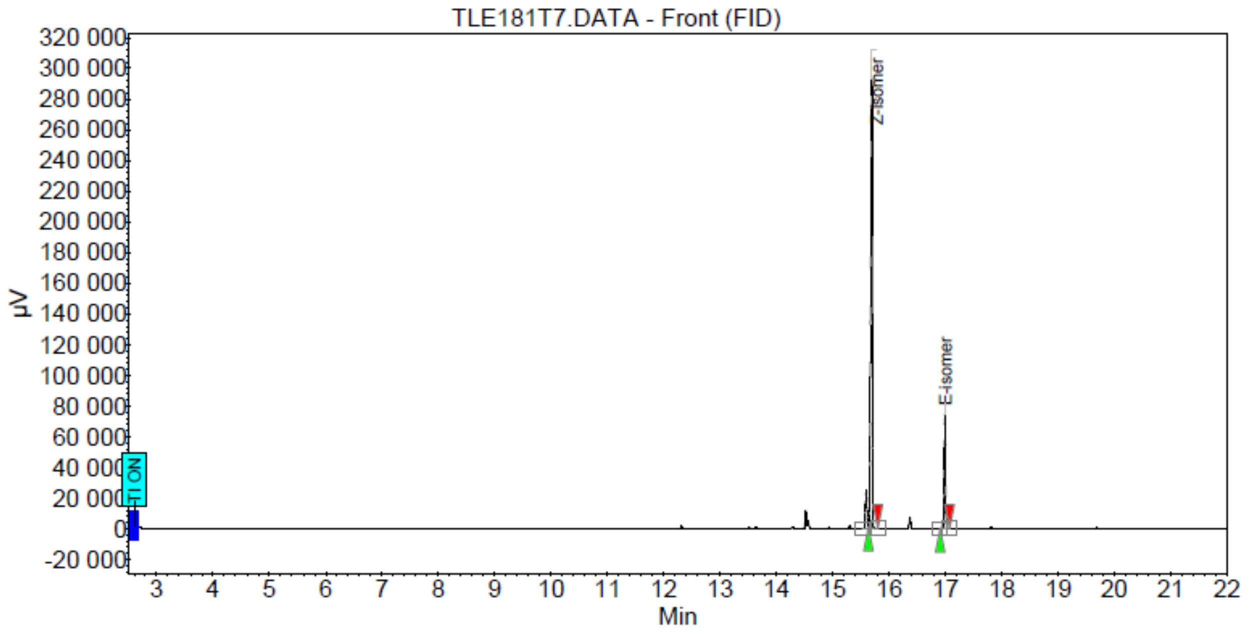

Peak results :

Index	Name	Time [Min]	Area % [%]	Quantity [% Area]	Height [µV]	Area [µV.Min]	As. PE.	Res. HW
1	Z-isomer	15,70	83,317	83,32	293236,3	9589,9	0,65	0,00
2	E-isomer	16,99	16,683	16,68	74913,0	1920,3	0,86	27,88
Total			100,000	100,00	368149,3	11510,2		

(*Z*)-trimethyl(2-(4-(methylsulfonyl)phenyl)but-1-en-1-yl)silane ((*Z*)-17)

System : GC436 Droite
Method : MARTIN50 250 droite
User : vahine
Volume injecté : 1,000 µl

Acquired : 09/03/2022 20:18:41
Processed : 11/03/2022 19:27:41
Printed : 11/03/2022 19:29:31
Nom colonne : Rt-bDEXsm
Vial : 3

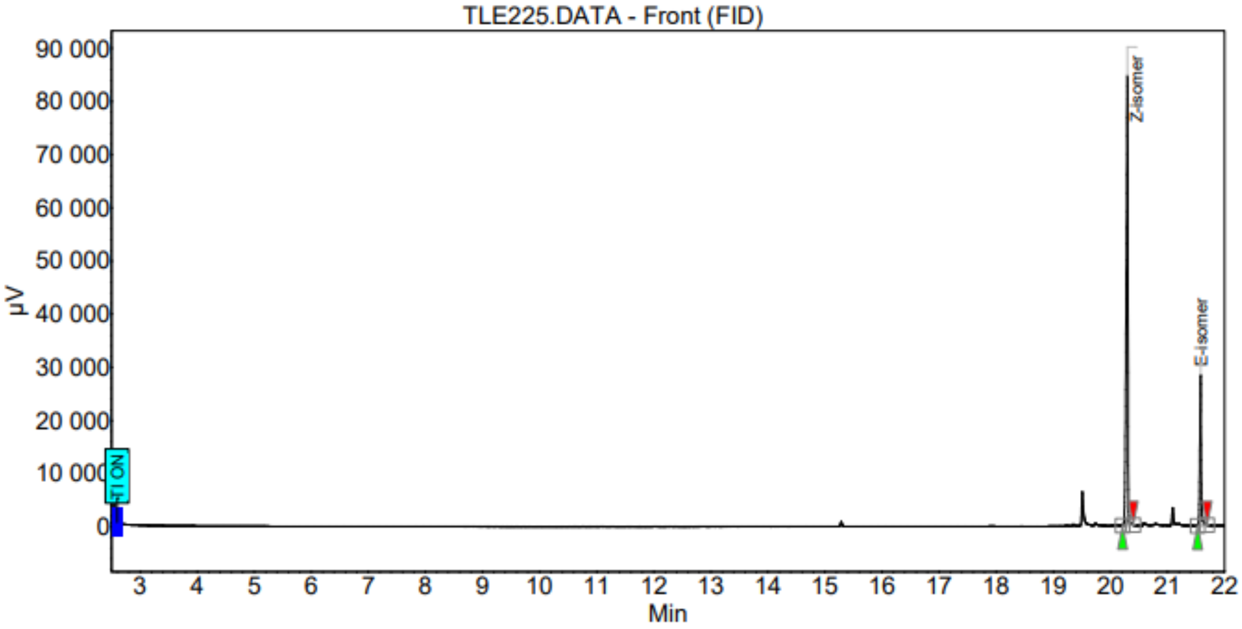

Peak results :

Index	Name	Time [Min]	Area % [%]	Quantity [% Area]	Height [µV]	Area [µV.Min]	As. PE.	Res. HW
1	Z-isomer	20,30	78,821	78,82	84553,2	2897,8	0,69	0,00
2	E-isomer	21,58	21,179	21,18	28398,5	778,6	1,06	26,44
Total			100,000	100,00	112951,7	3676,4		

(*Z*)-trimethyl(2-(naphthalen-2-yl)but-1-en-1-yl)silane ((*Z*)-18)

System : GC436 Droite
Method : MARTIN50 250 droite
User : vahine
Volume injecté : 1,000 µl

Acquired : 27/01/2022 16:16:34
Processed : 26/03/2022 09:48:01
Printed : 26/03/2022 09:48:54
Nom colonne : Rt-bDEXsm
Vial : 3

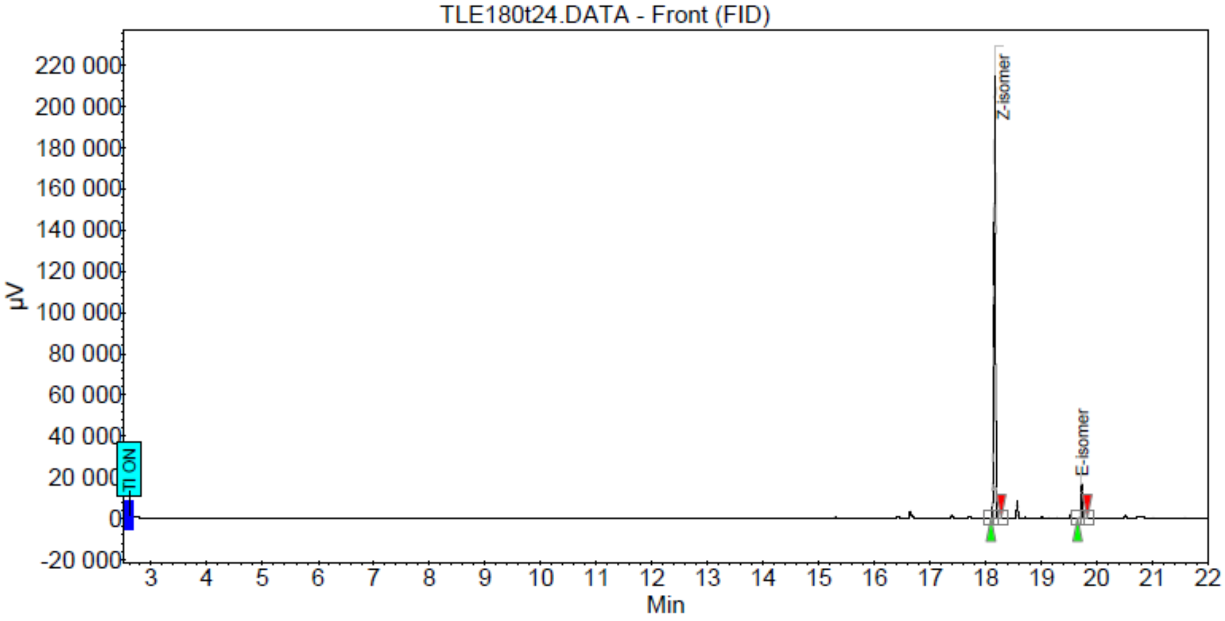

Peak results :

Index	Name	Time [Min]	Area % [%]	Quantity [% Area]	Height [µV]	Area [µV.Min]	As. PE.	Res. HW
1	Z-isomer	18,17	93,642	93,64	215384,6	6551,5	0,71	0,00
2	E-isomer	19,73	6,358	6,36	16644,1	444,9	1,10	34,58
Total			100,000	100,00	232028,8	6996,4		
